# Exploration of the role of drug resistance-associated anoikis-related genes in HER2-Negative breast cancer through bioinformatics analysis

**DOI:** 10.1016/j.bbrep.2025.101947

**Published:** 2025-02-21

**Authors:** Yuanzhao Wu, Cong Chen, Zao Jin, Kesi Zheng

**Affiliations:** Department of Breast and Thyroid Surgery, Wenzhou People's Hospital, The Third Clinical Institute Affiliated to Wenzhou Medical University, Wenzhou, 325000, People's Republic of China

**Keywords:** HER2 negative breast cancer, Prognostic models, anoikis, Biomarkers, Immune microenvironment

## Abstract

**Aims:**

To explore the potential functions and impacts of anoikis-related genes (ARGs) in breast cancer chemotherapy and to construct a prognosis model for HER2-negative breast cancer (HNBC) based on drug resistance-related ARGs.

**Background:**

Breast cancer remains a leading cause of cancer-related mortality, with HER2-negative subtypes exhibiting high rates of metastasis and recurrence. Standard treatments for HNBC include taxane- and anthracycline-based chemotherapies, which aim to mitigate recurrence and metastasis. Anoikis, a specialized form of programmed cell death, plays a pivotal role in maintaining tissue homeostasis by eliminating detached cells. Cancer cells often develop resistance to anoikis, enabling survival in adverse conditions and promoting tumor progression.

**Objective:**

To investigate the intersection of breast cancer drug resistance-related genes and anoikis-related genes (ARGs) and to assess their potential as biomarkers for HNBC. The study also aims to analyze differences in immune microenvironment and drug sensitivity among different prognosis score groups.

**Method:**

A bioinformatics approach was employed to identify the intersection of breast cancer drug resistance-related genes and ARGs. A prognosis model for HNBC was developed based on these identified drug resistance-related ARGs. The study further examined differences in the immune microenvironment and drug sensitivity among different prognosis score groups.

**Result:**

A prognosis model for HNBC was successfully constructed based on drug resistance-related ARGs. The study identified significant differences in immune microenvironment and drug sensitivity across different prognosis score groups.

**Conclusion:**

The findings suggest that ARGs could be key in tailoring more effective therapeutic approaches for patients with HER2-negative breast cancer.

## Highlights

• Identified anoikis-related genes (ARGs) linked to chemotherapy resistance in HER2-negative breast cancer (HNBC).
• Created a prognostic model for HNBC using drug resistance-related ARGs, enabling improved risk stratification.
• Found significant immune microenvironment variations across prognosis score groups.
• Noted distinct drug sensitivity patterns among prognosis score groups, guiding personalized therapy.
• ARGs emerged as potential biomarkers, advancing personalized treatment strategies for HNBC.

## 1 Introduction

Human epidermal growth factor receptor-2 (HER2) negative breast cancer (HNBC) poses a high risk of distant metastasis or recurrence [1,2]. Current approaches include taxane- and anthracycline-based chemotherapies, which have shown efficacy in reducing recurrence and metastasis rates [3,4]. Despite these advances, chemotherapy resistance remains a major challenge, limiting treatment outcomes and patient survival [5,6].

Anoikis, or programmed cell death, is a form of cell death involving a series of complex molecular events, including activation of proteases, and clearance of dead cells [7]. The interaction between death receptors on the cell surface and their corresponding ligands initiates a cascade of signaling events that culminate in cell anoikis. During this process, alterations in mitochondrial membrane permeability and the subsequent release of mitochondrial proteins drive the cell toward anoikis. This mechanism is essential for physiological functions such as maintaining tissue homeostasis, eliminating damaged cells, and preventing abnormal cell proliferation [8]. Cancer cells undergo various changes in the tumor microenvironment, including alterations in the extracellular matrix and disruption of cell-cell interactions. These changes may lead to resistance of cells to anoikis, promoting tumor growth and invasion. In recent years, many scholars have explored the application of anoikis-related genes (ARGs) in HNBC. Liu et al. found that TJP3 in ARGs could promote breast cancer T-cell immune escape and chemotherapy resistance [9]. Tang et al. constructed a prognostic model based on ARGs to predict the prognosis of HNBC patients and achieved favorable predictive results [10]. However, there is currently no research exploring the possible role of resistance-related ARGs' abnormal expression in HNBC.

Therefore, this study first investigates the potential functions or impacts of ARGs in chemotherapy resistance in breast cancer and identifies the intersection of resistance-related genes and ARGs in HNBC. Subsequently, a prognostic model for HNBC is constructed based on resistance-related ARGs, and the differences in the immune microenvironment and drug sensitivity among different prognostic score groups are explored. We aim to provide more precise and personalized treatment options for HER2-negative breast cancer and discuss the feasibility of ARGs as potential biomarkers.

## 2 Method

### 2.1 Data acquisition and preprocessing

The study began by searching for "anoikis" in the Genecard database (https://www.genecards.org/), selecting genes with a Score exceeding 0.5 as Anoikis-Related Genes (ARGs), resulting in the identification of 503 ARGs. Following this, two transcriptome datasets specific to triple-negative breast cancer (HNBC), namely GSE25055 and GSE25065, were retrieved from the Gene Expression Omnibus (GEO) database. GSE25055, comprising 310 HNBC samples treated with neoadjuvant taxane-anthracycline chemotherapy (197 insensitive and 113 sensitive to chemotherapy), was designated as the training set. GSE25065, with 198 HNBC samples subjected to paclitaxel-anthracycline chemotherapy (142 insensitive and 56 sensitive to chemotherapy), served as the test set.

### 2.2 Differential expression and gene correlation analysis

Differentially Expressed Genes (DEGs) between the drug-resistant (RR) and drug-sensitive (RS) HNBC groups were identified using the empirical Bayesian method provided by the "limma" package in R. A significance threshold was applied, with adjusted P-values set at < 0.05 and |logFC| > 0.3. Spearman correlation analysis was conducted on intersecting genes, and the chromosomal distribution of these genes was visualized using the "circlize" package in R. Additionally, Copy Number Variation (CNV) data for intersecting genes were sourced from the UCSC Xena database (https://xena.ucsc.edu/) and visualized. Enrichment analysis for prognostic genes was performed using the Metascape database (https://www.metascape.org/gp/index.html).

### 2.3 Construction and validation of the prognostic model

To assess genes significantly associated with prognosis among intersecting genes, univariate Cox regression analysis was performed first. The prognostic model was then developed using multivariate Cox regression and LASSO-Cox regression through the "glmnet" package in R. The model's effectiveness in predicting HNBC prognosis was evaluated by comparing Distant Metastasis-Free Survival (DRFS) between high-risk and low-risk groups using Kaplan-Meier survival curves, generated with the "survival" package in R. DRFS was defined as the duration from the initial biopsy to the diagnosis of distant metastasis or death from any cause. The "timeROC" package in R was used to evaluate the model's predictive power for 1-year, 3-year, and 5-year survival outcomes in HNBC samples.

### 2.4 Nomogram model construction

A nomogram was constructed to predict 1-year, 3-year, and 5-year DRFS by integrating risk scores with clinical features (age, gender, and staging) using the "rms" package in R. Calibration curves were employed to assess the nomogram's predictive accuracy.

### 2.5 Gene Set Enrichment Analysis

The HNBC samples were classified into high-risk and low-risk groups based on the median risk scores from the prognostic model. Gene Set Enrichment Analysis (GSEA) was conducted using the c2.cp.kegg.v7.4.symbols.gmt gene set and the "clusterProfiler" package in R to investigate potential molecular mechanisms driving HNBC progression.

### 2.6 Immune infiltration and drug sensitivity analysis

The ssGSEA package in R was used to profile the abundance of various immune cells across all HNBC samples. Differences in immune cell infiltration between the RR and RS groups were examined, and the proportion of infiltrated immune cells in the high-risk and low-risk groups was assessed using ssGSEA. Correlation thresholds were set at p < 0.01 for risk scores and differential immune cells, and p < 0.05 for prognostic genes and differential immune cells. The oncoPredict package was used to evaluate drug sensitivity in different risk groups.

### 2.7 Statistical analysis

Data analysis and visualization were conducted using R software (version 4.2.1). The Wilcoxon test was used to identify differences between groups, and the Spearman correlation coefficient was applied to assess relationships between variables.

## 3 Results

### 3.1 Acquisition and correlation analysis of drug-resistant associated ARGs

**Fig. 1:**
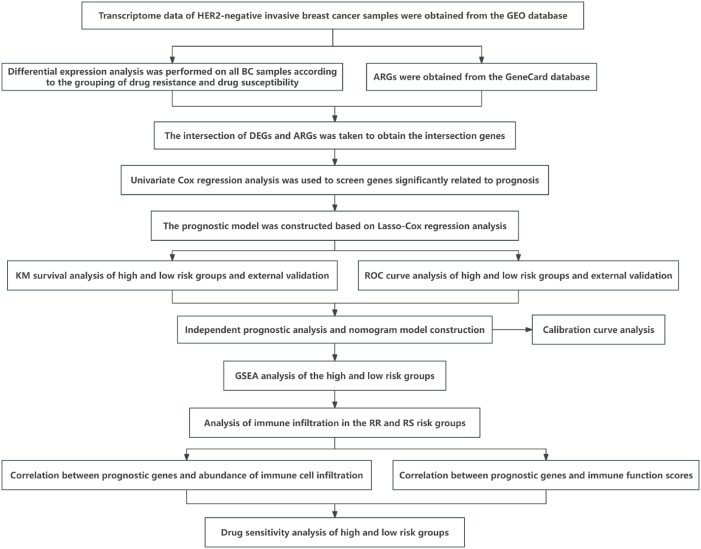
Technical roadmap.

**Fig. 2:**
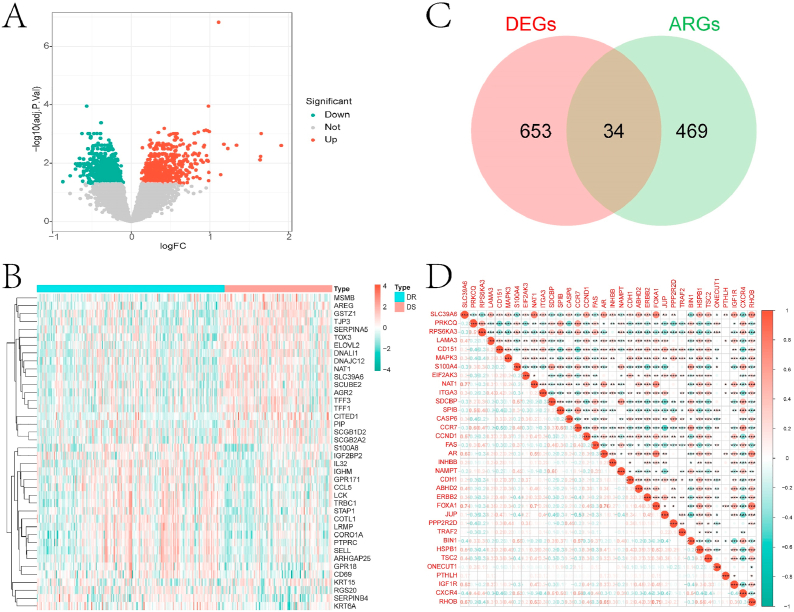
Differential expression analysis between RR and RS groups of Fig. 2 HNBC samples. A is the DEGs obtained from differential expression analysis between the two groups. B is the heat map of differential expression of Top 20 DEGs in the two groups. C is the Venn diagram of the intersection of DEGs with the previously collected ARGs. D is the correlation heatmap between intersection genes.

Fig. 1 illustrates the overall flowchart of this study. Initially, we collected and organized the transcriptome data of the GSE25055 dataset, which includes samples of both chemotherapy-sensitive and insensitive HNBC. Differential expression analysis was conducted using the limma package, resulting in a total of 687 DEGs (Fig. 2A–B). The intersection of DEGs with previously collected ARGs yielded 34 overlapping genes (Fig. 2C). These intersecting genes were defined as drug-resistant associated ARGs for subsequent analysis. Correlation analysis indicated a significant correlation among the majority of these genes (Fig. 2D).

### 3.2 Process and results of building a prognostic model based on drug-resistant-related ARGs

**Fig. 3:**
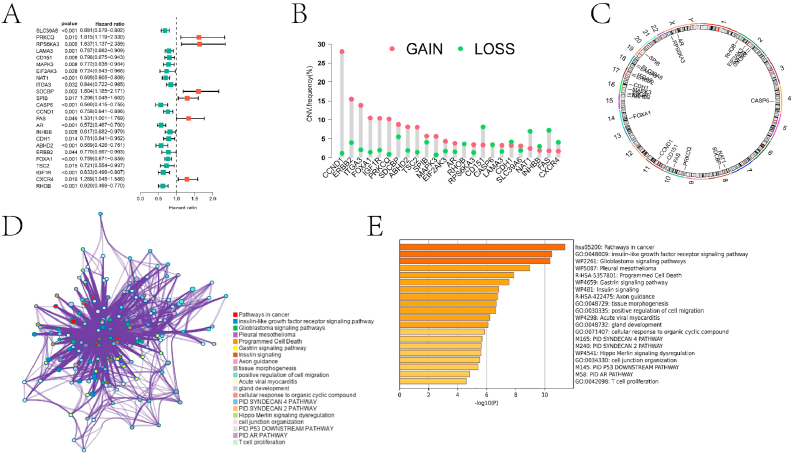
Results of comprehensive analysis of ARGs related to drug resistance. A is the forest plot obtained during the screening of prognostic genes based on univariate COX analysis. B is the result of CNV analysis of prognostic genes. C is a schematic representation of the chromosomal location of the prognostic genes. D and E are the pathway network and bar graphs obtained from metascape enrichment analysis of prognostic genes, respectively.

By performing single-factor Cox analysis on 34 drug-resistance-related ARGs, 24 ARGs (including SLC39A6, PRKCQ, RPS6KA3, LAMA3, CD151, MAPK3, EIF2AK3, NAT1, ITGA3, SDCBP, SPIB, CASP6, CCND1, FAS, AR, INHBB, CDH1, ABHD2, ERBB2, FOXA1, TSC2, IGF1R, CXCR4, and RHOB) were identified as prognostic genes (P < 0.05) (Fig. 3A). Most of these genes exhibited copy numbers higher than the normal level, which could lead to gene overexpression (Fig. 3B). These genes are primarily located on chromosomes 2, 15, 16, and 17 (Fig. 3C). Metascape enrichment analysis identified multiple pathways associated with the progression of HNBC (Fig. 3D–E).

**Fig. 4:**
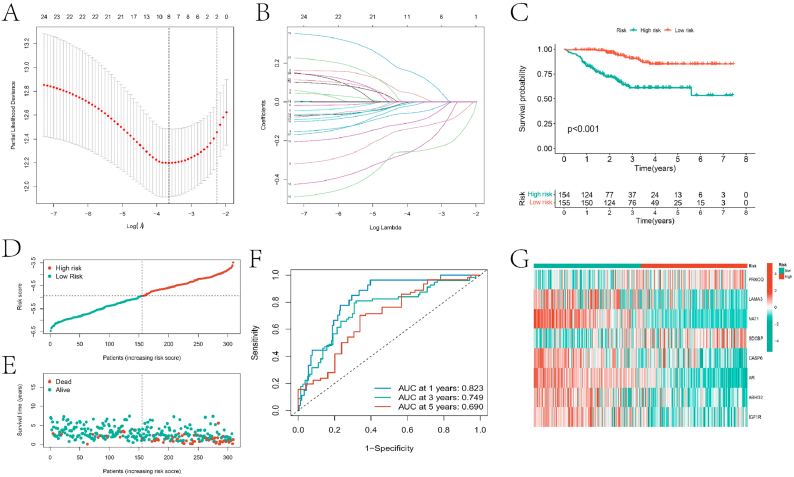
prognostic model construction results. A and B are the results of LASSO-Cox analysis of prognostic genes. C is the result of KM survival analysis of BC samples after dividing them into high and low risk groups based on the prognostic model. D and E are the score and survival status distribution plots for the two groups, respectively. F is the ROC curve for predicting 1 -, 3 -, and 5-year survival rates of BC samples based on the prognostic model, and G is the heatmap of expression of the eight genes used to construct the prognostic model in the two groups.

Additionally, LASSO-Cox regression analysis was conducted on these 24 prognostic genes to develop a prognostic model for HNBC (Fig. 4A–B). Based on the risk scores provided by the model, all HNBC samples were classified into high-risk and low-risk groups, with a significant difference in survival rates between the two groups (p < 0.001) (Fig. 4C). The high-risk group samples showed poorer survival outcomes (Fig. 4D–E). Furthermore, the AUC values for predicting 1-year, 3-year, and 5-year survival rates based on the prognostic model were 0.823, 0.749, and 0.690, respectively, indicating high predictive accuracy (Fig. 4F). Significant differences in the expression levels of eight genes (PRKCQ, LAMA3, NAT1, SDCBP, CASP6, AR, ABHD2, and IGF1R) were observed between the high and low-risk groups (Fig. 4G).

**Fig. 5:**
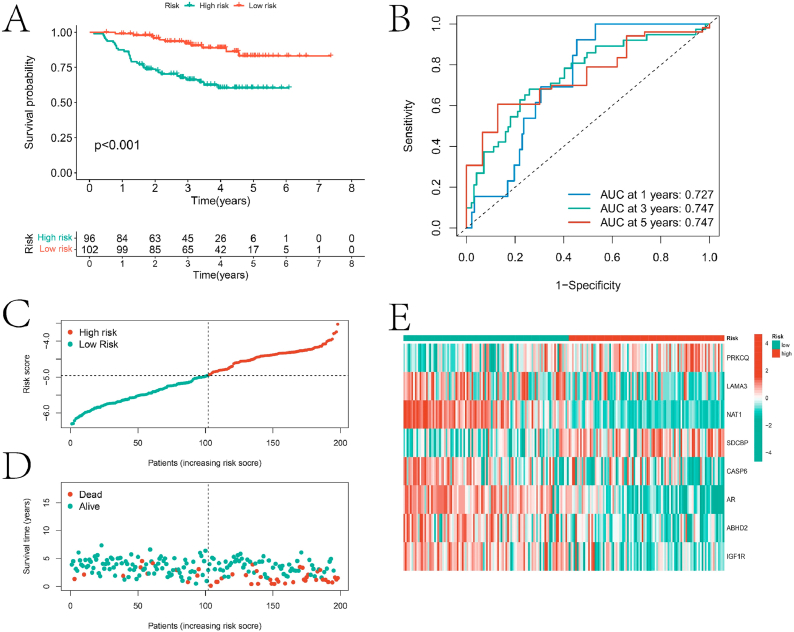
External validation results of the prognostic model. A is the result of KM survival analysis of the two groups in the validation set based on the prognostic model. B is the ROC curve for predicting 1, 3, and 5 year survival rates of the test set BC samples based on the prognostic model. C and D are the distribution plots of scores and survival status of the two groups of samples in the test set, respectively. E is the expression heatmap of the eight genes in the two groups of the test set.

To further validate the robustness of the prognostic model, significant differences in survival time between high-risk and low-risk groups were also observed in the test set samples (p < 0.001) (Fig. 5A). The model's AUC values for predicting 1-year, 3-year, and 5-year survival rates in the test set were 0.727, 0.747, and 0.747, respectively (Fig. 5B). Moreover, the samples in the test set exhibited similar trends in score distribution, survival status, and expression levels compared to the training set (Fig. 5C–E).

### 3.3 Independent prognostic analysis and construction of nomogram models

**Fig. 6:**
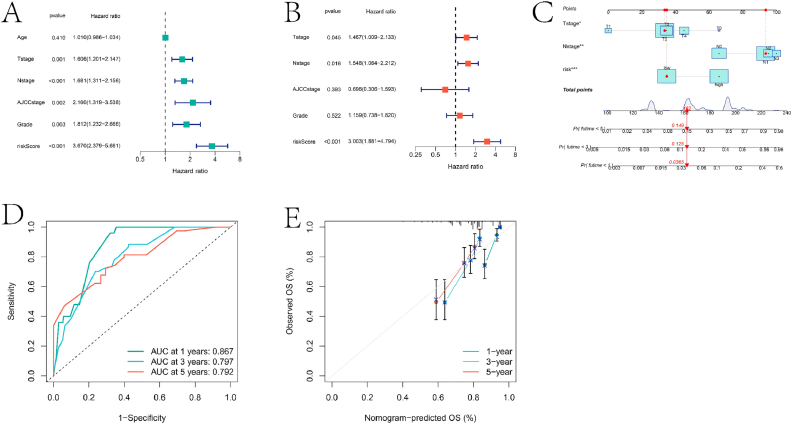
Independent prognostic analysis and nomogram model construction results. A and B are forest plots obtained from univariate and multivariate Cox regression analyses of clinical characteristics and risk scores, respectively. C is the nomogram model constructed using clinical characteristics and risk scores. D is the ROC curve of the nomogram model predicting 1 -, 3 -, and 5-year survival rates for the HNBC sample. E is the calibration curve of the nomogram model.

In order to enhance the practicality of the prognostic model, we integrated the risk score with clinical features (age, gender, and stage) to develop a nomogram model for predicting outcomes in HNBC patients. Specifically, we sequentially utilized univariate and multivariate Cox regression models to consider T stage, N stage, The International American Joint Committee on Cancer (AJCC) stage, Grade, and the risk score as independent prognostic factors (Fig. 6A–B). Subsequently, a nomogram model was constructed based on T stage, N stage, and the risk score (Fig. 6C). The nomogram model demonstrated higher predictive accuracy for 1-year, 3-year, and 5-year survival rates in HNBC samples, with AUCs of 0.867, 0.797, and 0.792, respectively (Fig. 6D), compared to previously established prognostic models. The calibration curve of the nomogram model indicates its good performance in predicting patient prognosis (Fig. 6E).

### 3.4 Results of enrichment, immune, and drug sensitivity analysis

**Fig. 7:**
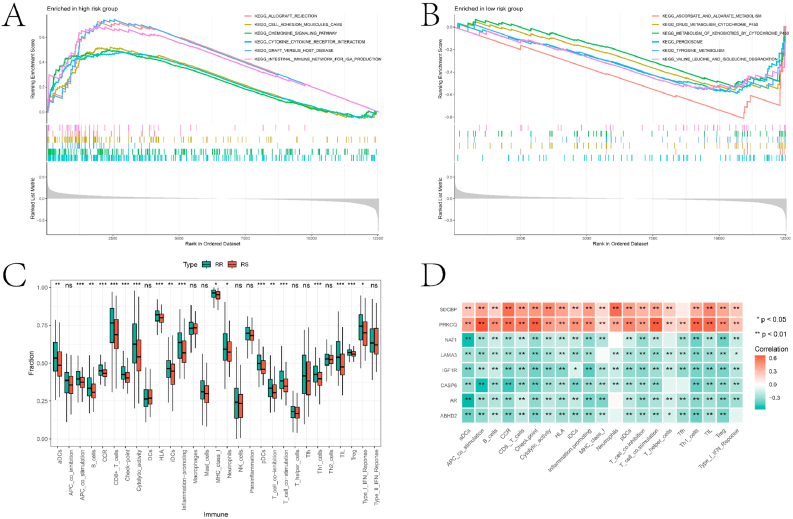
GSEA analysis and immune landscape for high and low risk groups. A and B are the results of the GSEA analysis of the high and low risk groups, respectively. C is the boxplot of the difference in immune cell infiltration abundance and immune function scores between the two groups based on the ssGSEA package. D is the heat map of the correlation between prognostic genes and the abundance of immune cell infiltration/immune function score.

**Fig. 8:**
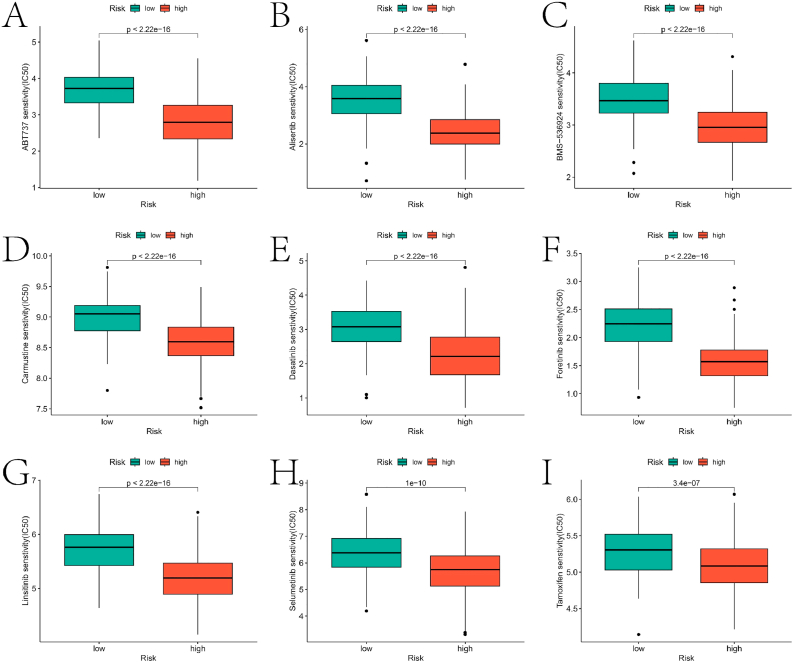
Results of drug sensitivity analysis between different risk groups. A-I is the IC50 value between the high and low risk groups of drugs that have been confirmed to be used in the treatment of BC.

To explore the biological functions and pathways linked to ARGs in constructing the prognostic model, we performed GSEA on the two risk categories. Fig. 7A–B highlight the top 6 pathways associated with the high-risk and low-risk groups, respectively. The connection between these pathways and HNBC will be further discussed in the subsequent section. Additionally, differences in immune cell infiltration levels and immune function scores between the RR and RS groups were analyzed using the single-sample Gene Set Enrichment Analysis (ssGSEA) package (Fig. 7C). There were significant differences in the abundance/functional scores of various immune cells/functions between the two groups, with the RR group exhibiting higher infiltration abundance/scores. Most prognostic genes used in constructing the prognostic model showed significant correlation with immune cells/immune functions (Fig. 7D). Lastly, sensitivity analysis of various drugs between the two groups revealed the critical roles of several drugs (ABT737, Alisertib, BMS-536924, Dasatinib, Foretinib, Selumetinib, and Tamoxifen) in the treatment of HNBC (Fig. 8A–I). Box plots illustrating the sensitivity of other drugs with significant differences between the two groups are provided in the supplementary material's "drug" folder.

## 4 Discussion

HER2-negative breast cancer (HNBC), compared to other breast cancer subtypes, presents a particularly high risk of distant metastasis and recurrence, posing significant challenges in its clinical management. Current treatment approaches primarily rely on taxane- and anthracycline-based chemotherapies, which, while effective in many cases, are often hindered by the emergence of chemotherapy resistance. Anoikis, a specialized form of programmed cell death, plays a vital role in maintaining tissue integrity by eliminating detached or damaged cells and preventing abnormal cell proliferation. However, cancer cells frequently develop resistance to anoikis, facilitated by alterations in the extracellular matrix and disruptions in cell-cell interactions. This resistance enables cancer cells to evade apoptosis, survive in anchorage-independent conditions, and promote tumor growth, invasion, and metastasis. This study explores the role of drug-resistant-related ARGs in HNBC.

Firstly, 24 relevant drug-resistant-related ARGs were identified through univariate Cox regression analysis. These genes showed significant correlation with the prognosis of HNBC. Metascape enrichment analysis of these genes identified multiple pathways related to HNBC. The analysis directly revealed cancer pathways. In addition, scholars found associations between Nadia de Gruil (IGF1) and insulin receptor single nucleotide variations with the response of HNBC patients to fasting-mimicking diet therapy [11]. Wong et al. found overexpression of gastric inhibitory polypeptide receptor in HNBC samples [12]. Consolidation therapy with activated T cells armed with anti-CD3 × anti-HER2 bispecific antibodies may improve the treatment effectiveness of HNBC [13].

PRKCQ is a protein kinase that plays a crucial role in T-cell signaling and is closely associated with cell survival, proliferation, and inflammatory responses. Quanxiao Xu et al. demonstrated that saffron can inhibit NF-κB-mediated inflammation and proliferation in breast cancer (BC) cells by downregulating PRKCQ expression [14]. LAMA3, a component of the basement membrane, is involved in cell-cell and cell-matrix adhesion, influencing cell migration and tissue architecture. High LAMA3 expression is strongly associated with cancer invasion and metastasis, particularly in pancreatic cancer [15]. NAT1, an enzyme involved in drug metabolism, regulates the metabolism of aromatic amine compounds and may influence the tumor microenvironment through metabolic state regulation [16]v. Katherine L. Pogue-Geile et al. developed a gene expression-based trastuzumab benefit prediction model (including NAT1), indicating that HER2-negative breast cancer (HNBC) falls within the moderately beneficial group [17]. SDCBP, a cytoskeleton-associated protein, participates in signal transduction, cell migration, and intercellular communication. Its overexpression is linked to enhanced invasive and metastatic capabilities of tumor cells [18]. Tianjie Pu et al. found that inhibiting miR-135b-5p promotes early BC metastasis by regulating the downstream target SDCBP [19]. CASP6, a caspase, is involved in apoptosis and inflammatory responses. In BRCA, CASP6 inactivation or overactivation may be associated with tumor suppression or cell survival, respectively [20]. AR, the androgen receptor, regulates androgen-dependent gene expression. In BRCA, AR expression levels are correlated with patient prognosis [21]. ABHD2, a lipid metabolism-associated enzyme, modulates signaling lipids on the cell membrane. It may influence signal transduction by regulating membrane lipid composition, thereby playing a role in BC cell proliferation and migration [22]. IGF1R, a tyrosine kinase receptor, mediates growth factor signaling, affecting cell growth, differentiation, and survival. In BRCA, high IGF1R expression is associated with more aggressive subtypes [23].

The prognostic model demonstrates robust accuracy in predicting outcomes for both training and testing datasets. Utilizing the model's risk scores, HNBC samples were categorized into high and low-risk groups. Key pathways identified through GSEA in both categories are significantly linked to HNBC progression. Notably, L1 cell adhesion molecule (L1CAM) is prominently expressed in HNBC, serving as a potential marker for recurrence and aggressive disease behavior [24]. Research by Ga-Eon Kim et al. highlights the chemokine signaling pathway as crucial in the transition from cancer to lymph node metastasis in HNBC patients [25]. Additionally, human epidermal growth factor receptor 2, through mechanisms like gene amplification, mutation, or overexpression, contributes to HNBC carcinogenesis [26]. Interestingly, the prognostic model based on drug resistance-related genes (ARGs) that we constructed revealed a significant difference in survival rates between the high-risk and low-risk groups during the first five years, while after 6–8 years, the survival rates of the two groups tended to converge. We hypothesize that, in addition to ARGs, multiple factors may have played a role in this process. First, the immune environment may have played a critical role in survival during advanced stages. Our analysis showed significant differences in immune cell infiltration levels and immune function scores between the high-risk and low-risk groups, with the RR group exhibiting higher immune cell infiltration, which might contribute to improved survival. Second, GSEA analysis identified multiple pathways associated with tumor progression, which could explain the early survival differences. Over time, the impact of these pathways may diminish, leading to the convergence of survival rates. Additionally, genetic and epigenetic factors, as well as changes in the tumor microenvironment, may also influence survival at later stages. In particular, tumor immune evasion and dormancy mechanisms may affect long-term survival, further altering the survival differences between the two groups. Finally, clinical treatment interventions, individual patient differences, and their adherence to treatment may also impact survival outcomes at later stages. Taken together, while ARGs play a crucial role in early survival differences, other molecular, immune, and clinical factors also have a significant influence on long-term survival and merit further investigation in future studies.

A new nomograph model was developed with superior predictive accuracy compared to the initial prognostic model, incorporating both risk scores and clinical factors. We further investigated immune cell infiltration and immune function differences between the RR and RS groups. Research by Rubén Osuna-Gómez et al. revealed that HNBC patients post-neoadjuvant chemotherapy engage in cytotoxic and pro-inflammatory responses involving CD8^+^ HLA-DR + T cells [27]. Furthermore, early-stage HNBC patients receiving neoadjuvant chemotherapy combined with dendritic cell vaccines achieved pathological complete remission [28]. Eftilagmod alpha (efti), a soluble LAG-3 protein and MHC class II agonist, has shown safety and efficacy in combination with paclitaxel for HNBC treatment [29].

Lastly, drug sensitivity analysis across high and low-risk groups identified several crucial drugs for HNBC management. Clinical studies indicate that oral alisertib significantly enhances progression-free survival when added to a reduced weekly dose of paclitaxe. The recommended dosage and effectiveness of dasatinib combined with zoledronic acid for bone-dominant HNBC metastasis were evaluated in phase 2 trials by Zahi Mitri et al. [30]. Conversely, Juhyeon Lee et al. found no substantial survival benefits in HNBC patients receiving chemotherapy before tamoxifen combined with GnRH agonists compared to those who did not undergo chemotherapy [31].

The limitations of this study must be acknowledged to provide a balanced view of the findings. The study utilized a relatively small dataset, which may limit the generalizability of the results, and there was no external validation cohort to confirm the prognostic model's effectiveness. Additionally, the data were obtained from a single center, introducing potential bias related to specific patient demographics or treatment protocols. While bioinformatics analysis offered valuable insights into ARGs and their role in chemotherapy resistance, functional experiments were limited, necessitating further laboratory-based studies to validate the computational predictions. The analysis of the immune microenvironment and drug sensitivity was based on computational predictions, which require experimental validation to confirm their implications for therapy. Finally, while the potential of ARGs as biomarkers for HNBC was suggested, comprehensive clinical trials are needed to establish ARGs as reliable biomarkers for personalized treatment strategies. In addition, one of the key limitations of this study is the small and specific dataset (GSE25055) used for the analysis. Although efforts were made to explore larger, publicly available datasets such as those from TCGA and GEO, we were unable to identify a suitable, large-scale dataset that specifically includes chemotherapy-resistant HNBC samples with the necessary annotations for ARGs. The lack of such a dataset further highlights the need for more comprehensive public repositories with detailed molecular and clinical data on chemotherapy-resistant breast cancer subtypes. Future studies should aim to incorporate data from multiple centers and larger cohorts to validate and expand upon the findings of this study. This would help overcome the limitations imposed by small sample sizes and improve the robustness and generalizability of the prognostic model developed here.

## 5 Conclusion

The manuscript constructs and validates an HNBC prognostic model based on drug-resistant ARGs. A high-precision line chart model is built using clinical factors and risk scores. Immunoinfiltration analysis identifies various immune cells and functions in the RR group and RS group. Additionally, drug sensitivity analysis reveals significant differences in IC50 values for drugs between high and low-risk groups. In conclusion, drug-resistant ARGs contribute to risk stratification in HNBC, offering insights for clinical diagnosis and drug target discovery in HNBC.

## Availability of data and materials

The data that support the findings of this study are openly available in TCGA dataset at https://portal.gdc.cancer.gov/, reference number TCGA-UCSC cohort.

## Funding statement

The authors acknowledge the support provided by Wenzhou Municipal Science and Technology Planning Project (2023Y1931)

## Declaration of competing interest

The authors declare no conflict of interest.

The authors declare that they have no known competing financial interests or personal relationships that could have appeared to influence the work reported in this paper.

## Data Availability

The data that has been used is confidential.
