# Supplementary figures and images for: Exploration of the role of drug resistance-associated anoikis-related genes in HER2-Negative breast cancer through bioinformatics analysis (part 2 of 2)
Source: Biochem Biophys Rep. 2025 Feb 21;41:101947. doi: 10.1016/j.bbrep.2025.101947 (PMC11891708; doi:10.1016/j.bbrep.2025.101947)

Risk 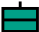 low 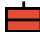 high

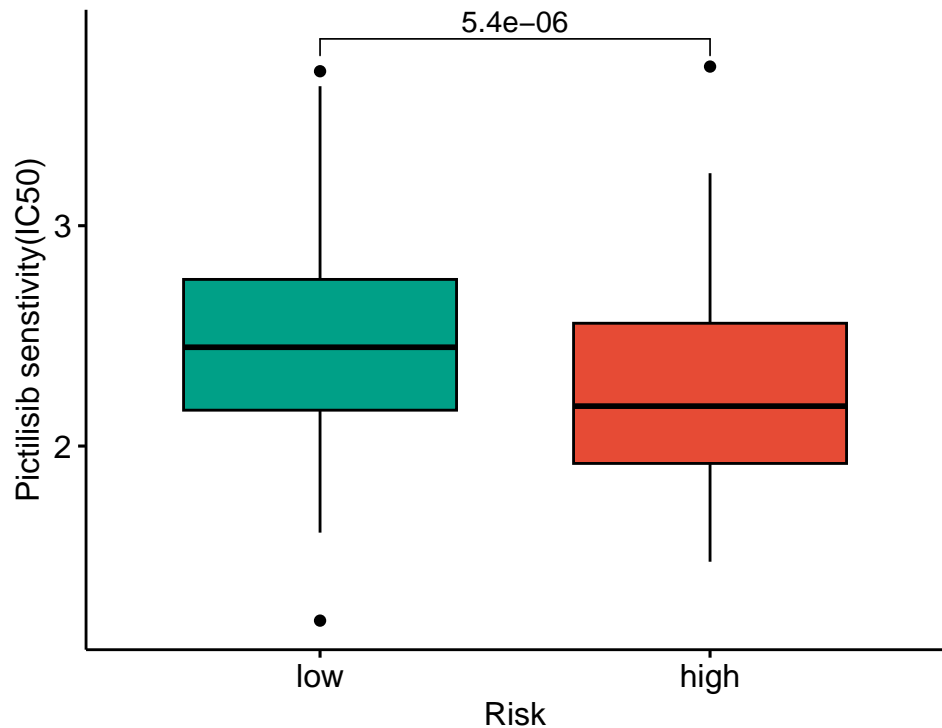

Supplement: Multimedia component 1 [file mmc1.zip › drug/drugSenstivity.Pictilisib.pdf]

Risk 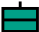 low 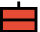 high

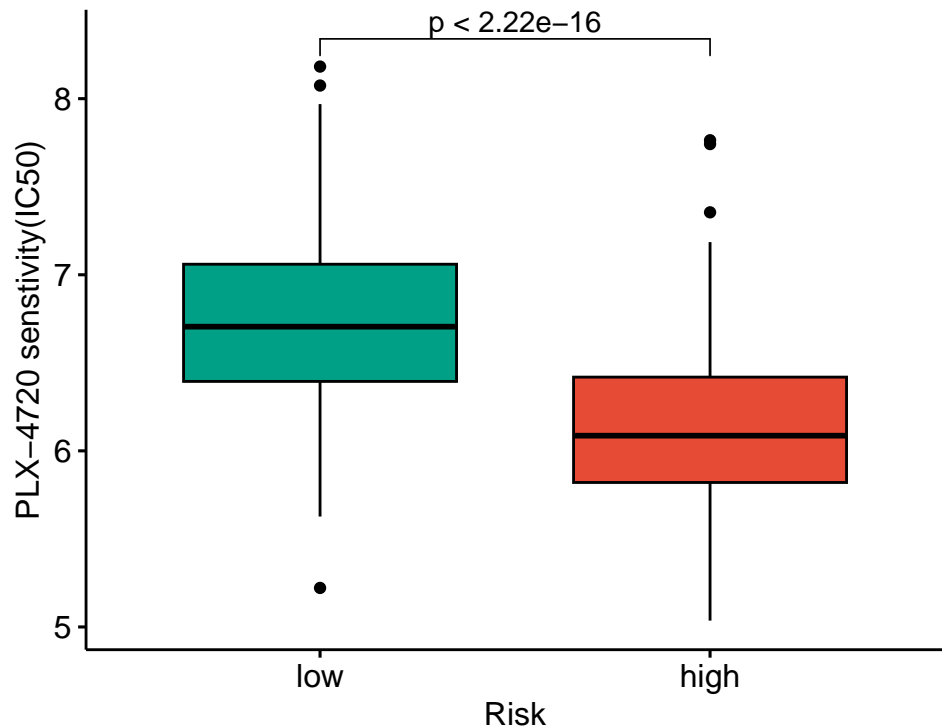

Supplement: Multimedia component 1 [file mmc1.zip › drug/drugSenstivity.PLX-4720.pdf]

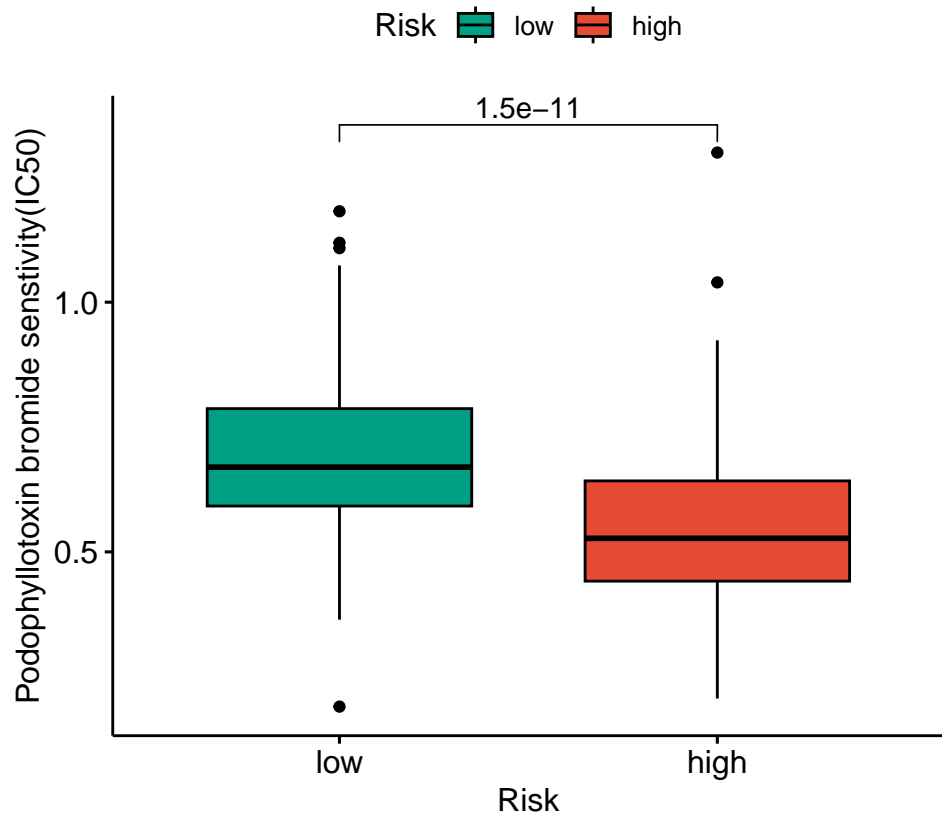

Supplement: Multimedia component 1 [file mmc1.zip › drug/drugSenstivity.Podophyllotoxin bromide.pdf]

Risk 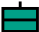 low 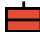 high

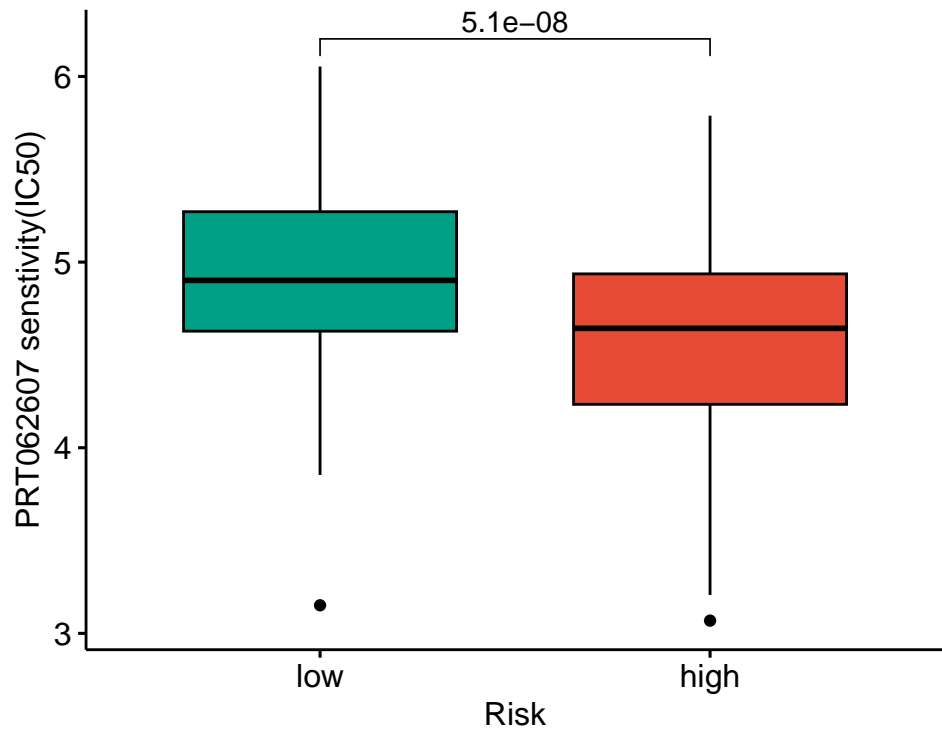

Supplement: Multimedia component 1 [file mmc1.zip › drug/drugSenstivity.PRT062607.pdf]

Risk 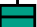 low 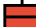 high

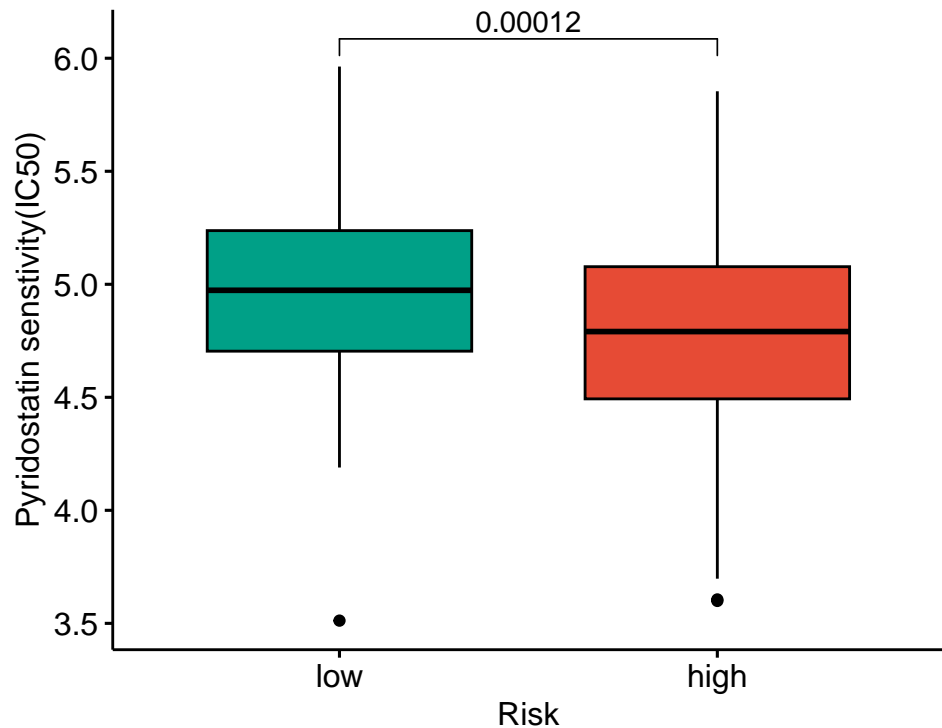

Supplement: Multimedia component 1 [file mmc1.zip › drug/drugSenstivity.Pyridostatin.pdf]

Risk 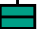 low 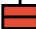 high

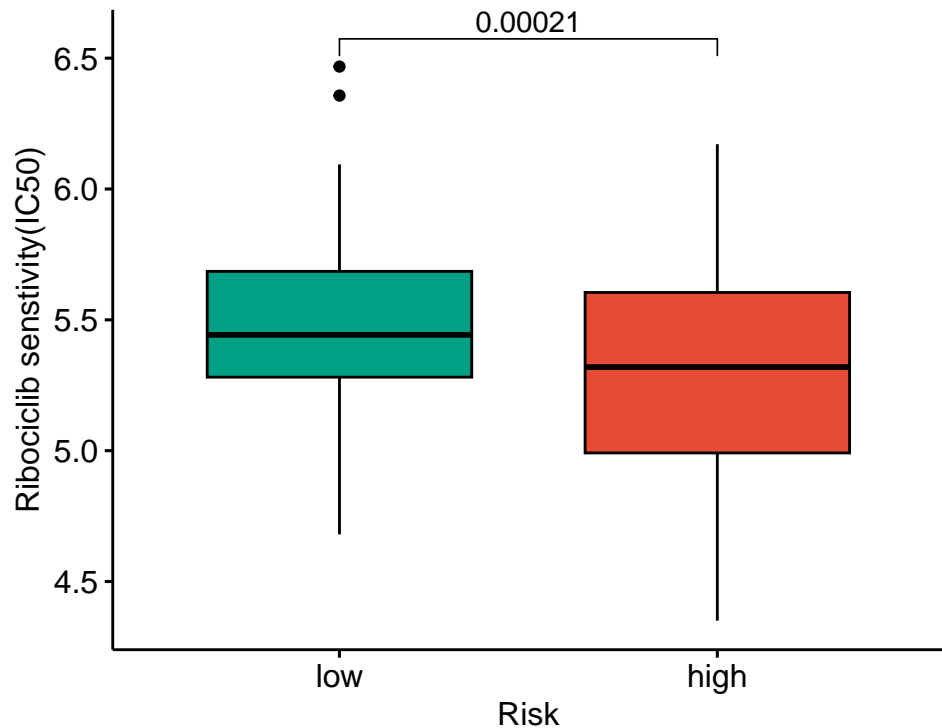

Supplement: Multimedia component 1 [file mmc1.zip › drug/drugSenstivity.Ribociclib.pdf]

Risk 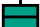 low 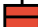 high

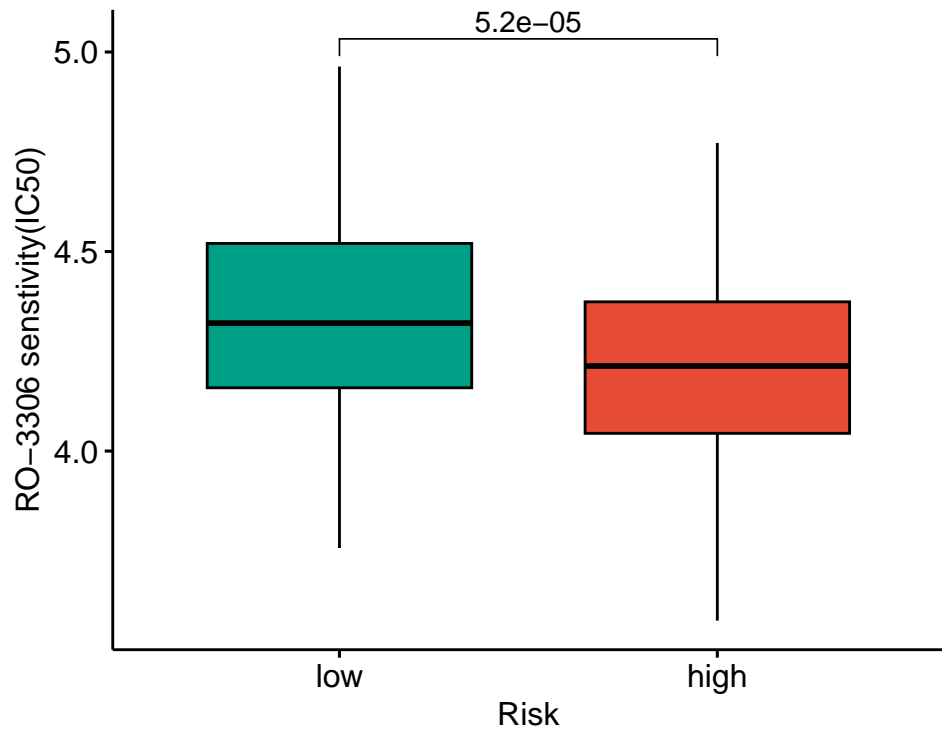

Supplement: Multimedia component 1 [file mmc1.zip › drug/drugSenstivity.RO-3306.pdf]

Risk 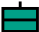 low 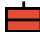 high

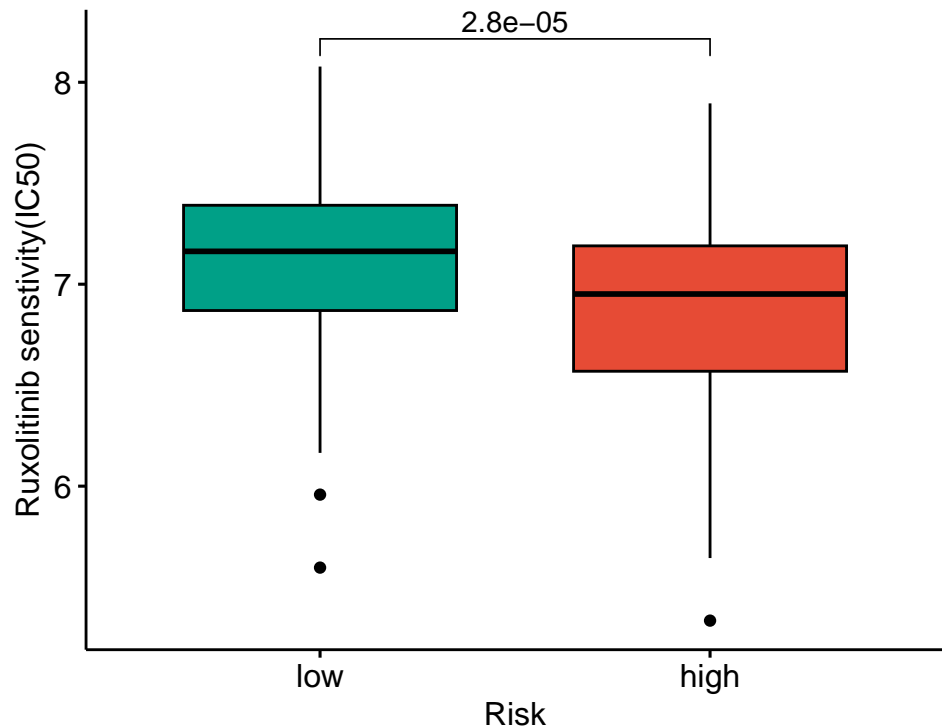

Supplement: Multimedia component 1 [file mmc1.zip › drug/drugSenstivity.Ruxolitinib.pdf]

Risk 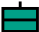 low 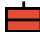 high

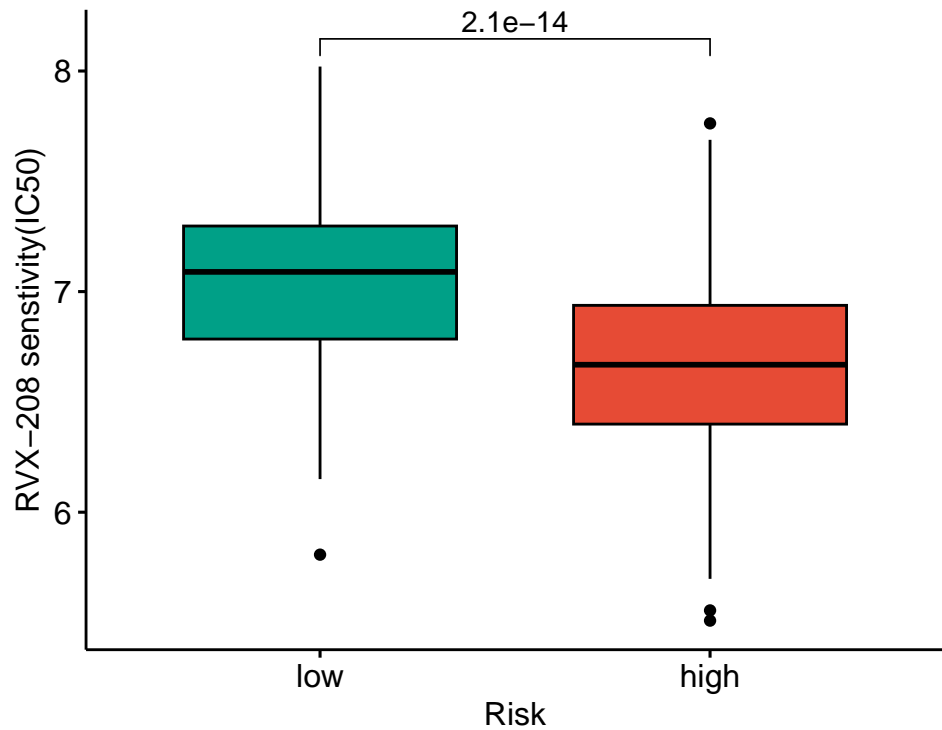

Supplement: Multimedia component 1 [file mmc1.zip › drug/drugSenstivity.RVX-208.pdf]

Risk 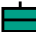 low 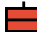 high

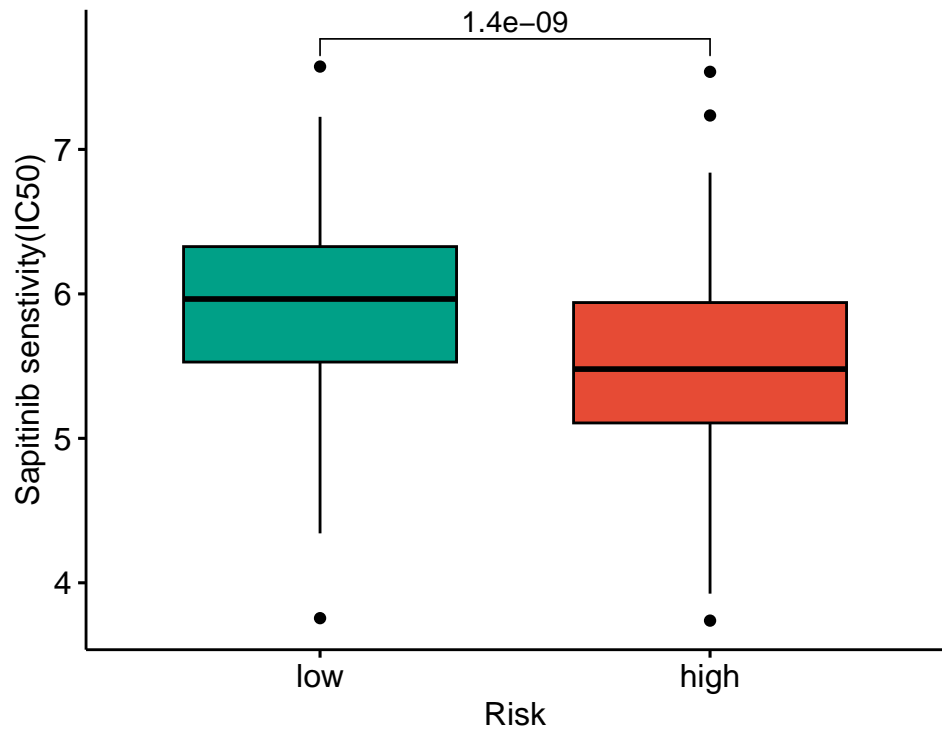

Supplement: Multimedia component 1 [file mmc1.zip › drug/drugSenstivity.Sapitinib.pdf]

Risk 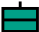 low 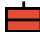 high

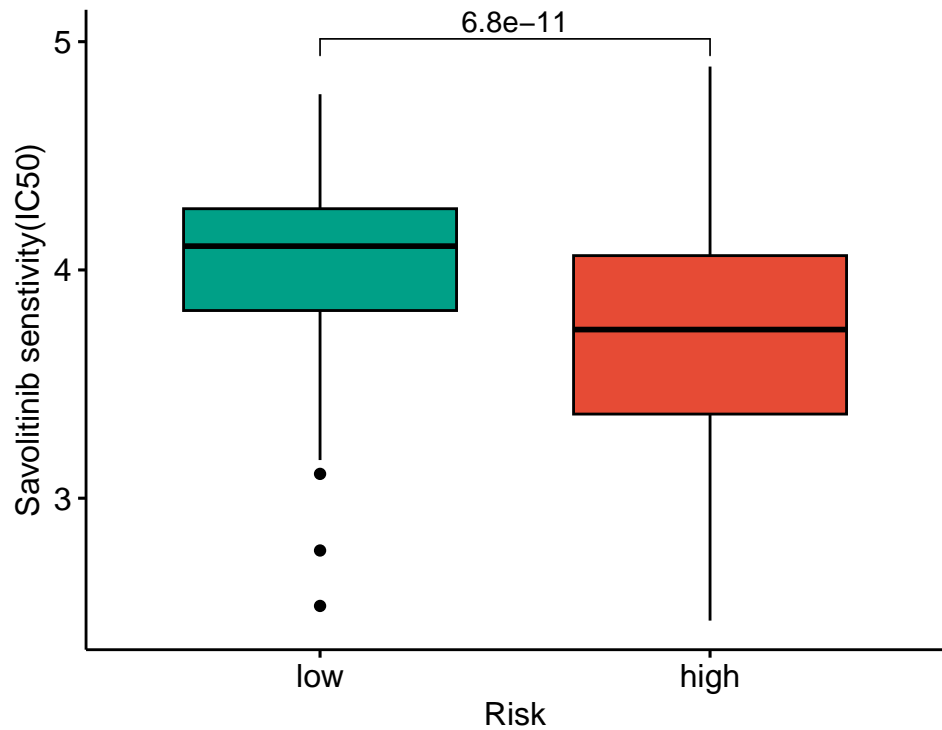

Supplement: Multimedia component 1 [file mmc1.zip › drug/drugSenstivity.Savolitinib.pdf]

Risk 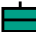 low 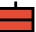 high

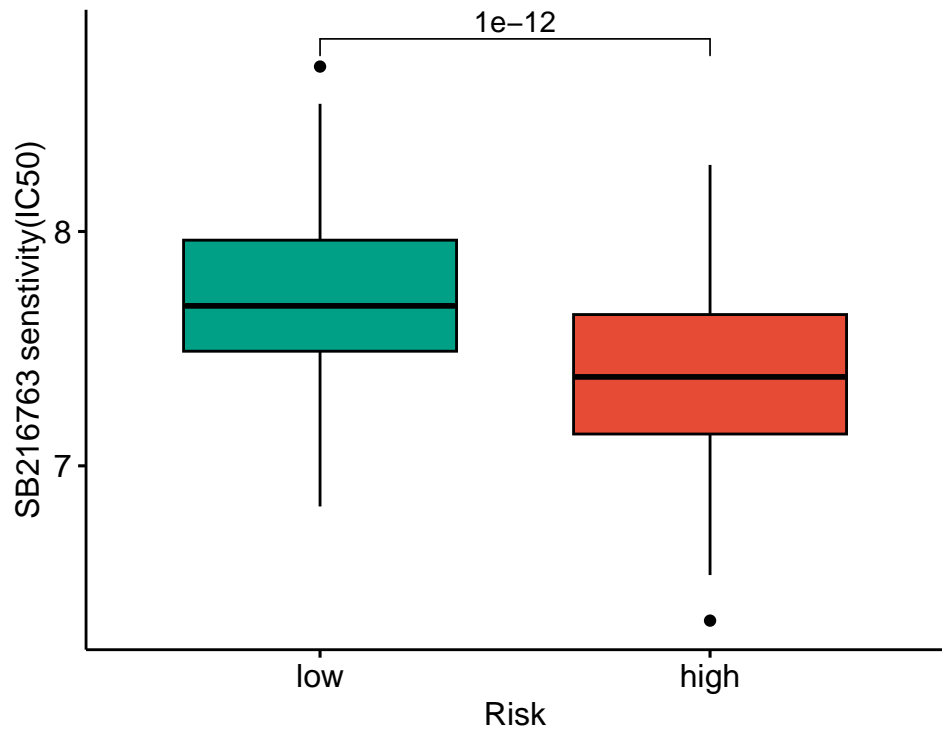

Supplement: Multimedia component 1 [file mmc1.zip › drug/drugSenstivity.SB216763.pdf]

Risk 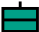 low 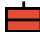 high

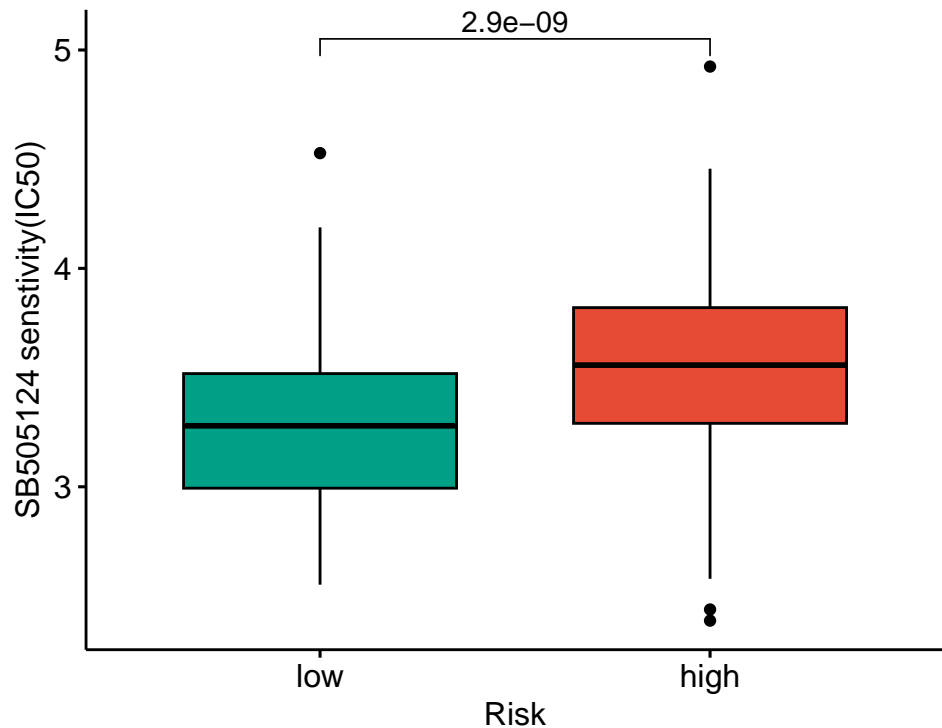

Supplement: Multimedia component 1 [file mmc1.zip › drug/drugSenstivity.SB505124.pdf]

Risk 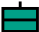 low 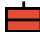 high

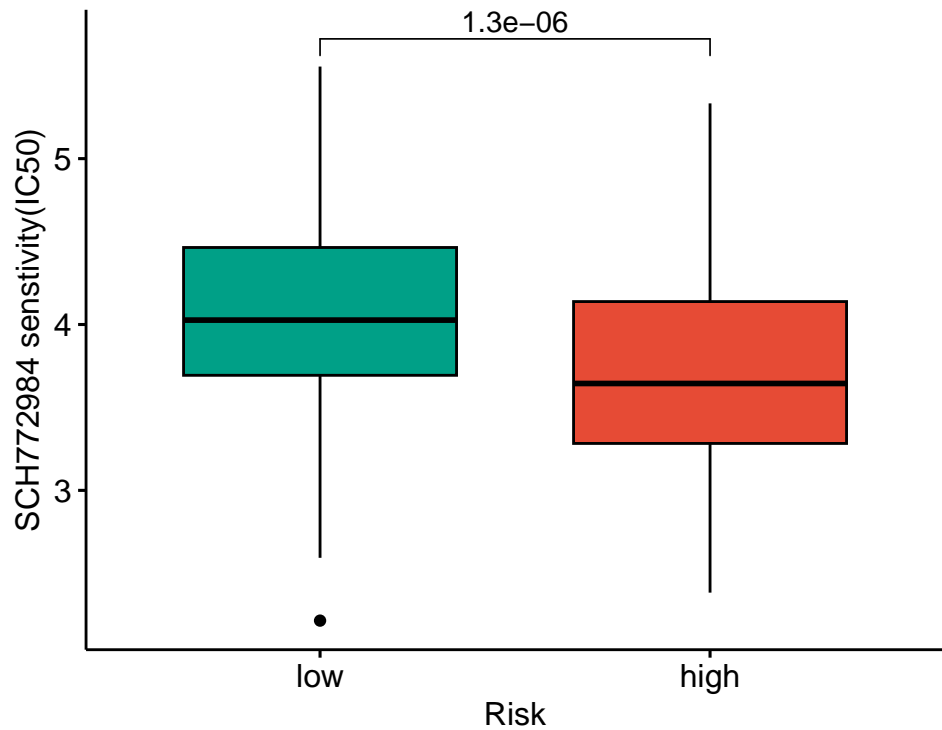

Supplement: Multimedia component 1 [file mmc1.zip › drug/drugSenstivity.SCH772984.pdf]

Risk 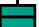 low 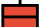 high

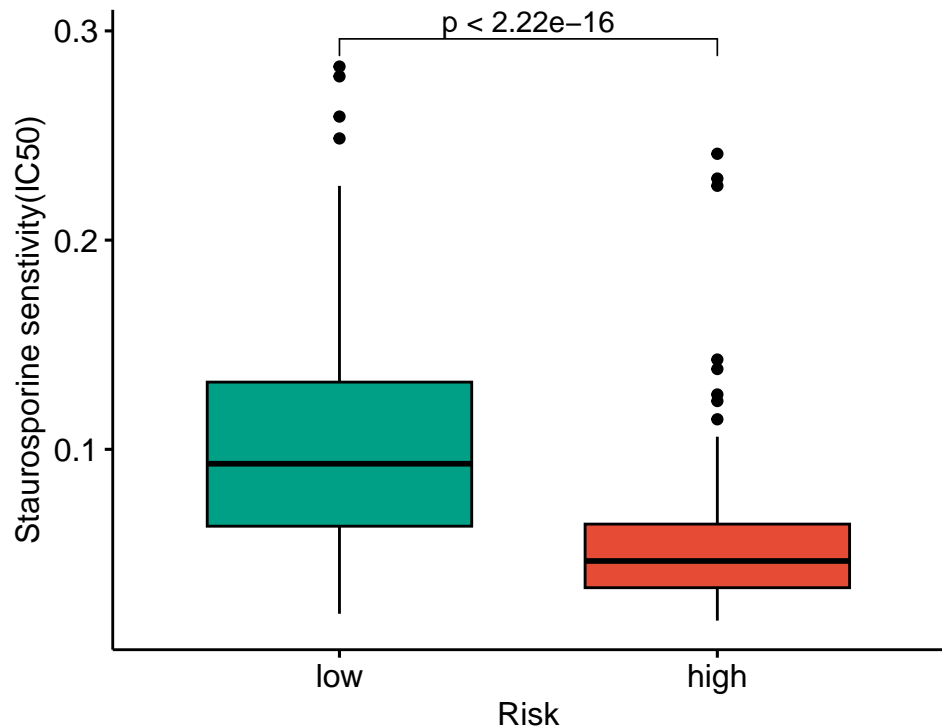

Supplement: Multimedia component 1 [file mmc1.zip › drug/drugSenstivity.Staurosporine.pdf]

Risk 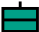 low 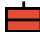 high

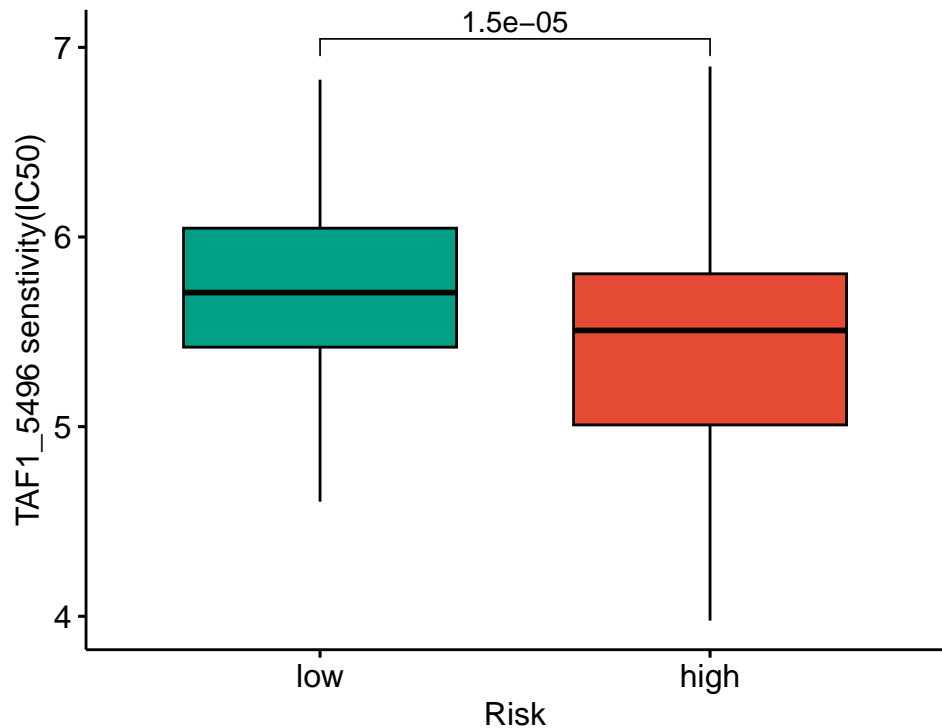

Supplement: Multimedia component 1 [file mmc1.zip › drug/drugSenstivity.TAF1_5496.pdf]

Risk 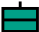 low 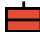 high

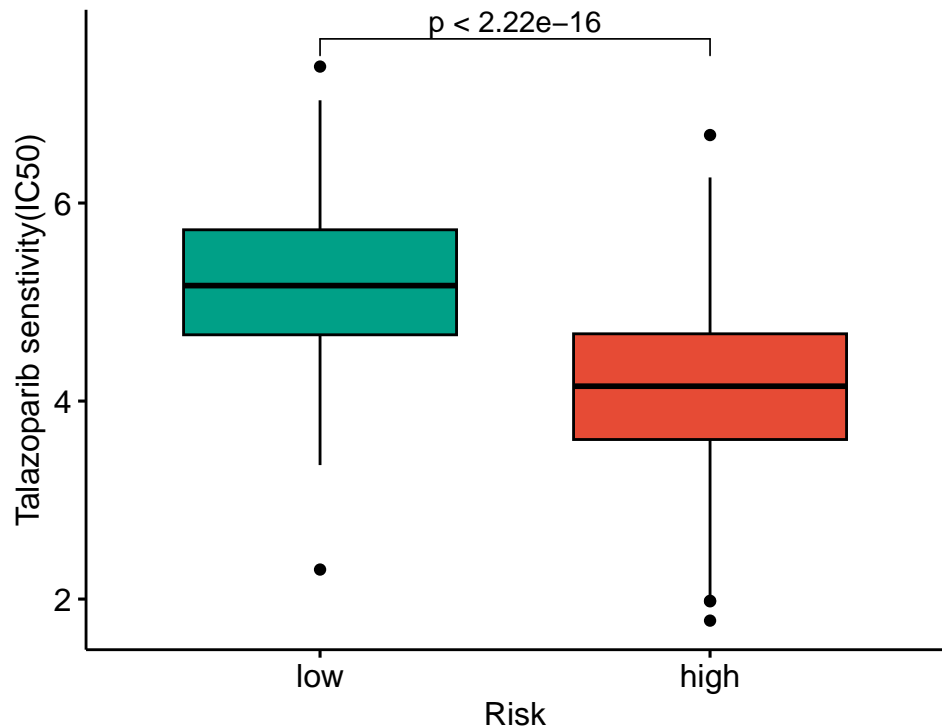

Supplement: Multimedia component 1 [file mmc1.zip › drug/drugSenstivity.Talazoparib.pdf]

Risk 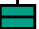 low 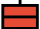 high

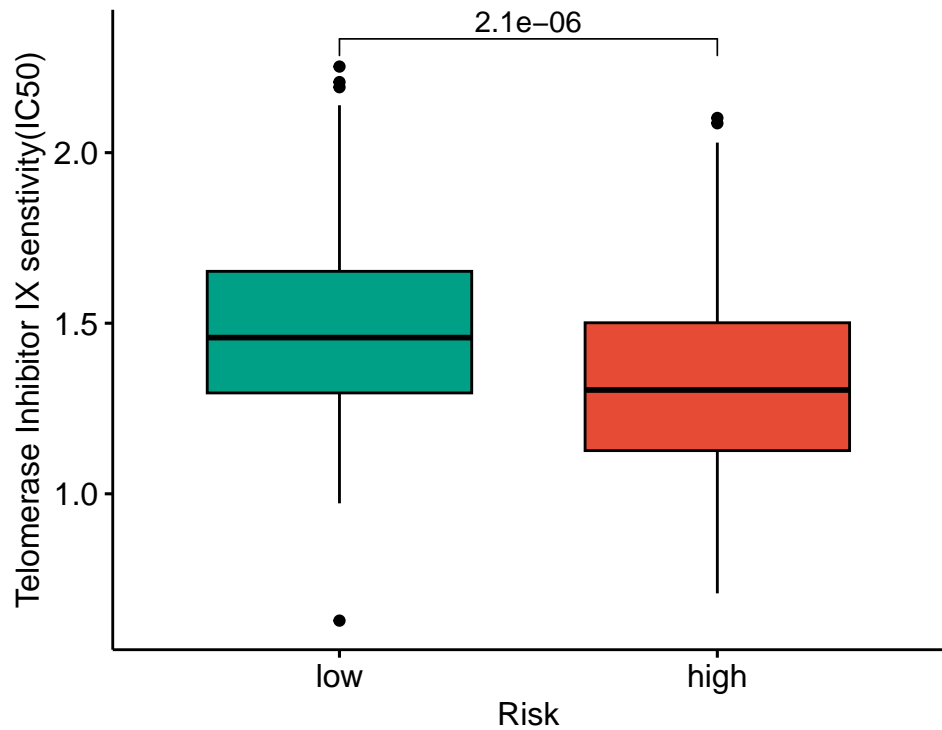

Supplement: Multimedia component 1 [file mmc1.zip › drug/drugSenstivity.Telomerase Inhibitor IX.pdf]

Risk 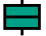 low 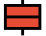 high

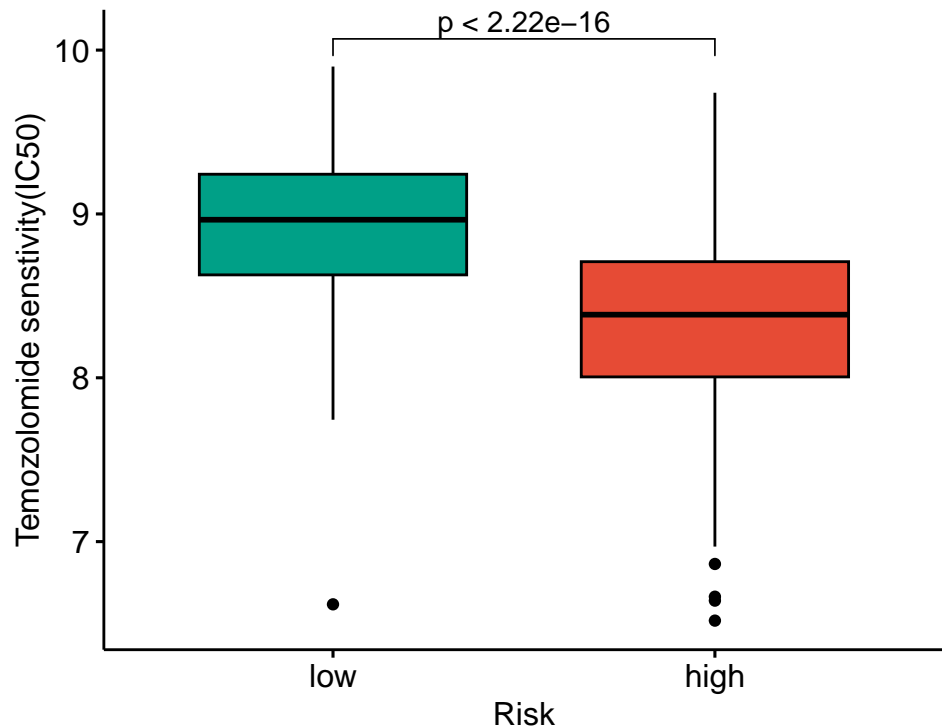

Supplement: Multimedia component 1 [file mmc1.zip › drug/drugSenstivity.Temozolomide.pdf]

Risk 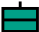 low 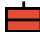 high

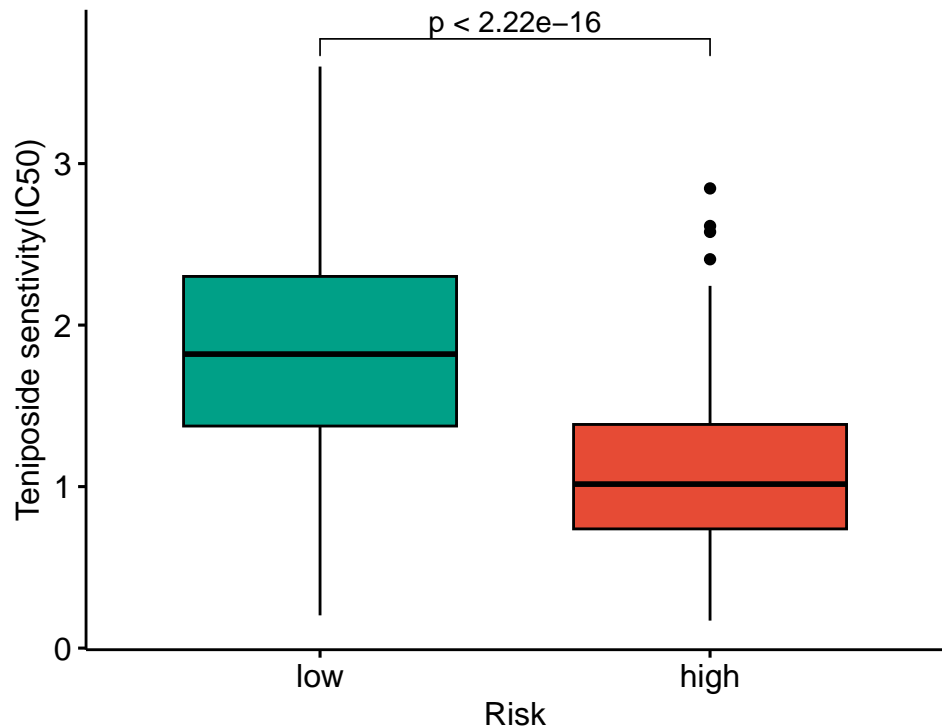

Supplement: Multimedia component 1 [file mmc1.zip › drug/drugSenstivity.Teniposide.pdf]

Risk 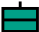 low 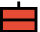 high

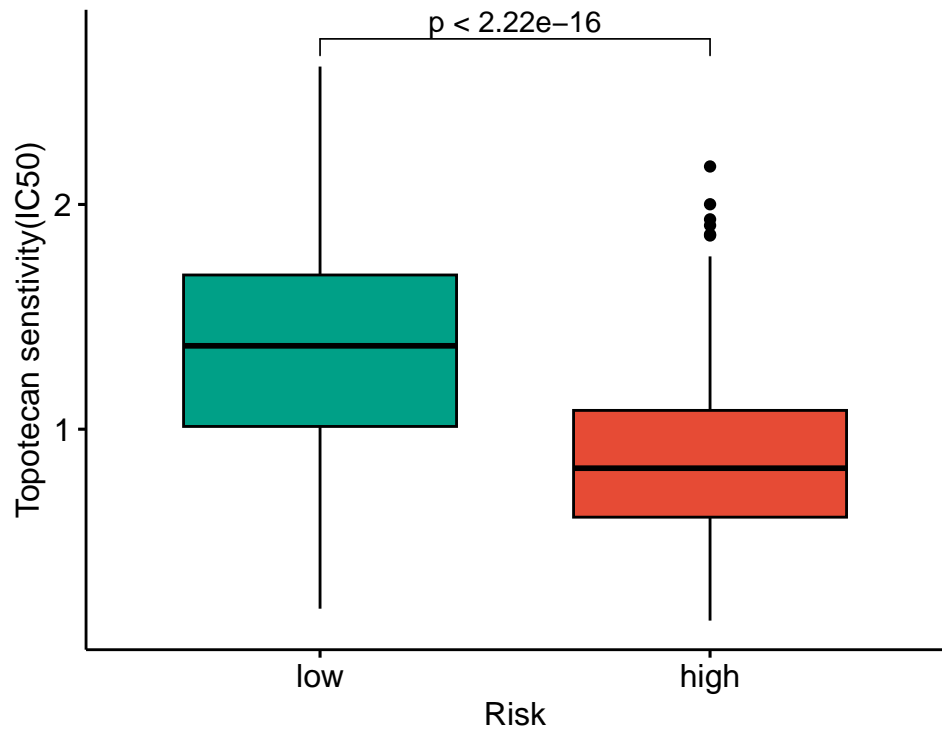

Supplement: Multimedia component 1 [file mmc1.zip › drug/drugSenstivity.Topotecan.pdf]

Risk 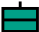 low 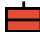 high

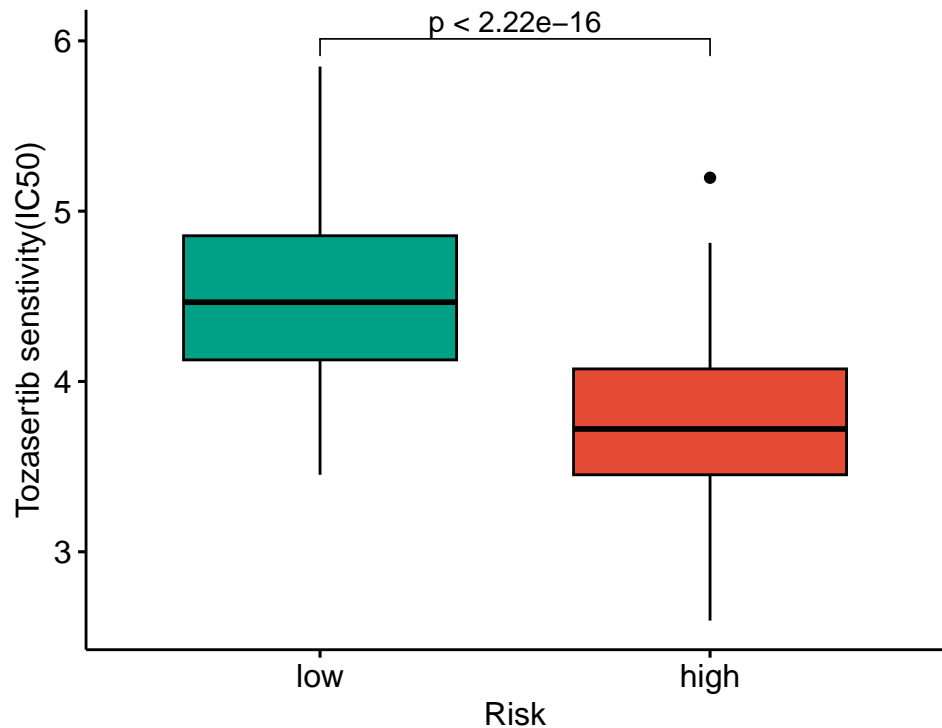

Supplement: Multimedia component 1 [file mmc1.zip › drug/drugSenstivity.Tozasertib.pdf]

Risk 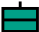 low 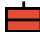 high

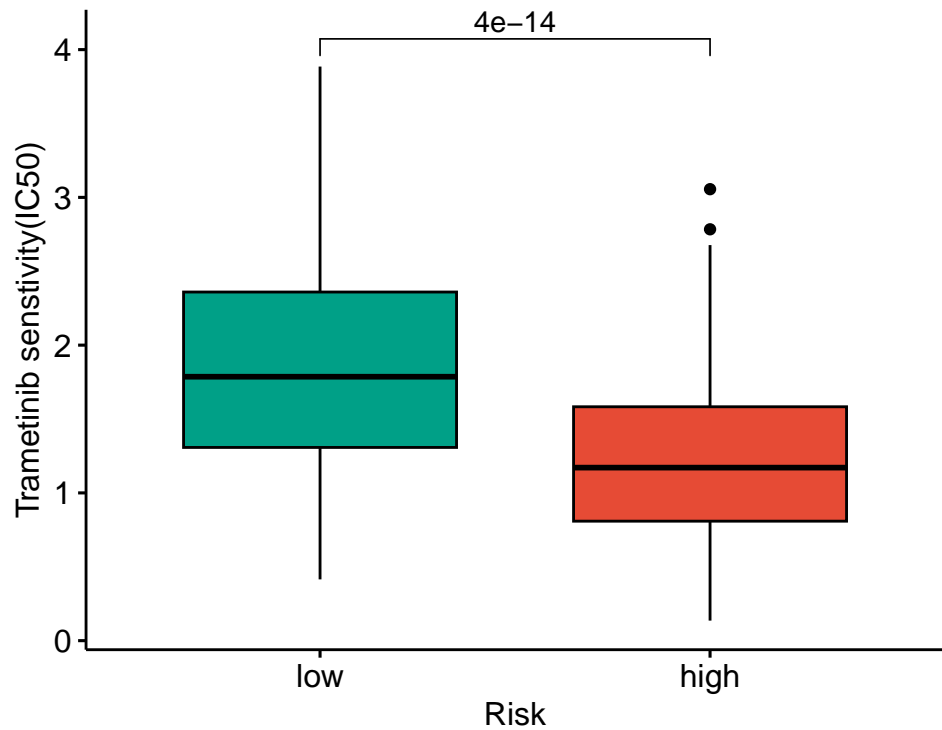

Supplement: Multimedia component 1 [file mmc1.zip › drug/drugSenstivity.Trametinib.pdf]

Risk 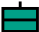 low 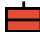 high

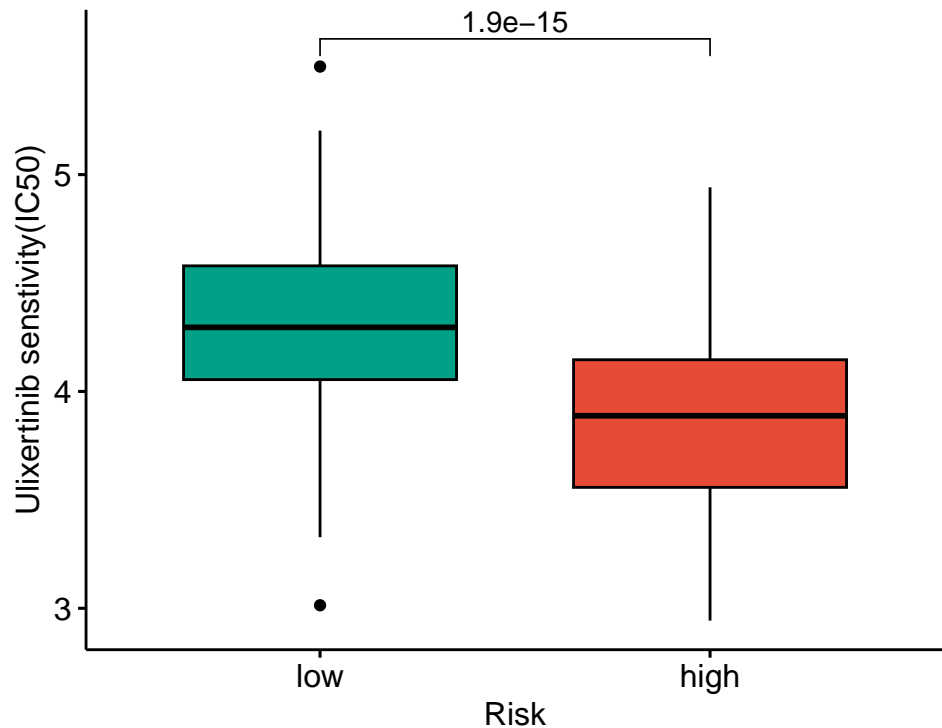

Supplement: Multimedia component 1 [file mmc1.zip › drug/drugSenstivity.Ulixertinib.pdf]

Risk 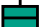 low 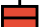 high

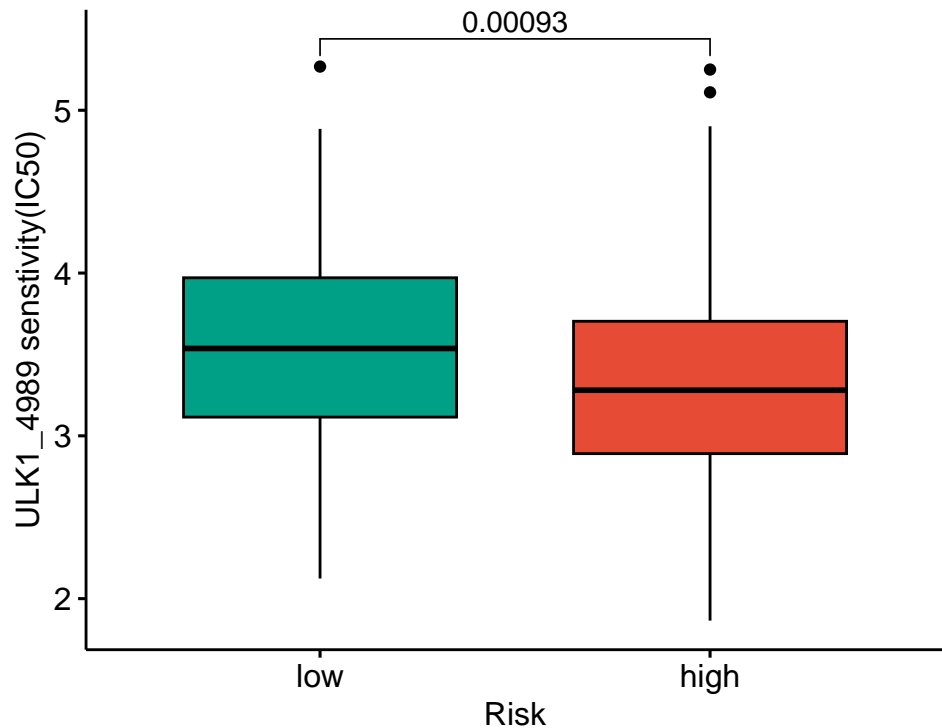

Supplement: Multimedia component 1 [file mmc1.zip › drug/drugSenstivity.ULK1_4989.pdf]

Risk 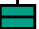 low 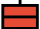 high

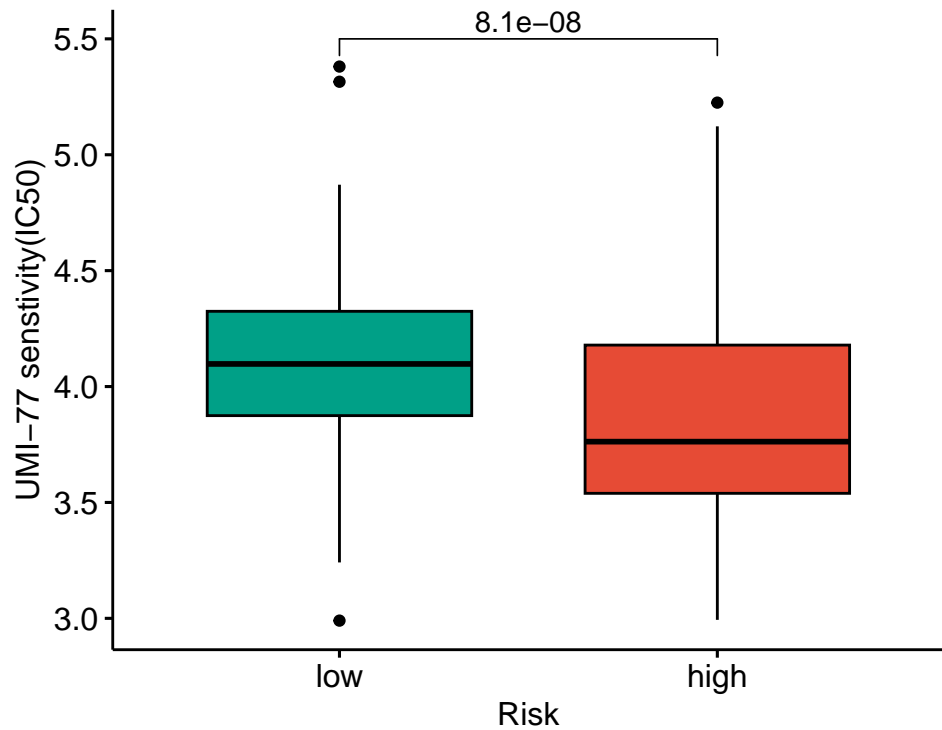

Supplement: Multimedia component 1 [file mmc1.zip › drug/drugSenstivity.UMI-77.pdf]

Risk 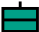 low 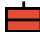 high

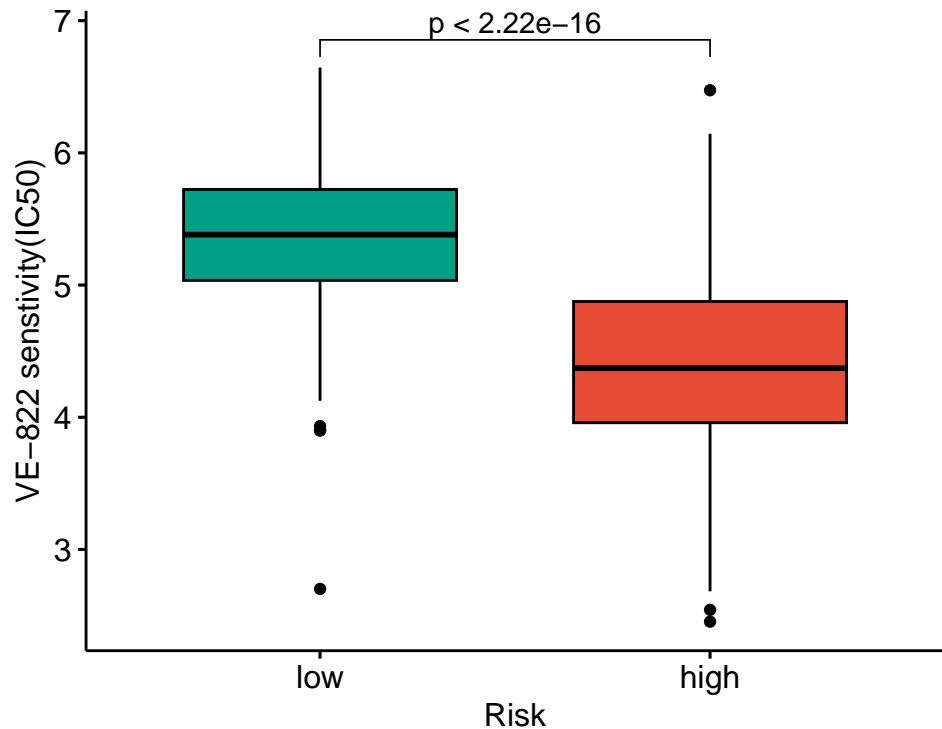

Supplement: Multimedia component 1 [file mmc1.zip › drug/drugSenstivity.VE-822.pdf]

Risk 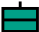 low 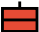 high

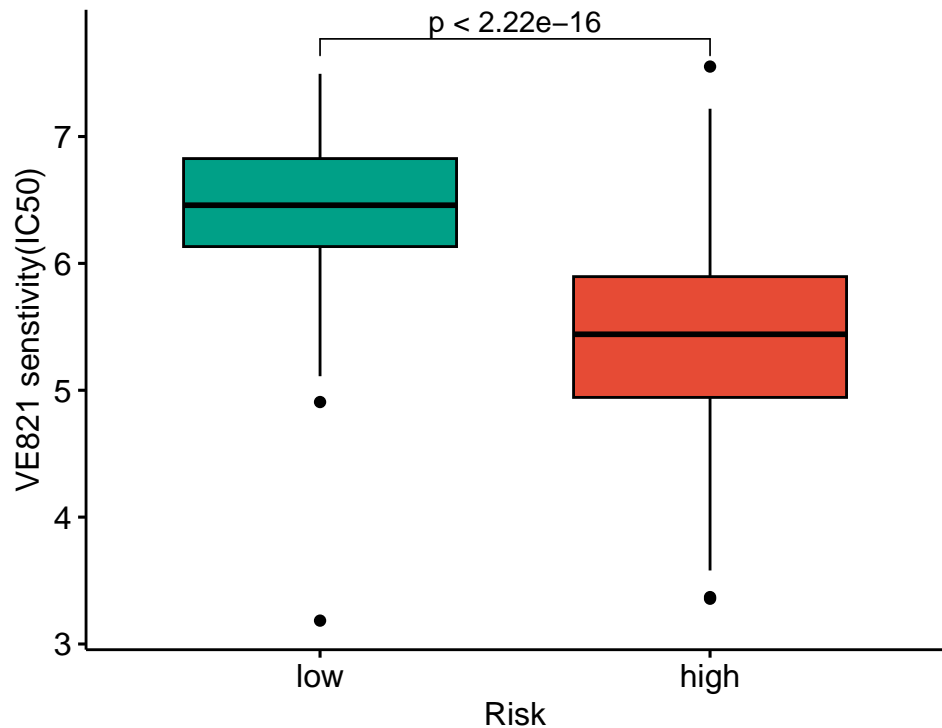

Supplement: Multimedia component 1 [file mmc1.zip › drug/drugSenstivity.VE821.pdf]

Risk 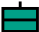 low 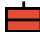 high

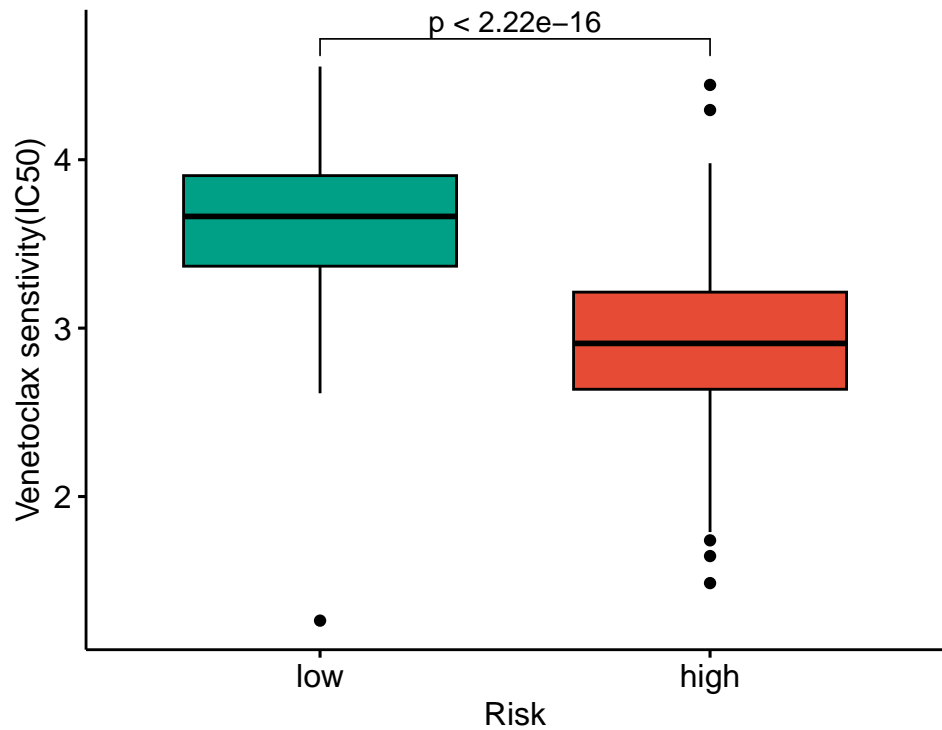

Supplement: Multimedia component 1 [file mmc1.zip › drug/drugSenstivity.Venetoclax.pdf]

Risk 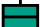 low 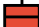 high

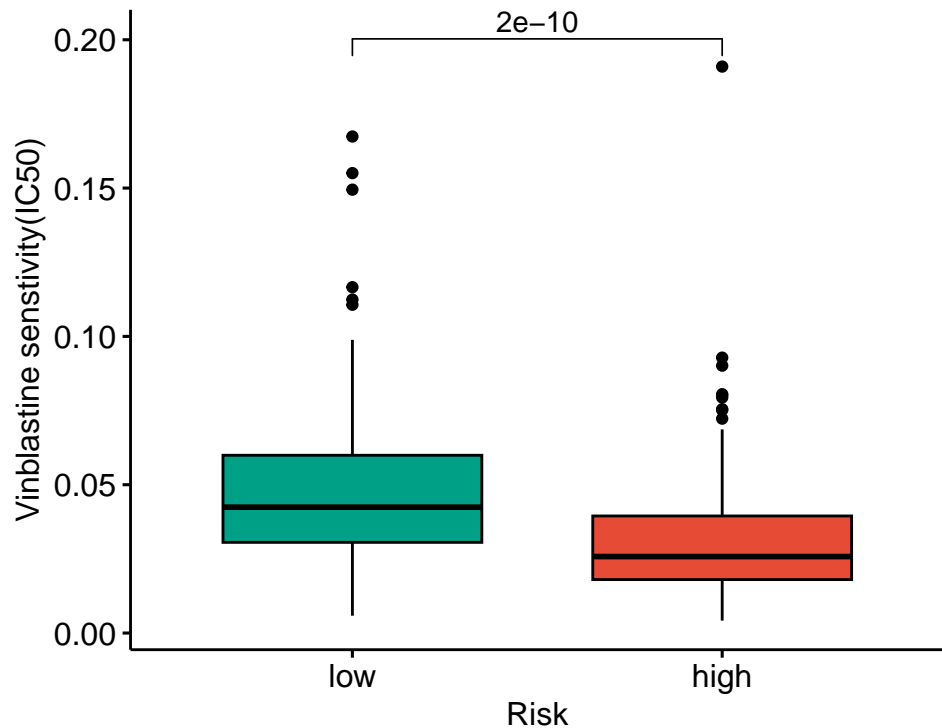

Supplement: Multimedia component 1 [file mmc1.zip › drug/drugSenstivity.Vinblastine.pdf]

Risk 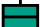 low 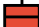 high

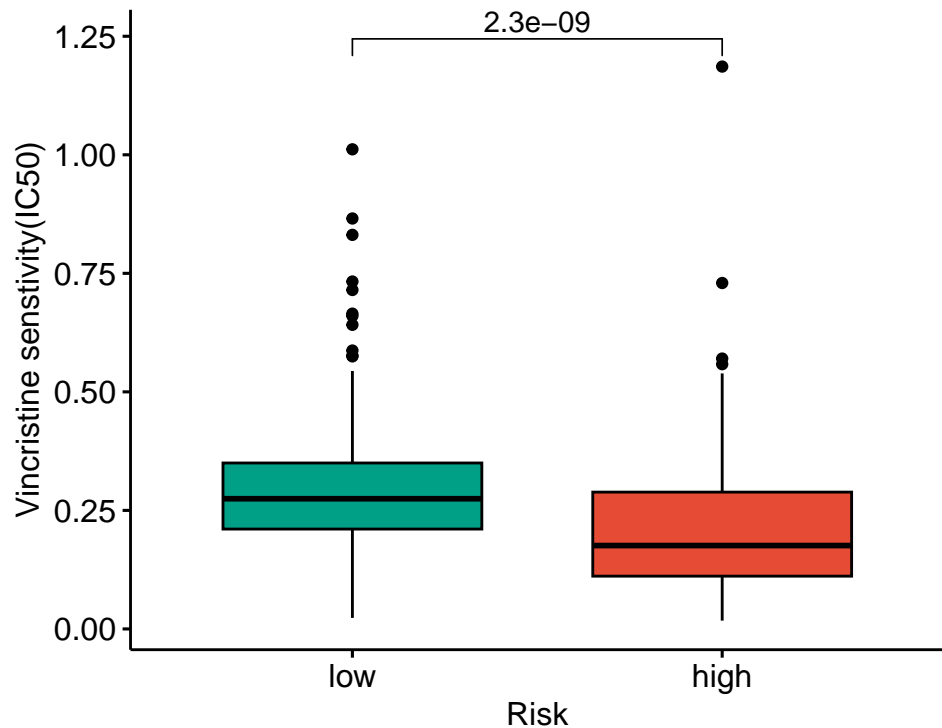

Supplement: Multimedia component 1 [file mmc1.zip › drug/drugSenstivity.Vincristine.pdf]

Risk 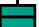 low 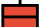 high

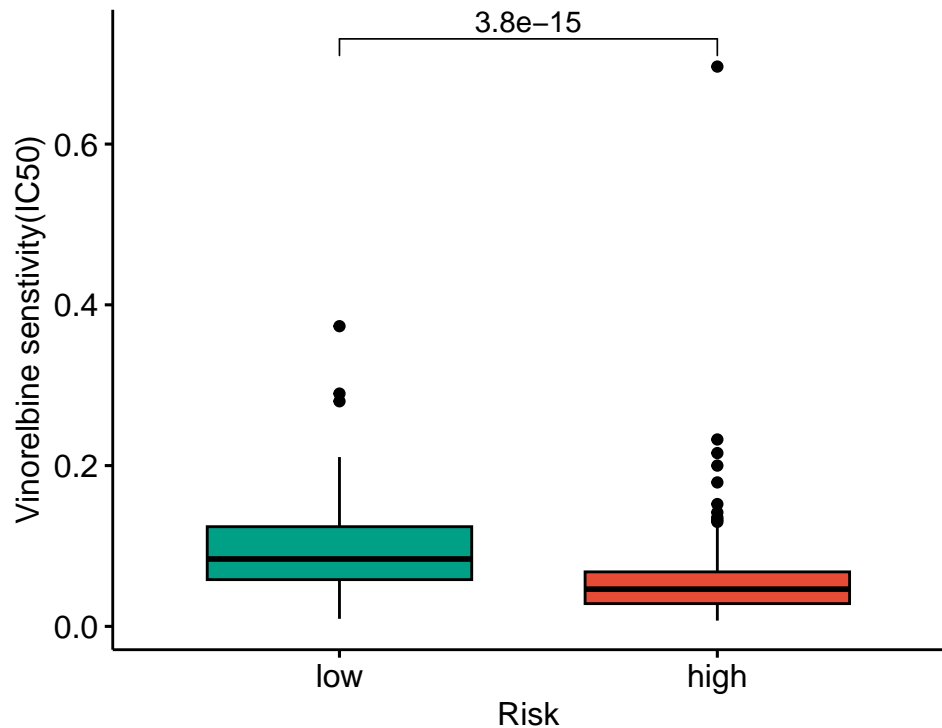

Supplement: Multimedia component 1 [file mmc1.zip › drug/drugSenstivity.Vinorelbine.pdf]

Risk 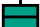 low 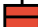 high

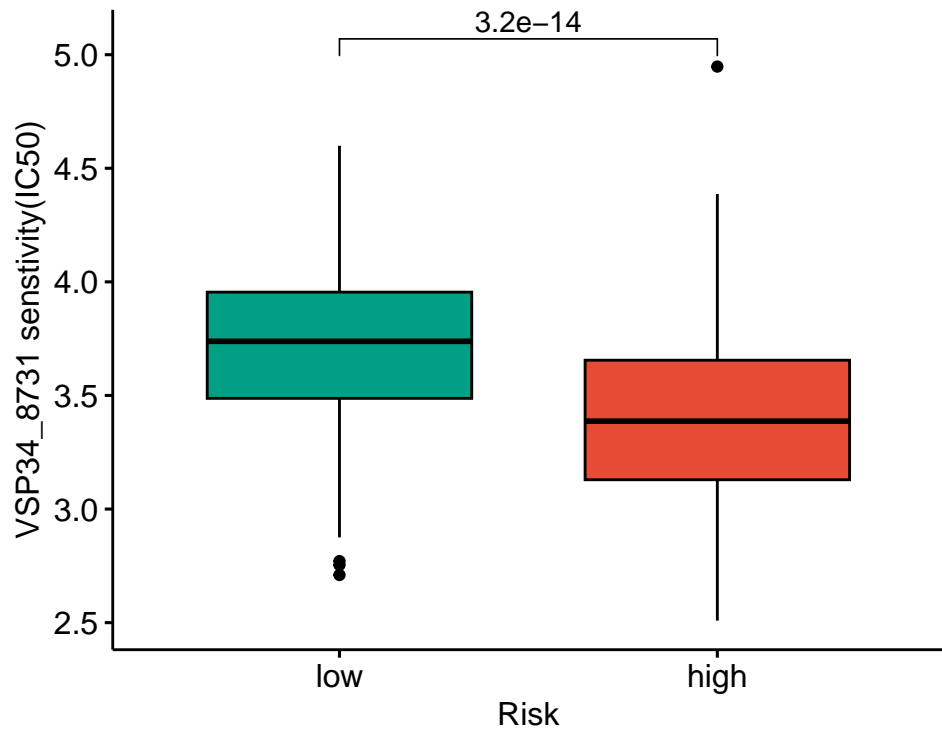

Supplement: Multimedia component 1 [file mmc1.zip › drug/drugSenstivity.VSP34_8731.pdf]

Risk 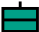 low 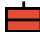 high

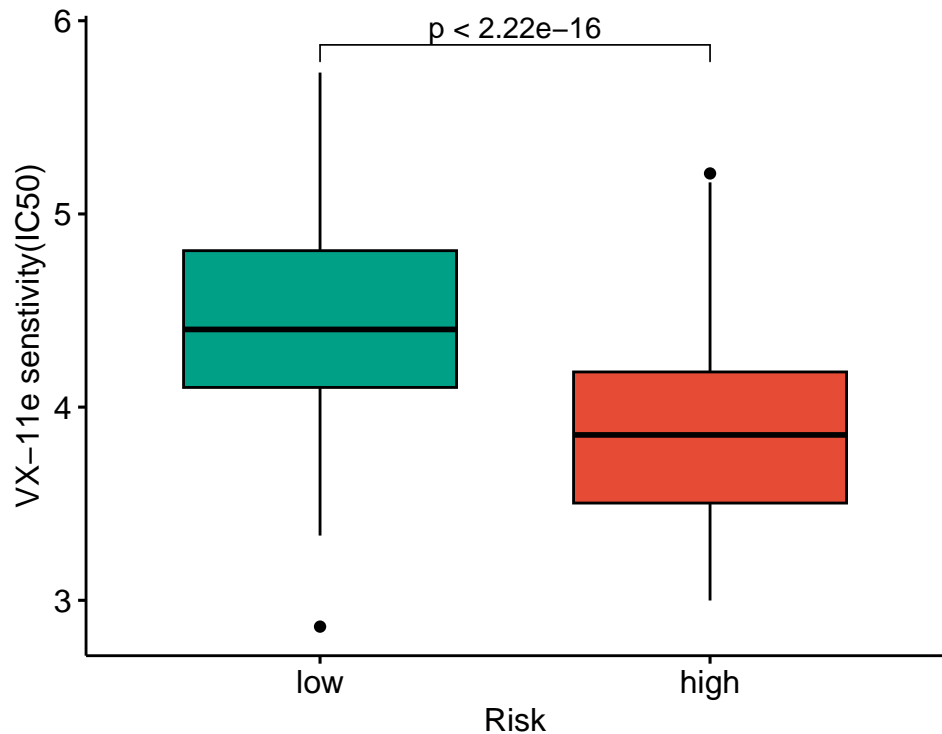

Supplement: Multimedia component 1 [file mmc1.zip › drug/drugSenstivity.VX-11e.pdf]

Risk 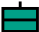 low 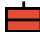 high

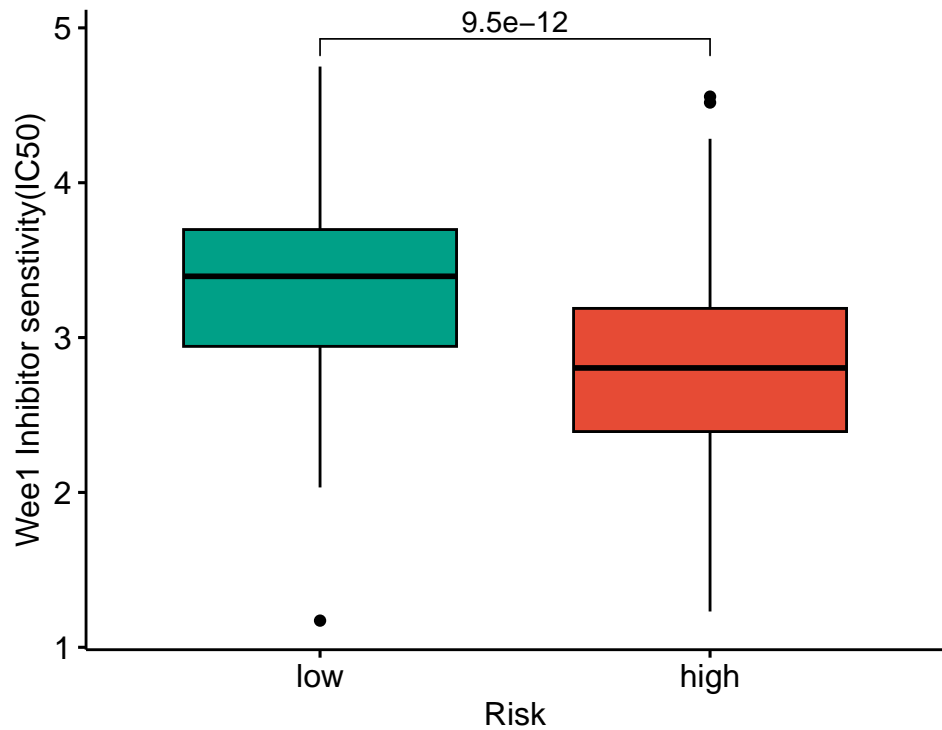

Supplement: Multimedia component 1 [file mmc1.zip › drug/drugSenstivity.Wee1 Inhibitor.pdf]

Risk 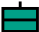 low 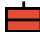 high

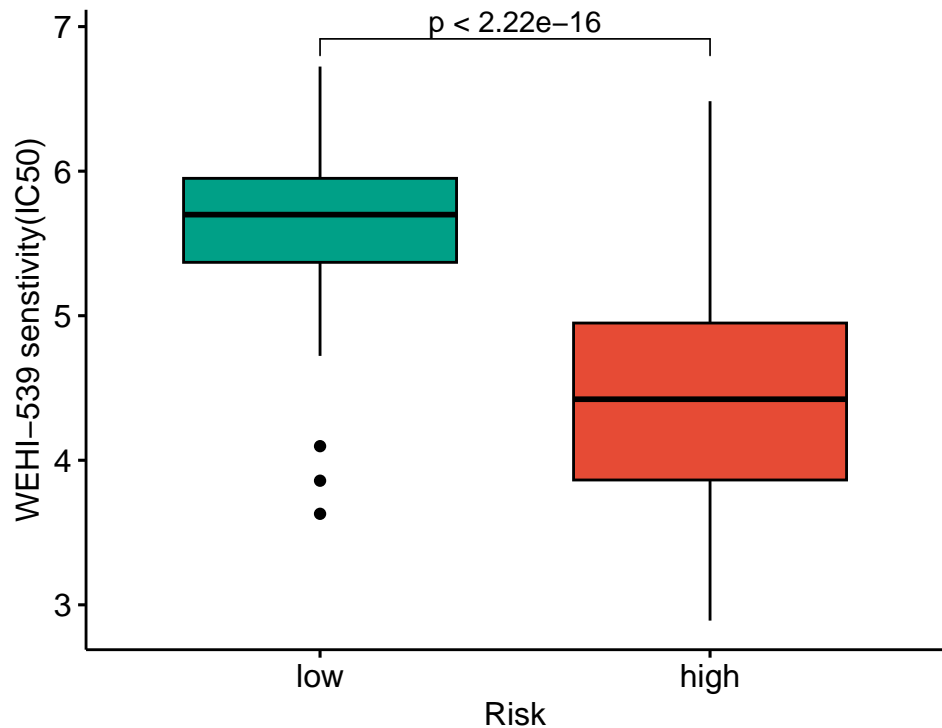

Supplement: Multimedia component 1 [file mmc1.zip › drug/drugSenstivity.WEHI-539.pdf]

Risk 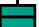 low 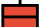 high

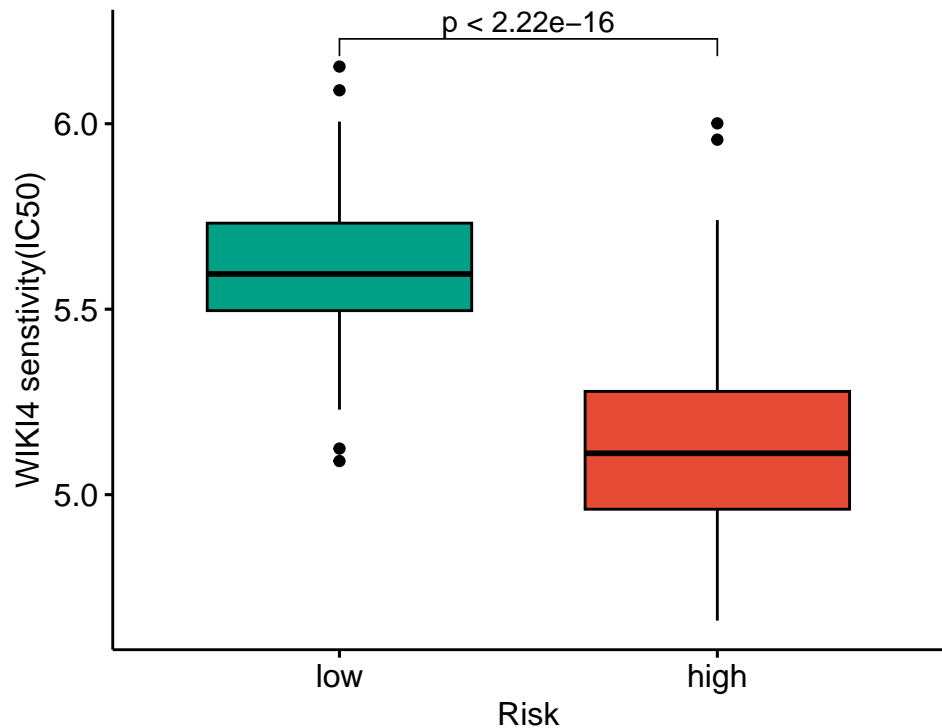

Supplement: Multimedia component 1 [file mmc1.zip › drug/drugSenstivity.WIKI4.pdf]

Risk 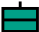 low 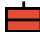 high

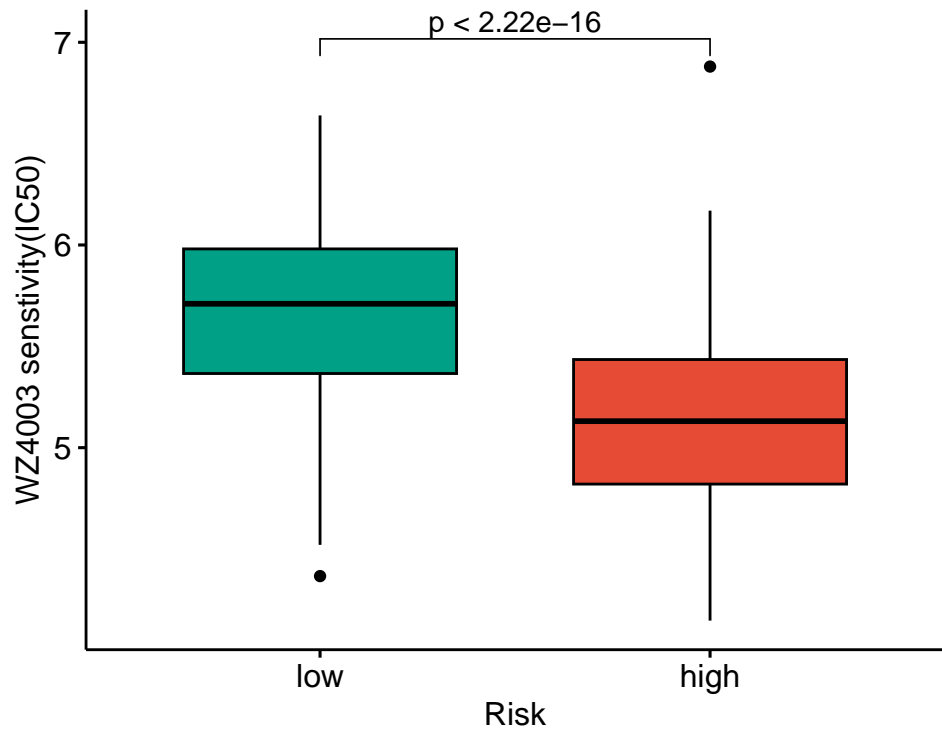

Supplement: Multimedia component 1 [file mmc1.zip › drug/drugSenstivity.WZ4003.pdf]

Risk 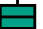 low 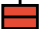 high

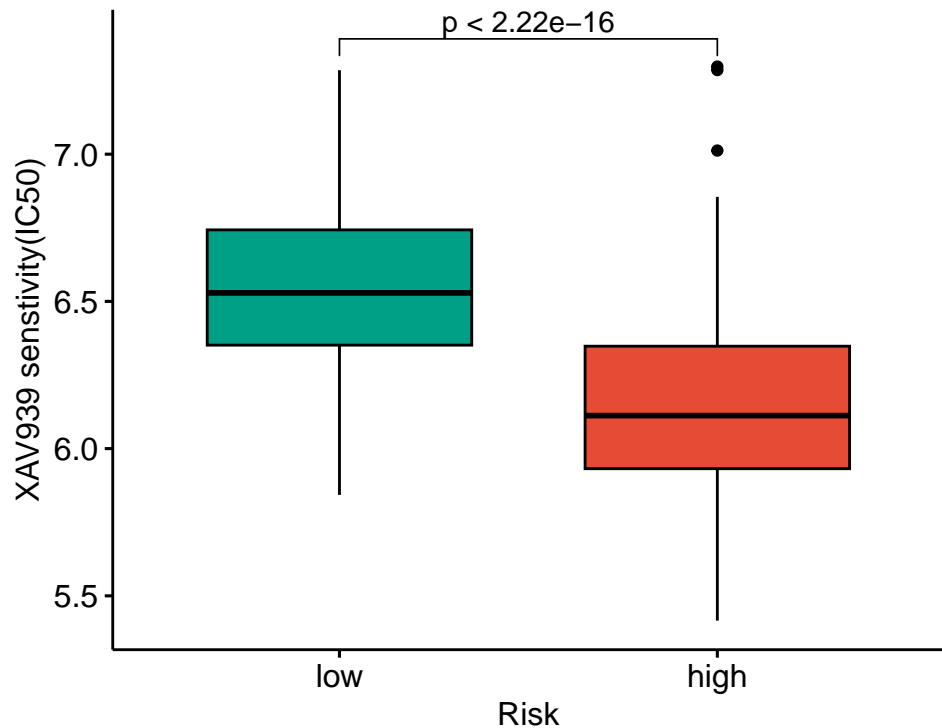

Supplement: Multimedia component 1 [file mmc1.zip › drug/drugSenstivity.XAV939.pdf]

Risk 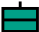 low 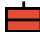 high

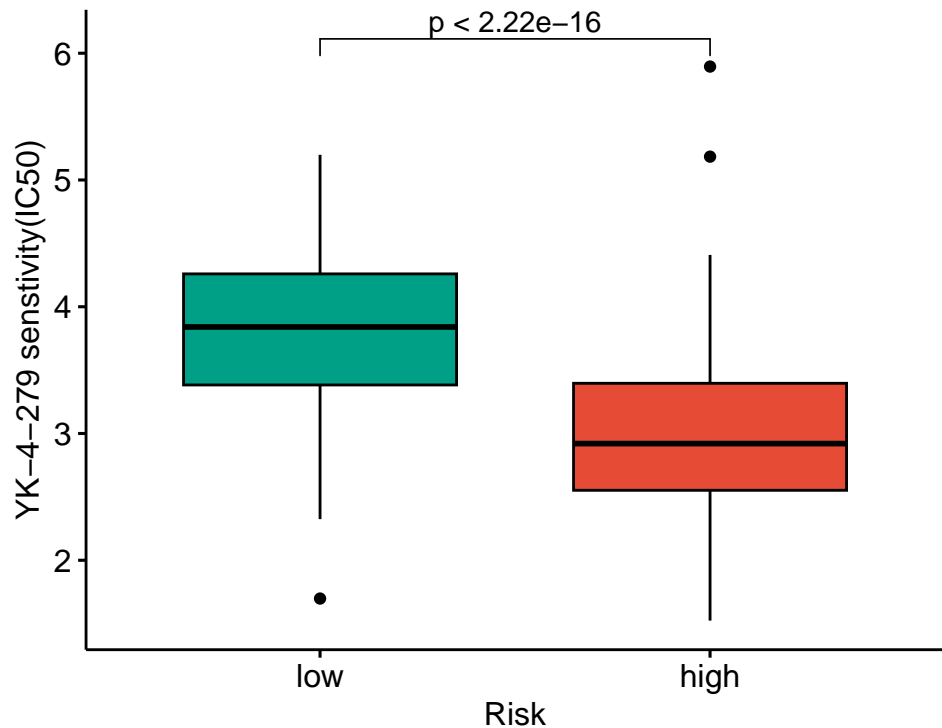

Supplement: Multimedia component 1 [file mmc1.zip › drug/drugSenstivity.YK-4-279.pdf]

Risk 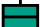 low 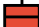 high

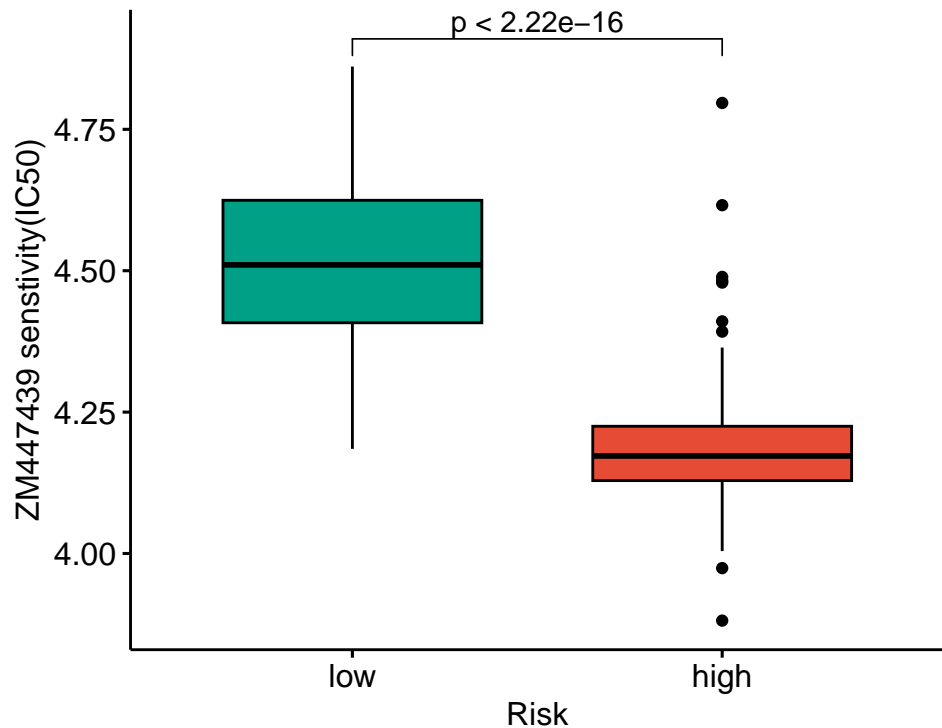

Supplement: Multimedia component 1 [file mmc1.zip › drug/drugSenstivity.ZM447439.pdf]

Risk 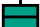 low 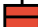 high

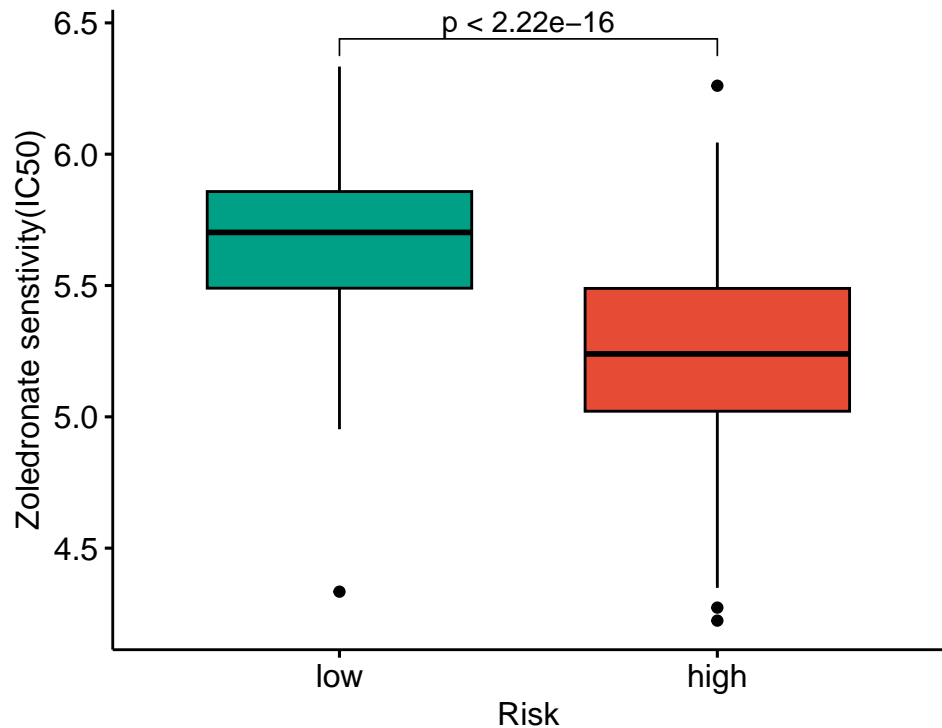

Supplement: Multimedia component 1 [file mmc1.zip › drug/drugSenstivity.Zoledronate.pdf]
