# Supplementary figures and images for: Exploration of the role of drug resistance-associated anoikis-related genes in HER2-Negative breast cancer through bioinformatics analysis (part 1 of 2)
Source: Biochem Biophys Rep. 2025 Feb 21;41:101947. doi: 10.1016/j.bbrep.2025.101947 (PMC11891708; doi:10.1016/j.bbrep.2025.101947)

Risk 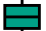 low 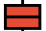 high

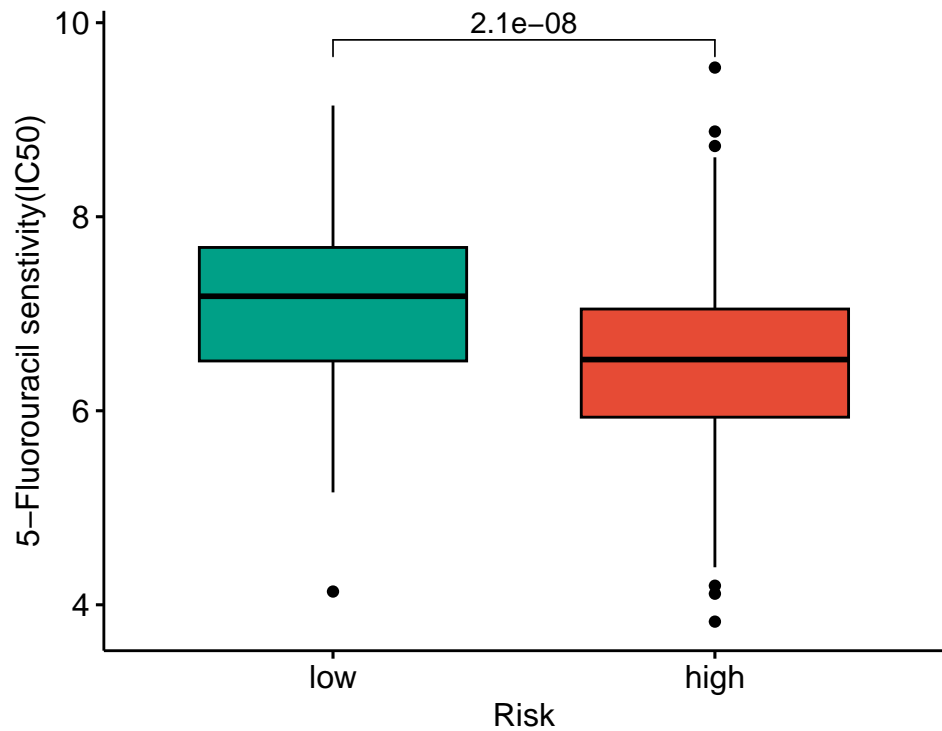

Supplement: Multimedia component 1 [file mmc1.zip › drug/drugSenstivity.5-Fluorouracil.pdf]

Risk 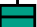 low 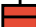 high

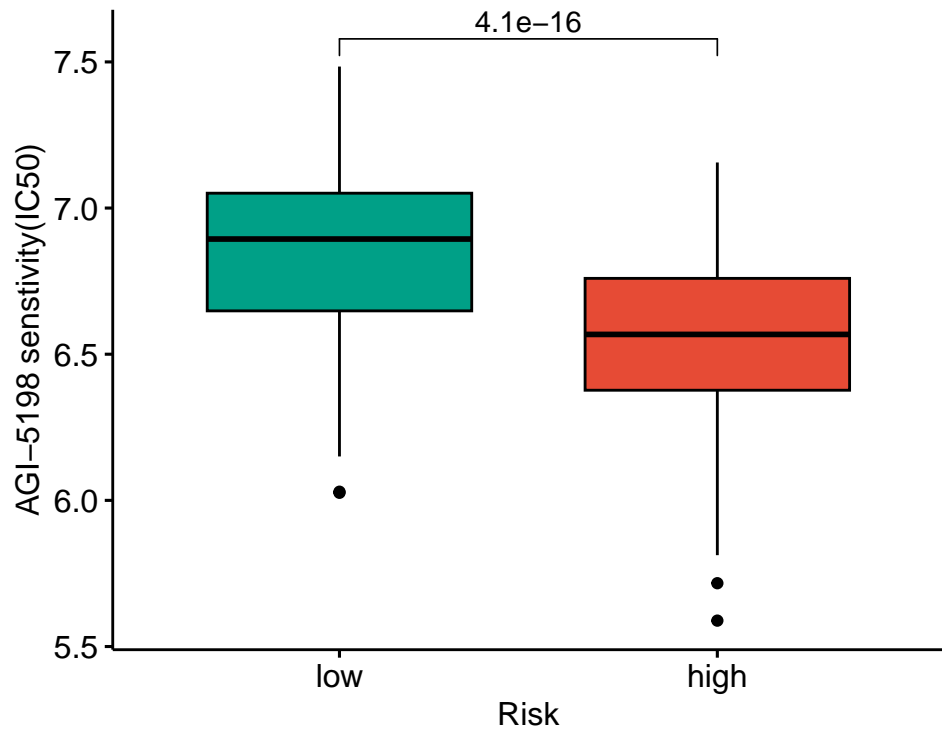

Supplement: Multimedia component 1 [file mmc1.zip › drug/drugSenstivity.AGI-5198.pdf]

Risk 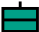 low 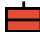 high

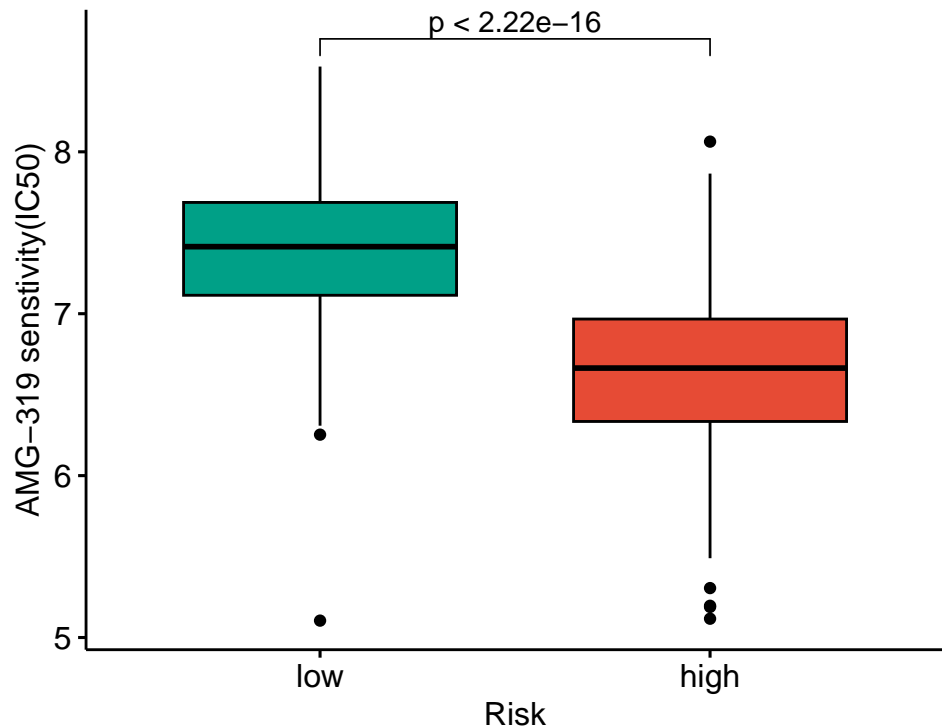

Supplement: Multimedia component 1 [file mmc1.zip › drug/drugSenstivity.AMG-319.pdf]

Risk 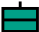 low 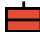 high

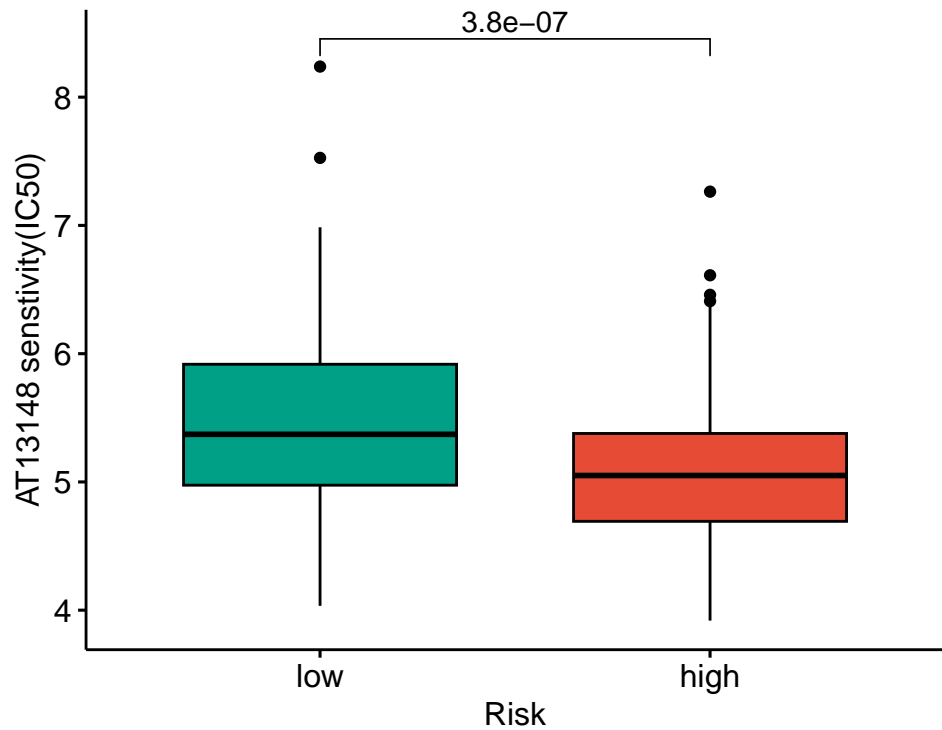

Supplement: Multimedia component 1 [file mmc1.zip › drug/drugSenstivity.AT13148.pdf]

Risk 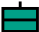 low 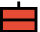 high

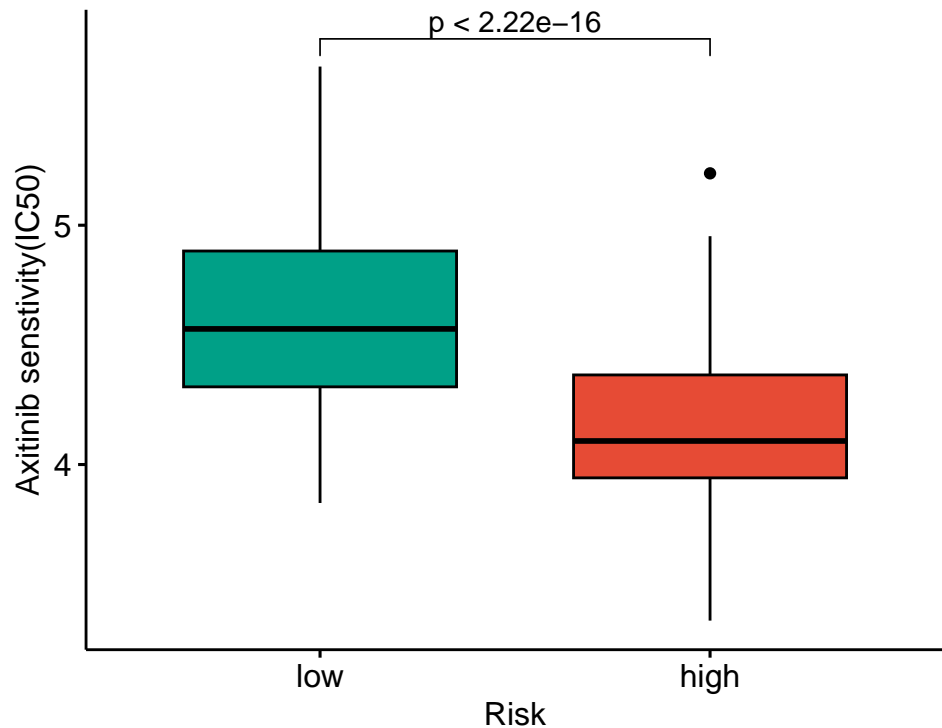

Supplement: Multimedia component 1 [file mmc1.zip › drug/drugSenstivity.Axitinib.pdf]

Risk 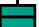 low 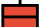 high

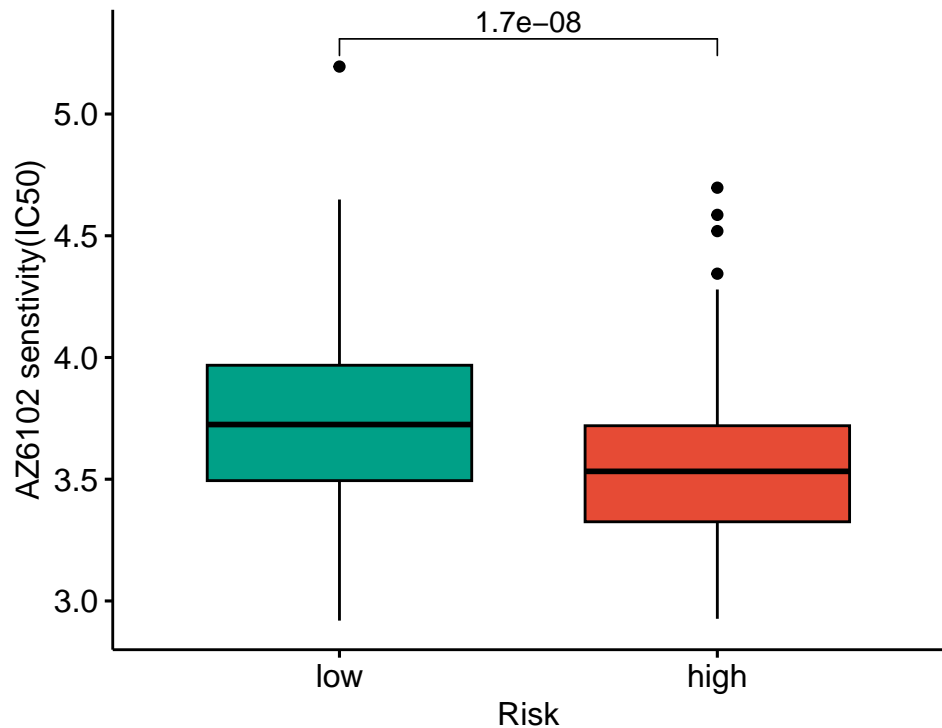

Supplement: Multimedia component 1 [file mmc1.zip › drug/drugSenstivity.AZ6102.pdf]

Risk 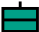 low 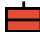 high

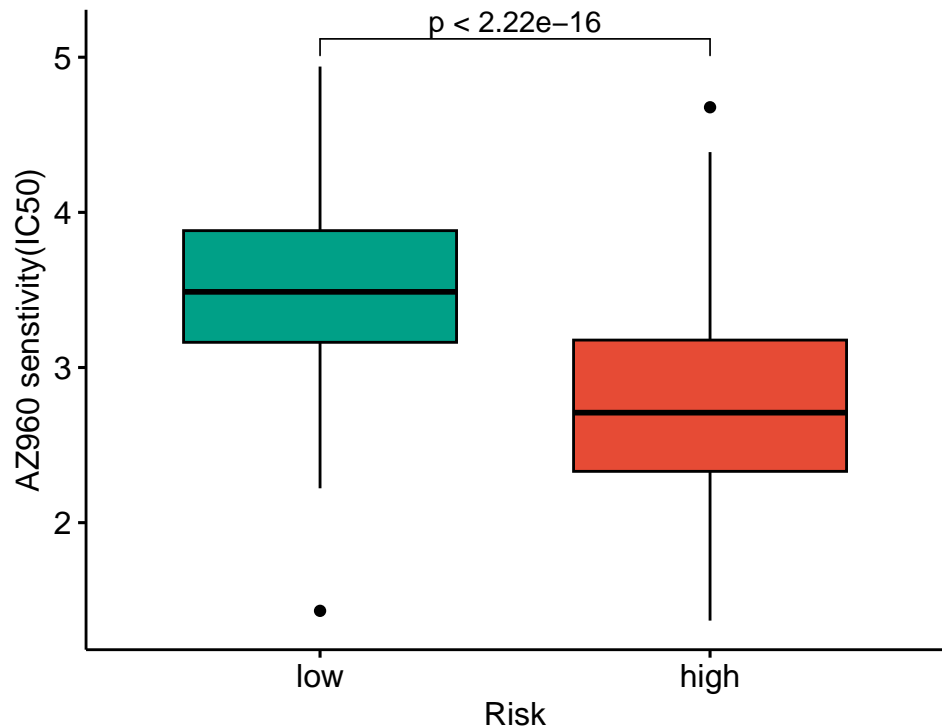

Supplement: Multimedia component 1 [file mmc1.zip › drug/drugSenstivity.AZ960.pdf]

Risk 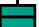 low 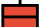 high

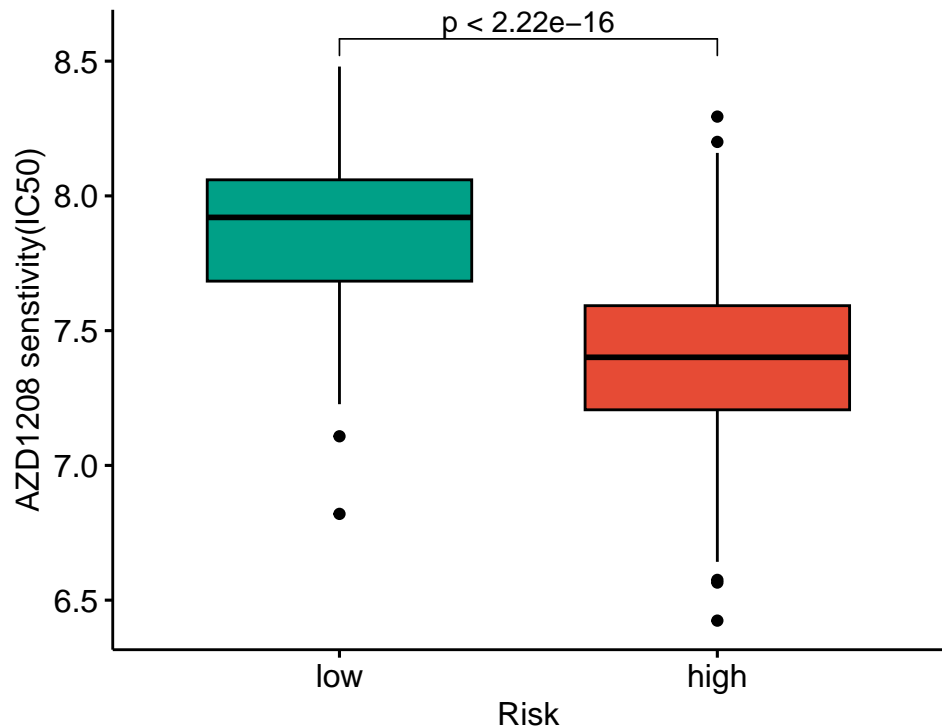

Supplement: Multimedia component 1 [file mmc1.zip › drug/drugSenstivity.AZD1208.pdf]

Risk 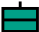 low 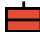 high

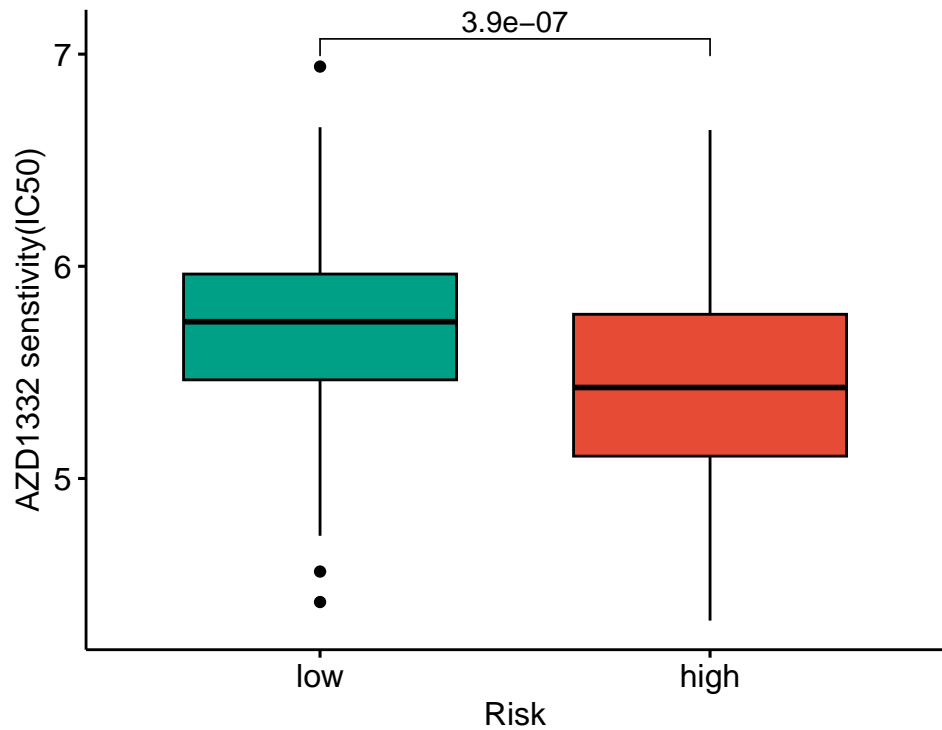

Supplement: Multimedia component 1 [file mmc1.zip › drug/drugSenstivity.AZD1332.pdf]

Risk 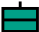 low 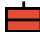 high

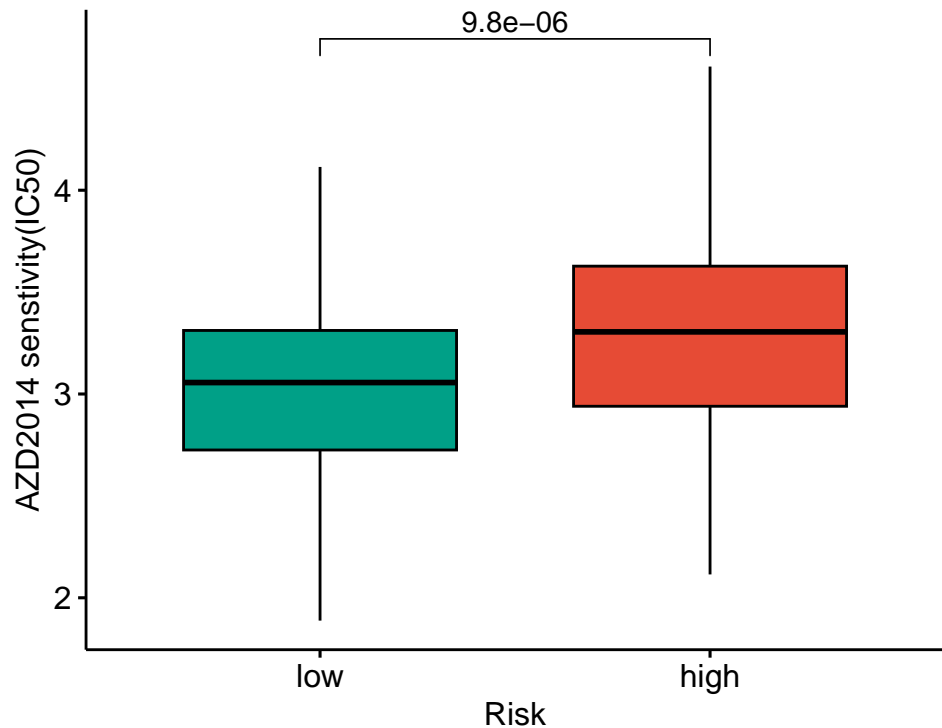

Supplement: Multimedia component 1 [file mmc1.zip › drug/drugSenstivity.AZD2014.pdf]

Risk 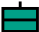 low 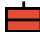 high

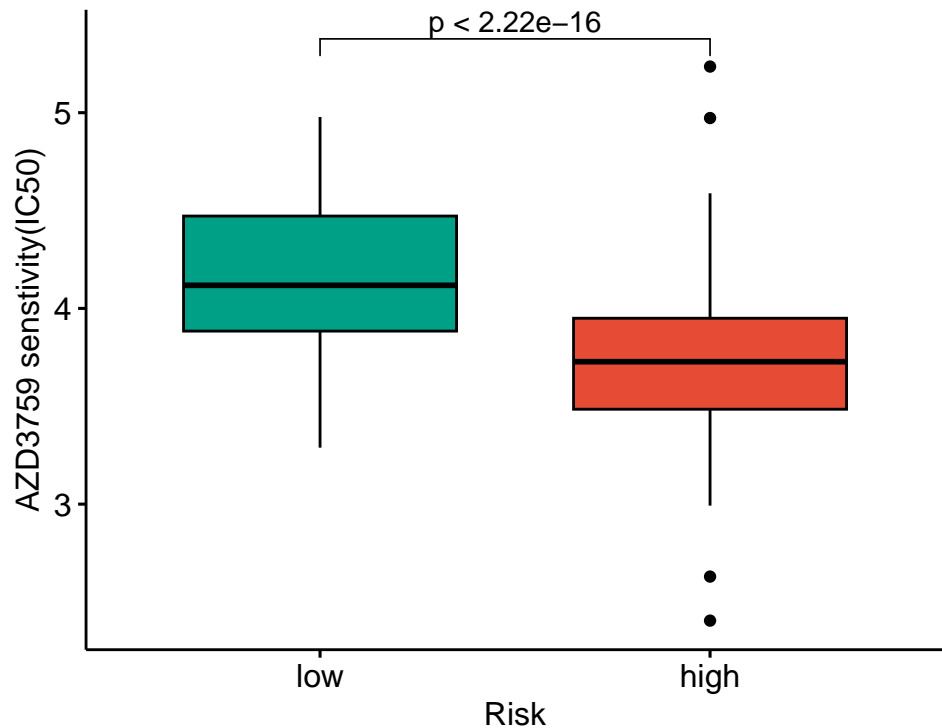

Supplement: Multimedia component 1 [file mmc1.zip › drug/drugSenstivity.AZD3759.pdf]

Risk 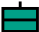 low 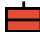 high

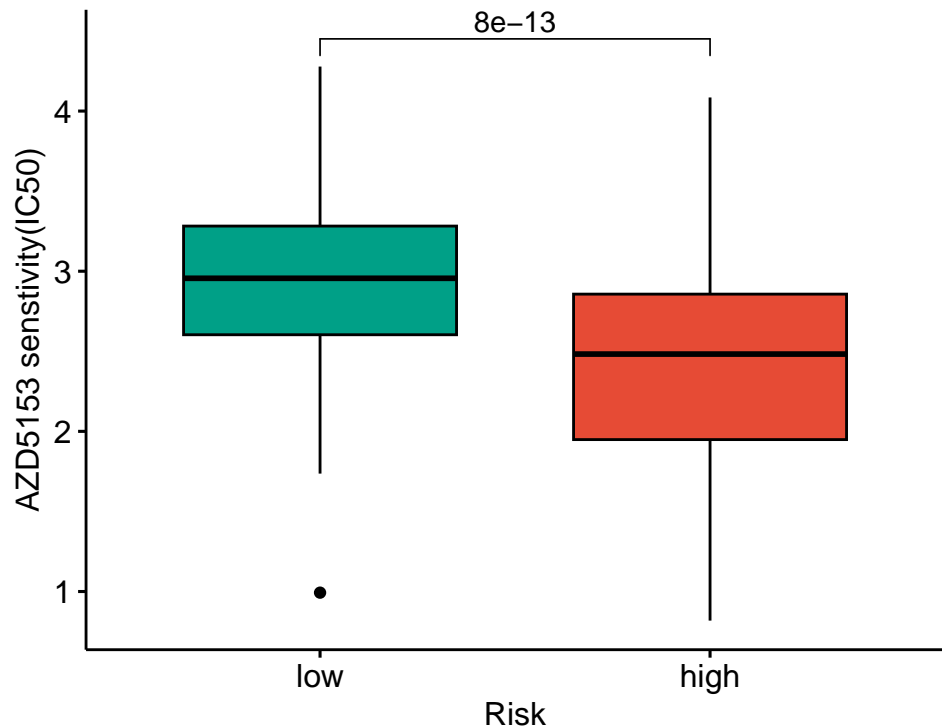

Supplement: Multimedia component 1 [file mmc1.zip › drug/drugSenstivity.AZD5153.pdf]

Risk 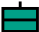 low 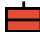 high

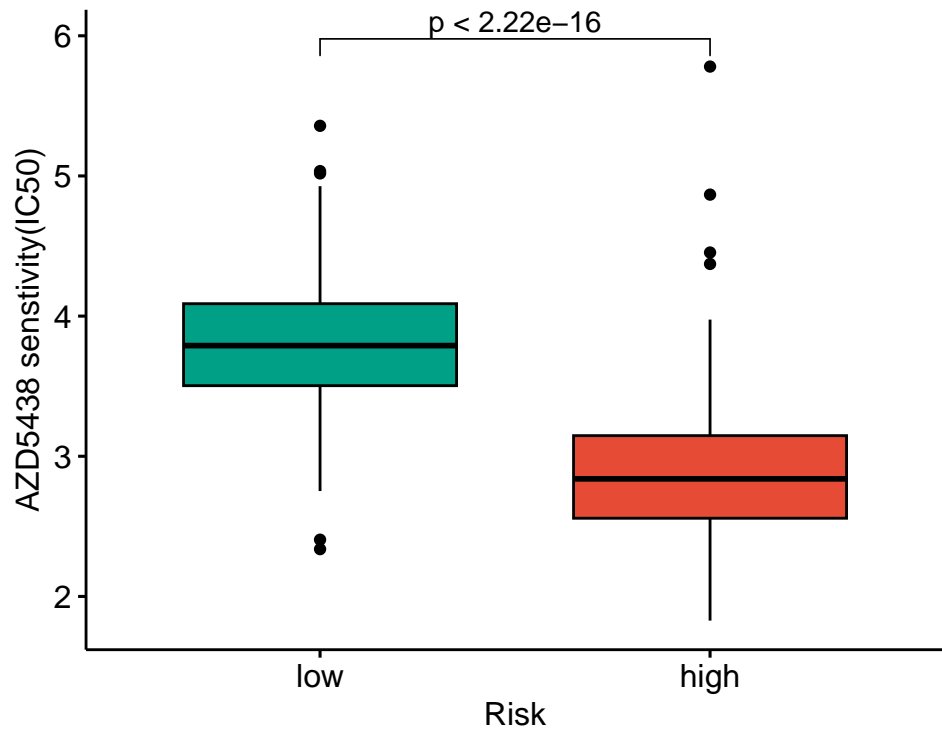

Supplement: Multimedia component 1 [file mmc1.zip › drug/drugSenstivity.AZD5438.pdf]

Risk 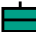 low 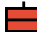 high

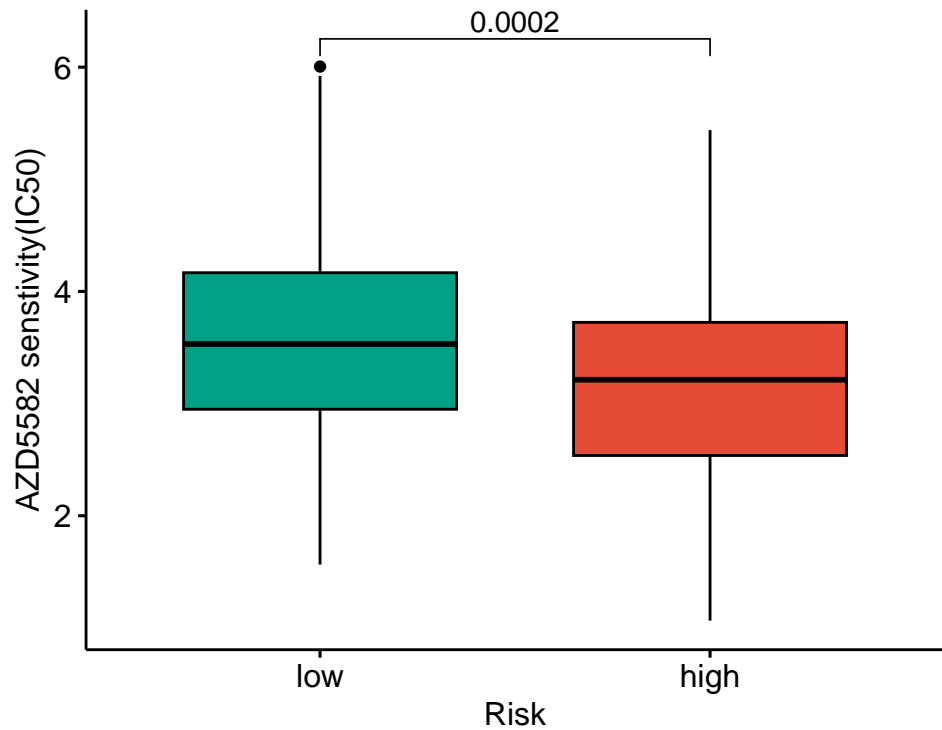

Supplement: Multimedia component 1 [file mmc1.zip › drug/drugSenstivity.AZD5582.pdf]

Risk 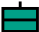 low 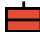 high

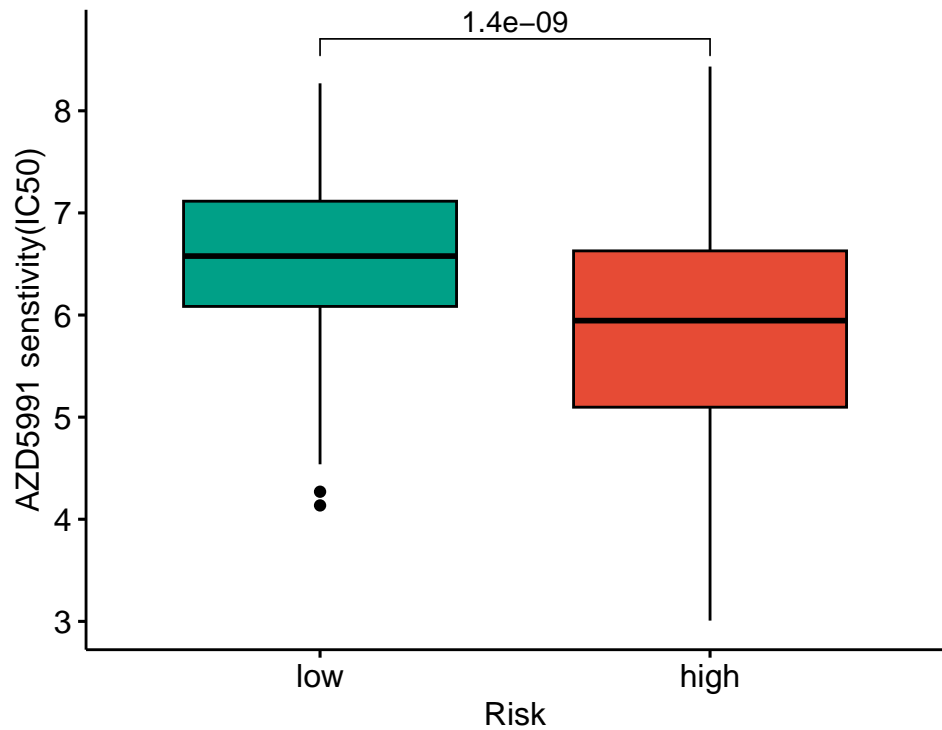

Supplement: Multimedia component 1 [file mmc1.zip › drug/drugSenstivity.AZD5991.pdf]

Risk 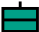 low 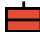 high

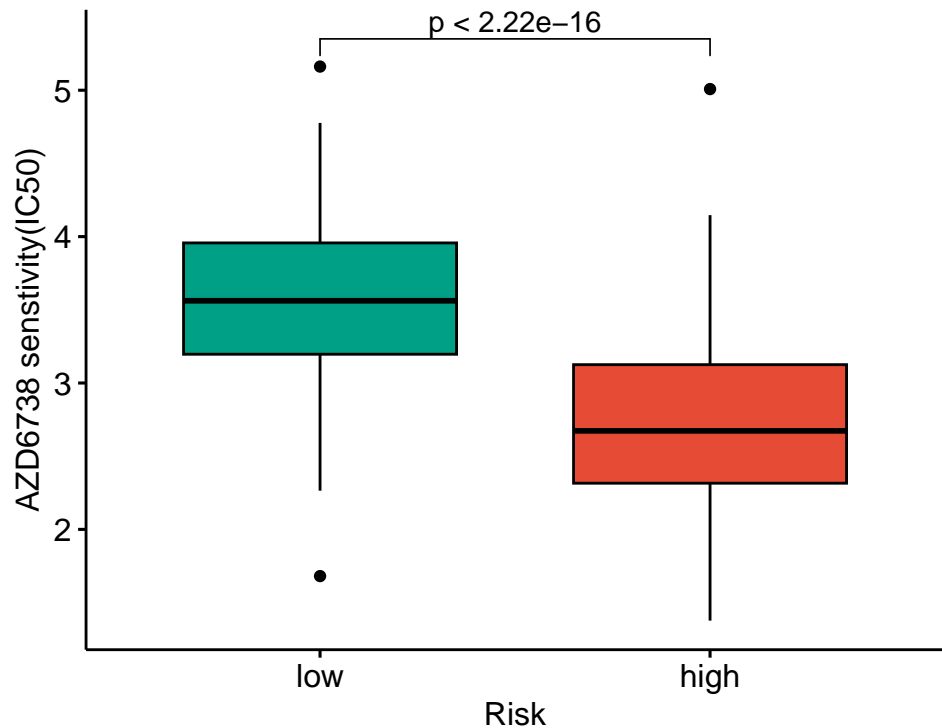

Supplement: Multimedia component 1 [file mmc1.zip › drug/drugSenstivity.AZD6738.pdf]

Risk 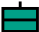 low 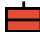 high

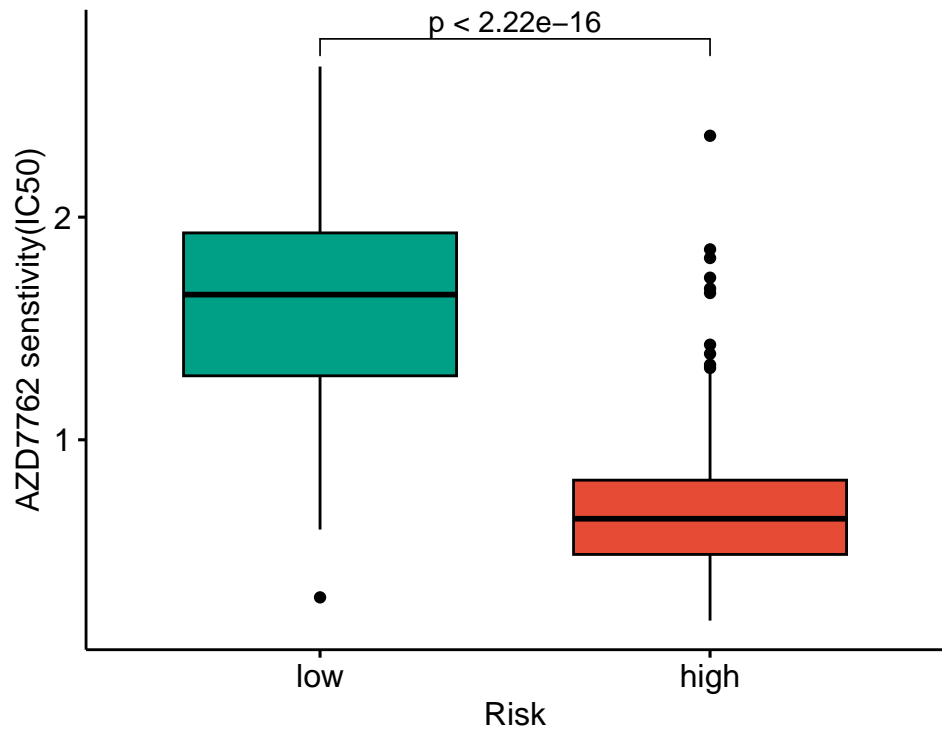

Supplement: Multimedia component 1 [file mmc1.zip › drug/drugSenstivity.AZD7762.pdf]

Risk 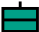 low 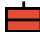 high

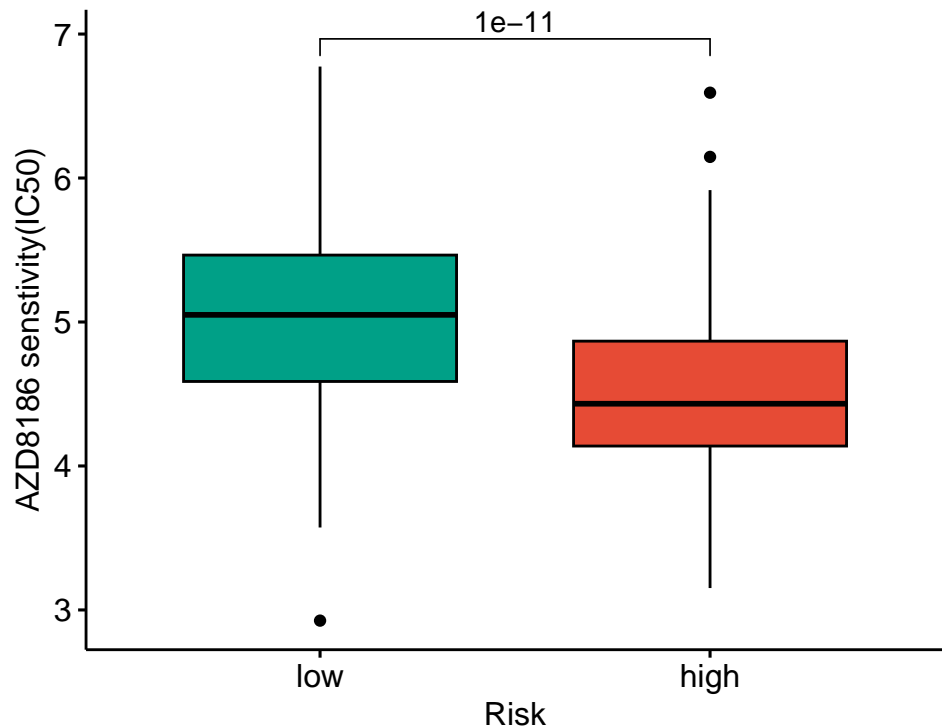

Supplement: Multimedia component 1 [file mmc1.zip › drug/drugSenstivity.AZD8186.pdf]

Risk low high

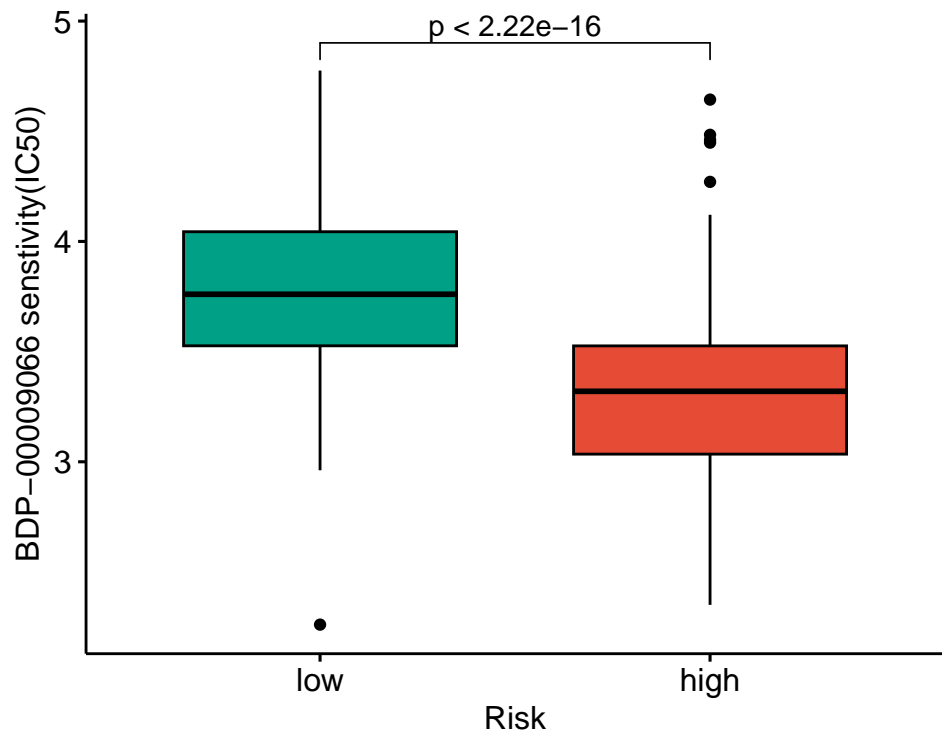

Supplement: Multimedia component 1 [file mmc1.zip › drug/drugSenstivity.BDP-00009066.pdf]

Risk 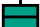 low 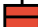 high

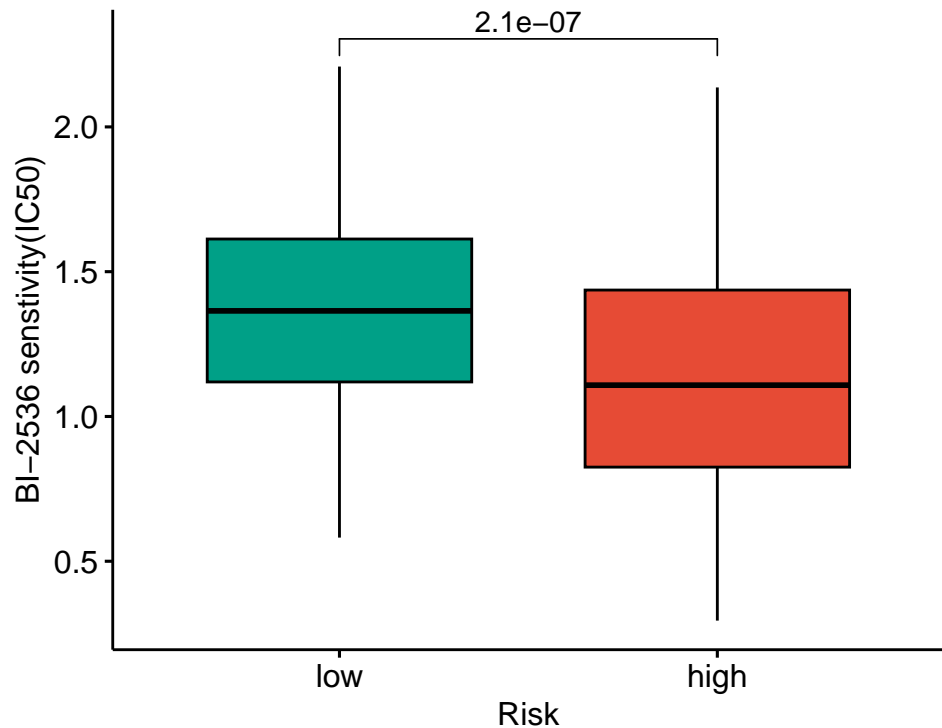

Supplement: Multimedia component 1 [file mmc1.zip › drug/drugSenstivity.BI-2536.pdf]

Risk 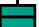 low 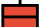 high

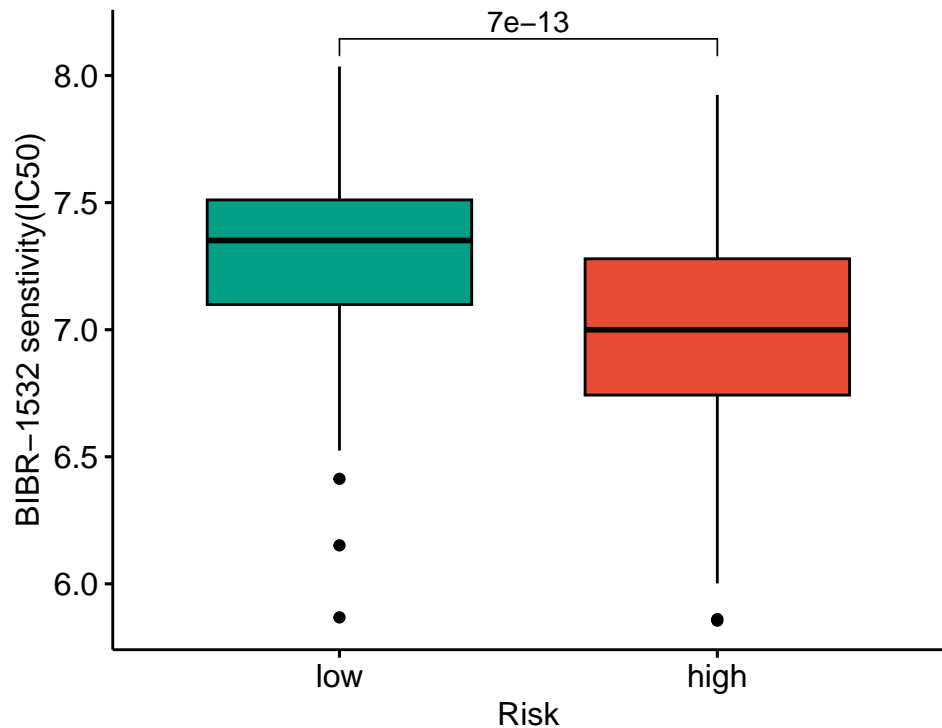

Supplement: Multimedia component 1 [file mmc1.zip › drug/drugSenstivity.BIBR-1532.pdf]

Risk 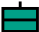 low 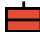 high

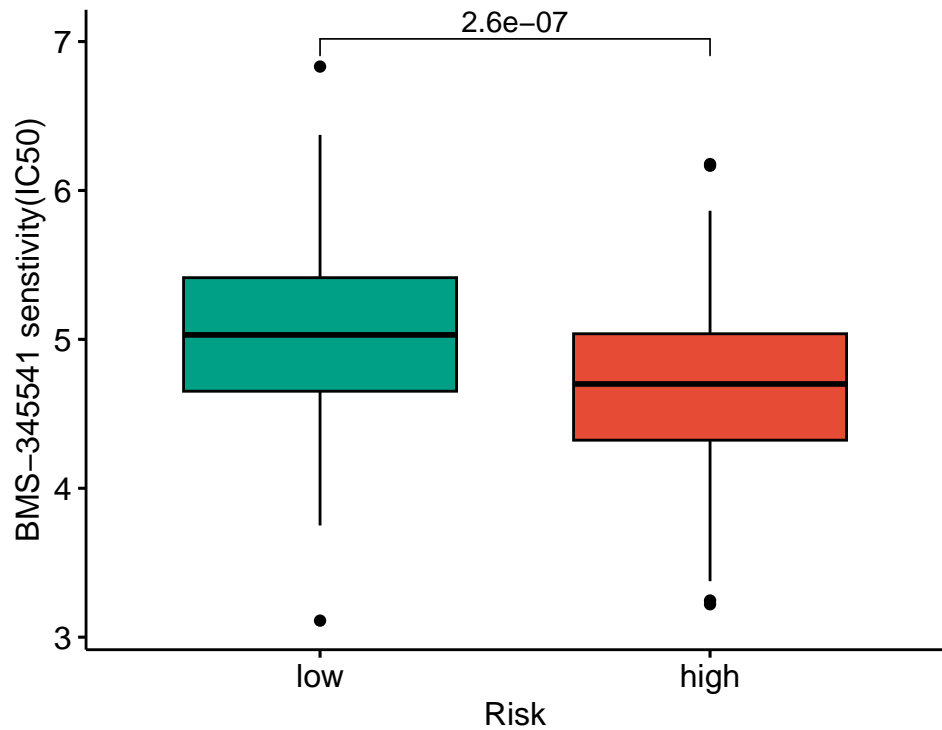

Supplement: Multimedia component 1 [file mmc1.zip › drug/drugSenstivity.BMS-345541.pdf]

Risk 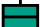 low 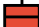 high

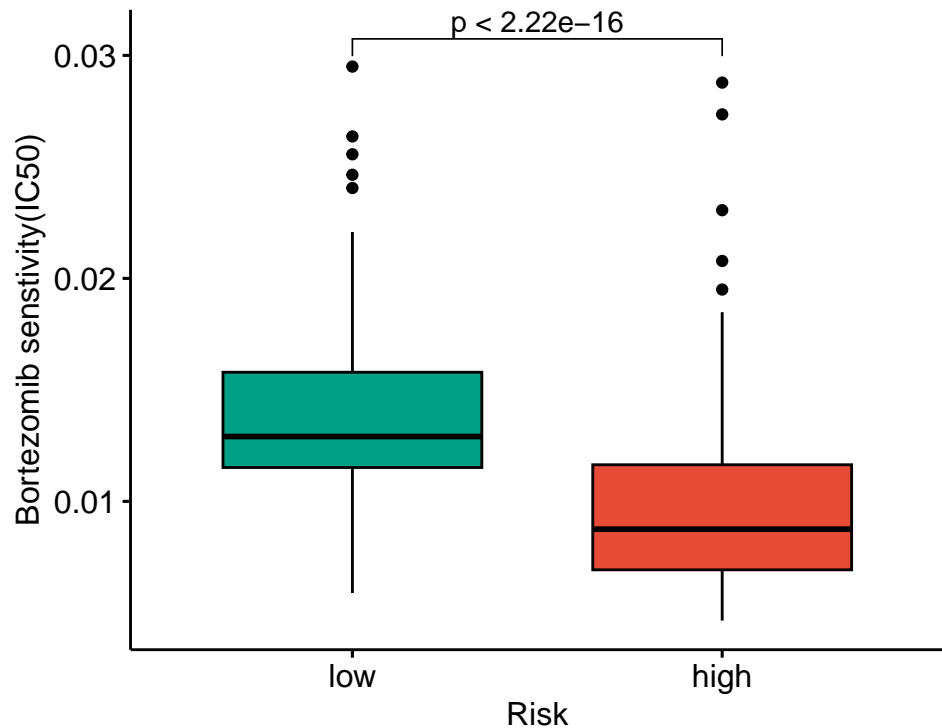

Supplement: Multimedia component 1 [file mmc1.zip › drug/drugSenstivity.Bortezomib.pdf]

Risk 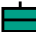 low 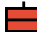 high

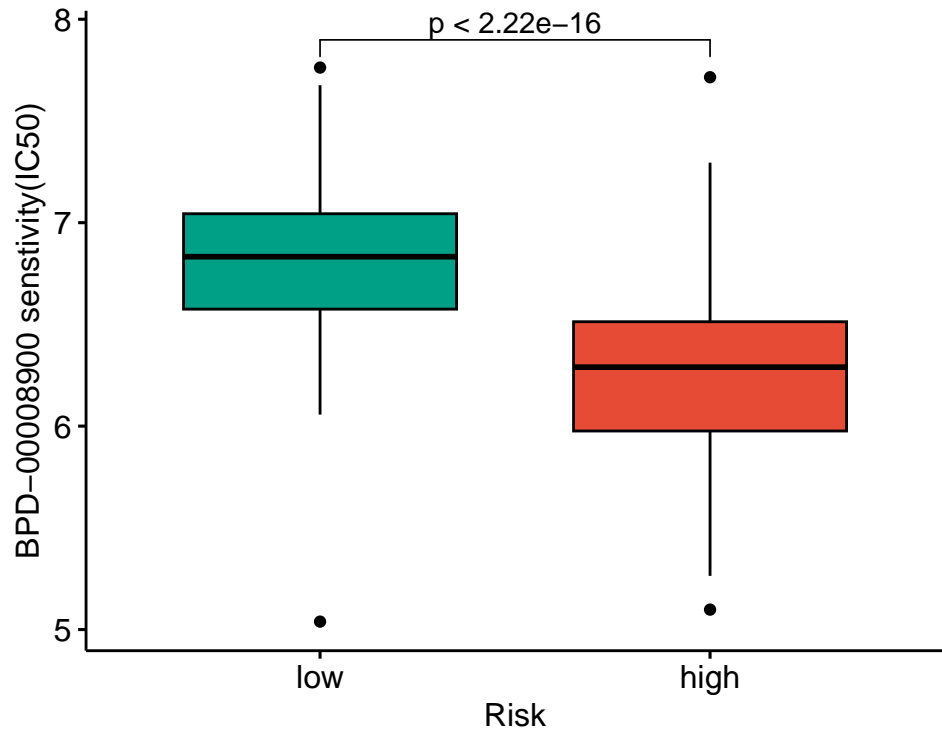

Supplement: Multimedia component 1 [file mmc1.zip › drug/drugSenstivity.BPD-00008900.pdf]

Risk 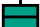 low 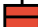 high

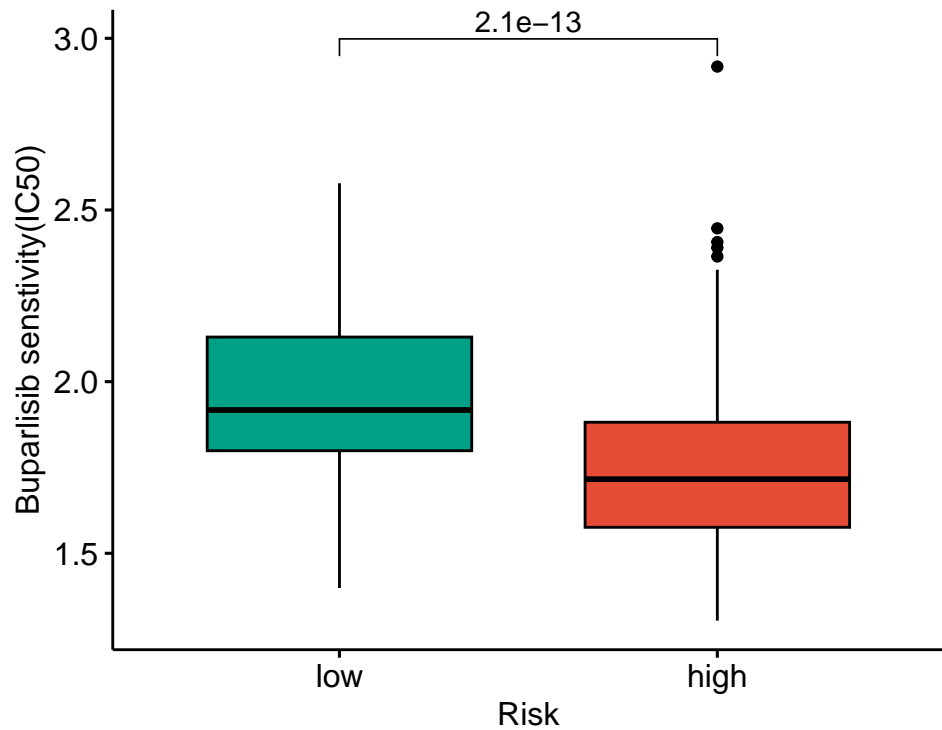

Supplement: Multimedia component 1 [file mmc1.zip › drug/drugSenstivity.Buparlisib.pdf]

Risk 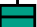 low 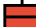 high

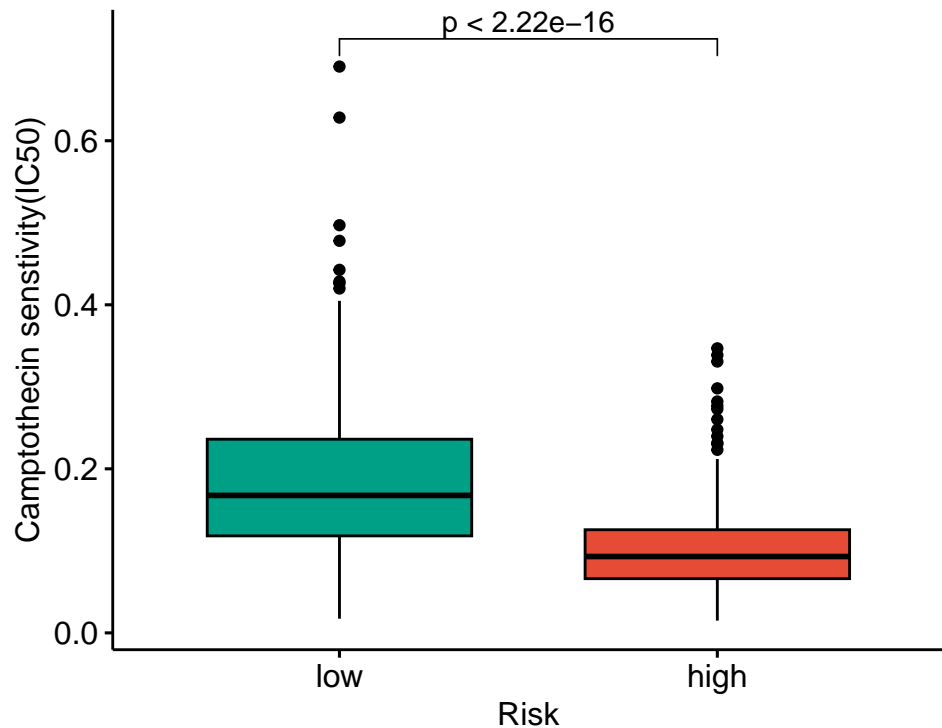

Supplement: Multimedia component 1 [file mmc1.zip › drug/drugSenstivity.Camptothecin.pdf]

Risk 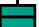 low 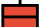 high

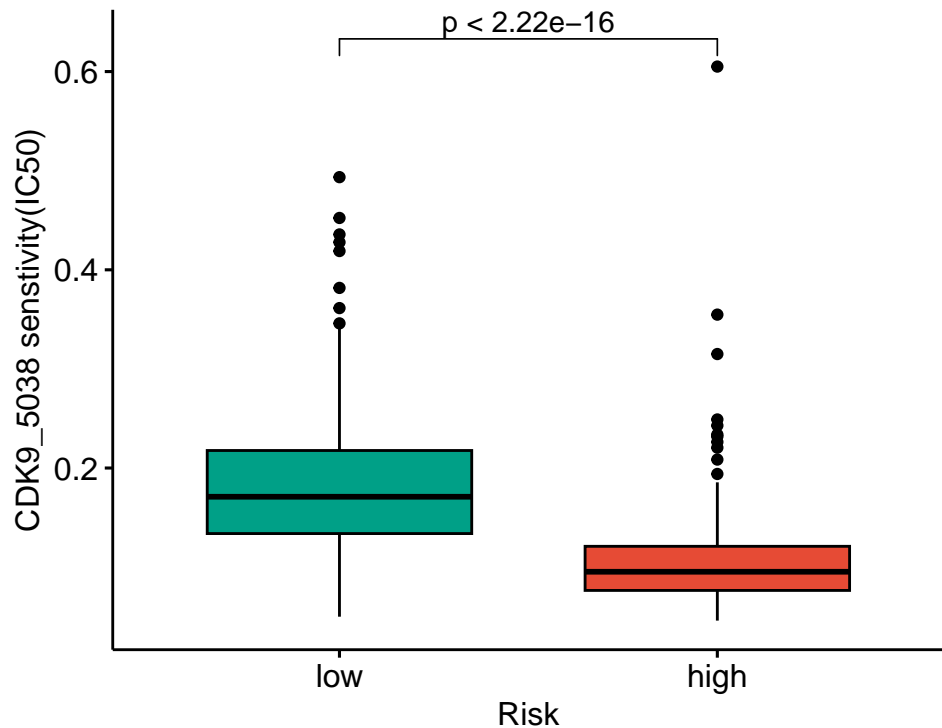

Supplement: Multimedia component 1 [file mmc1.zip › drug/drugSenstivity.CDK9_5038.pdf]

Risk 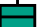 low 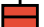 high

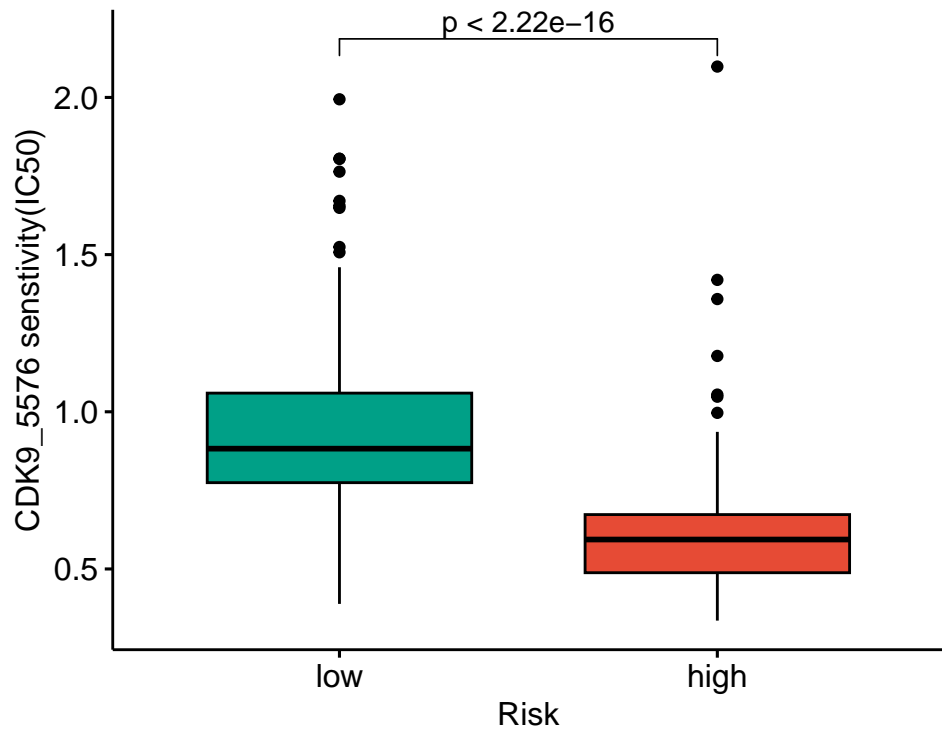

Supplement: Multimedia component 1 [file mmc1.zip › drug/drugSenstivity.CDK9_5576.pdf]

Risk 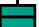 low 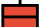 high

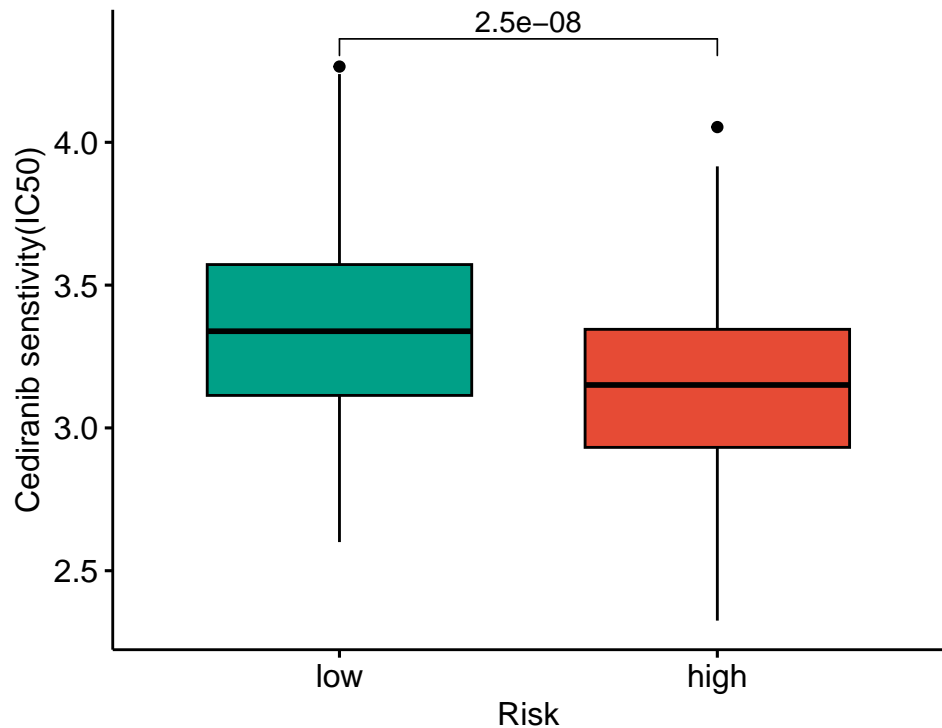

Supplement: Multimedia component 1 [file mmc1.zip › drug/drugSenstivity.Cediranib.pdf]

Risk 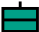 low 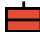 high

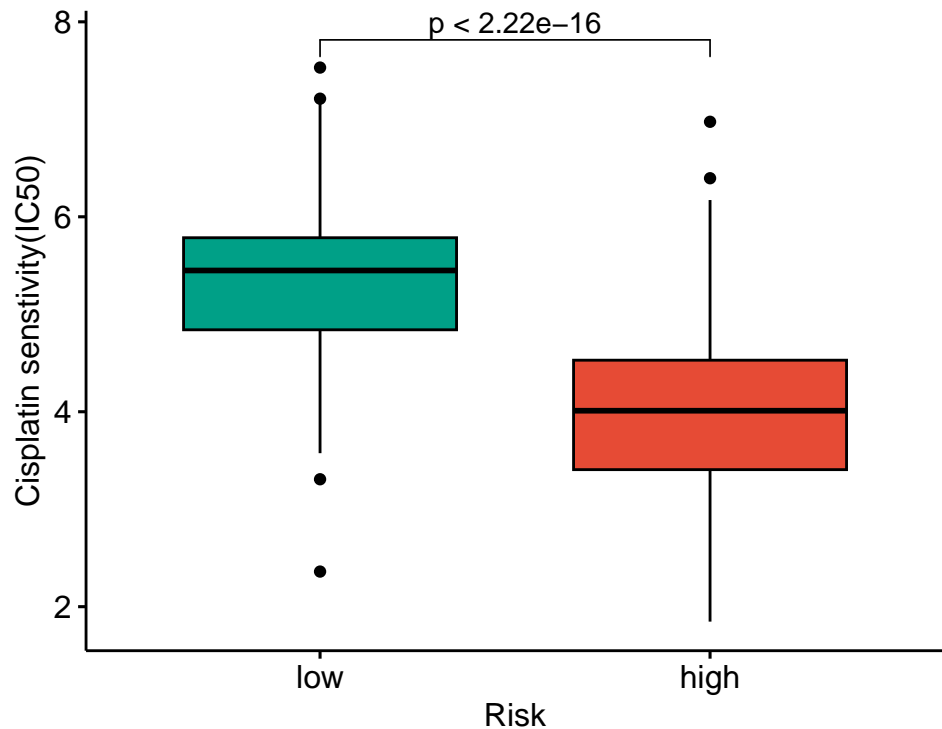

Supplement: Multimedia component 1 [file mmc1.zip › drug/drugSenstivity.Cisplatin.pdf]

Risk 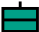 low 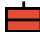 high

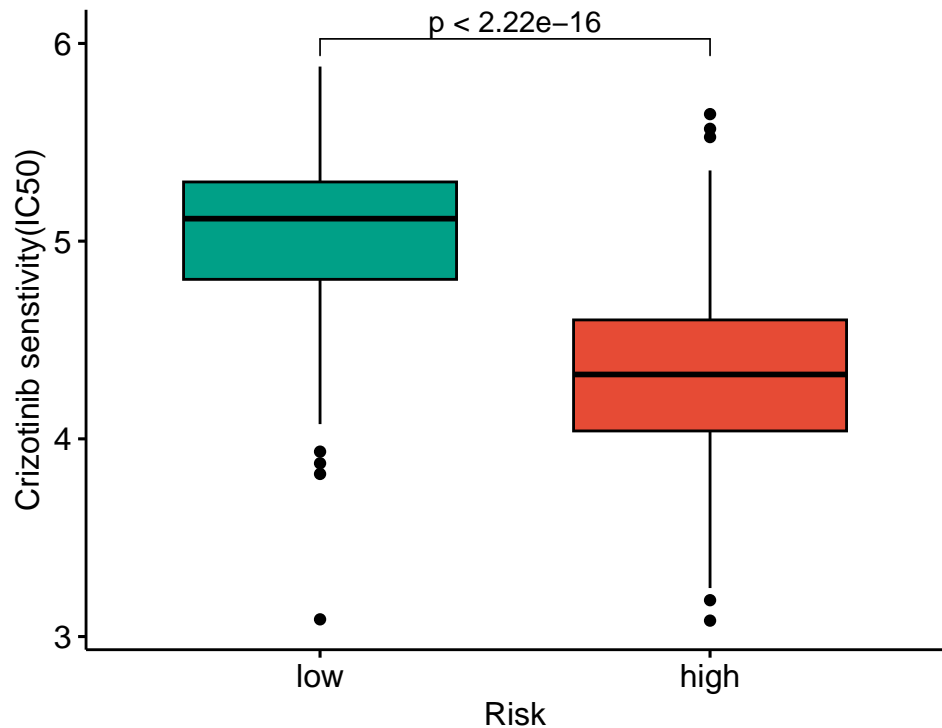

Supplement: Multimedia component 1 [file mmc1.zip › drug/drugSenstivity.Crizotinib.pdf]

Risk 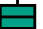 low 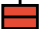 high

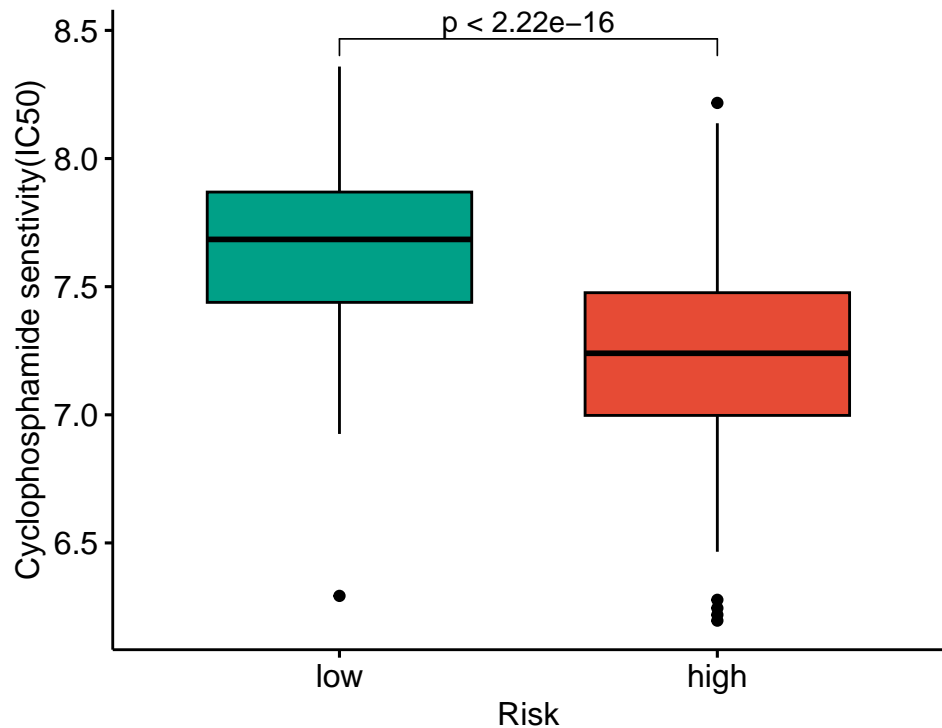

Supplement: Multimedia component 1 [file mmc1.zip › drug/drugSenstivity.Cyclophosphamide.pdf]

Risk 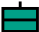 low 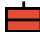 high

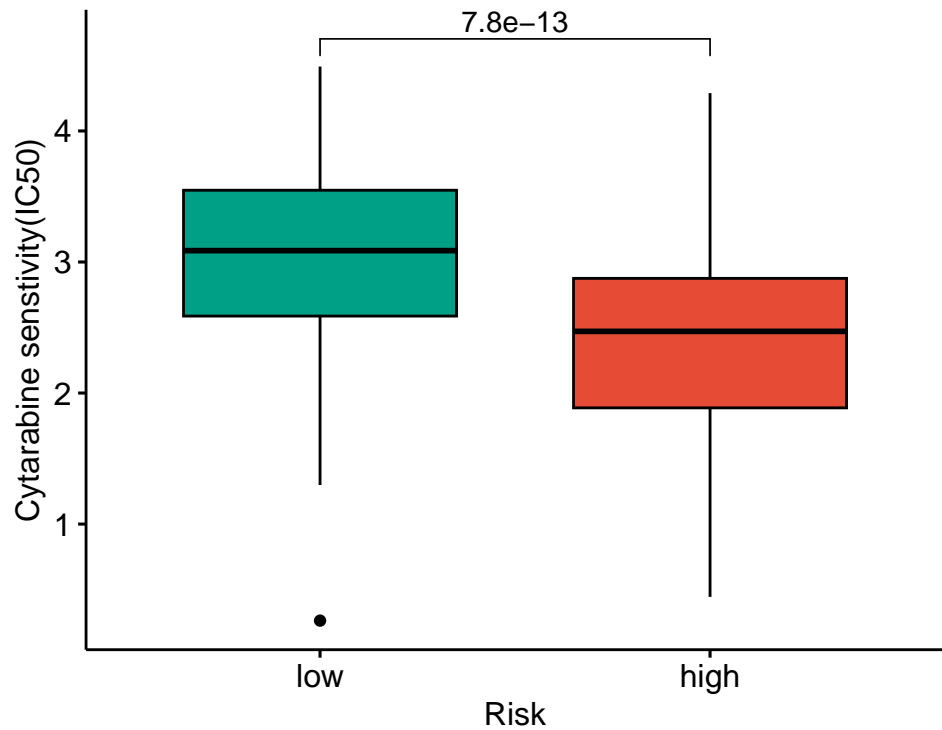

Supplement: Multimedia component 1 [file mmc1.zip › drug/drugSenstivity.Cytarabine.pdf]

Risk 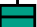 low 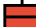 high

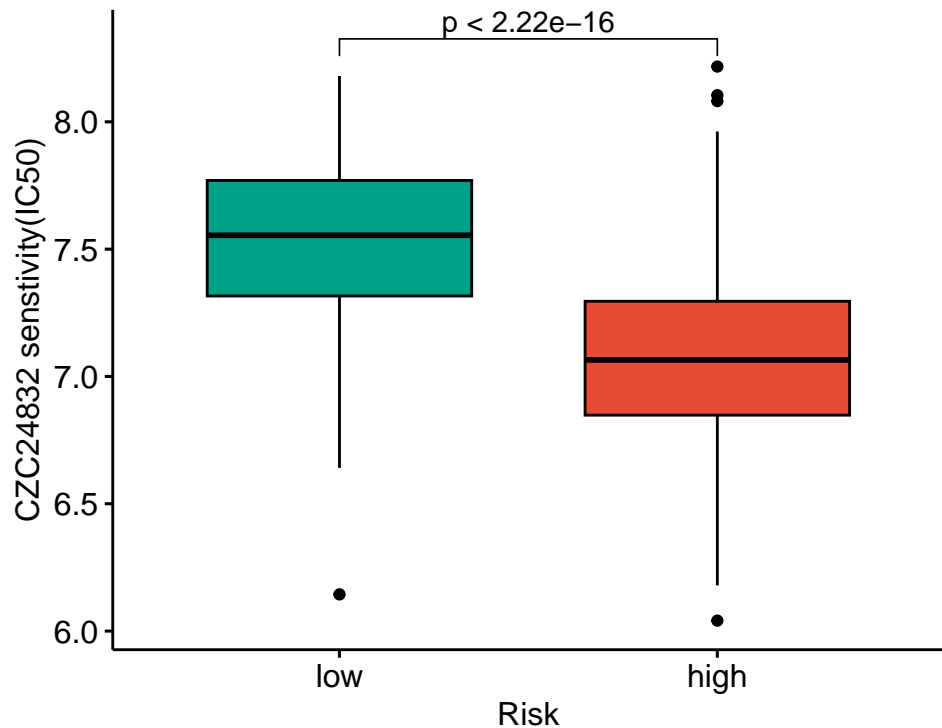

Supplement: Multimedia component 1 [file mmc1.zip › drug/drugSenstivity.CZC24832.pdf]

Risk 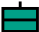 low 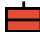 high

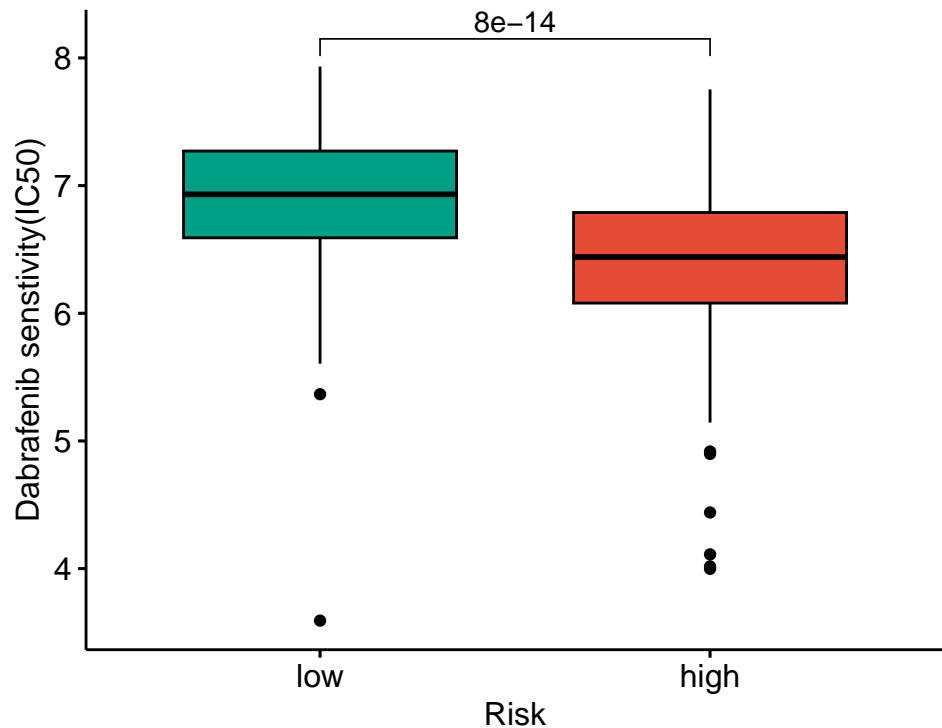

Supplement: Multimedia component 1 [file mmc1.zip › drug/drugSenstivity.Dabrafenib.pdf]

Risk 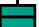 low 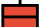 high

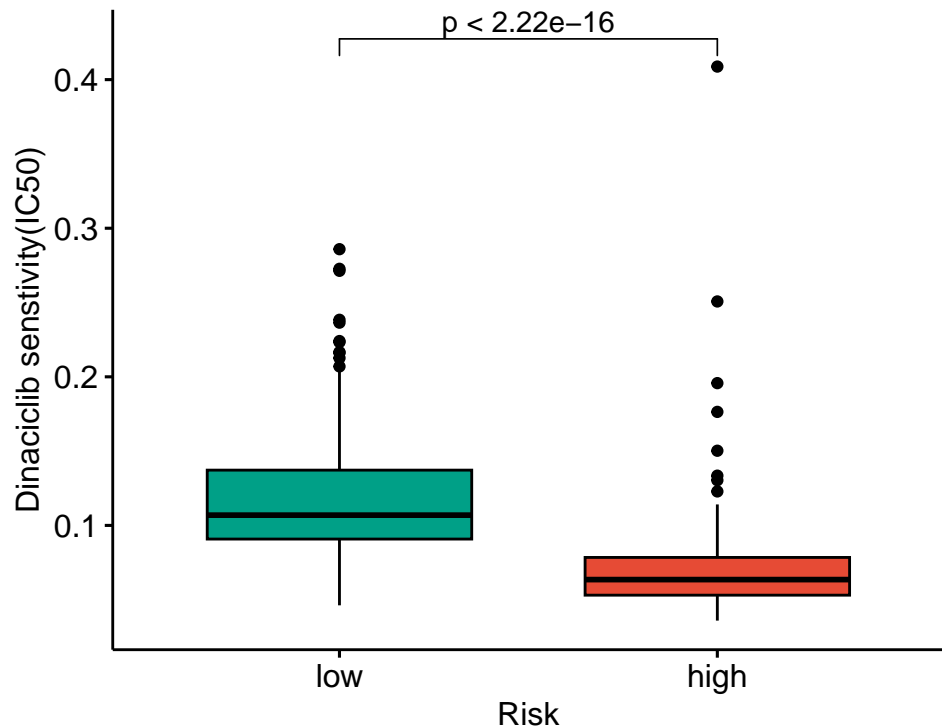

Supplement: Multimedia component 1 [file mmc1.zip › drug/drugSenstivity.Dinaciclib.pdf]

Risk 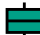 low 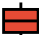 high

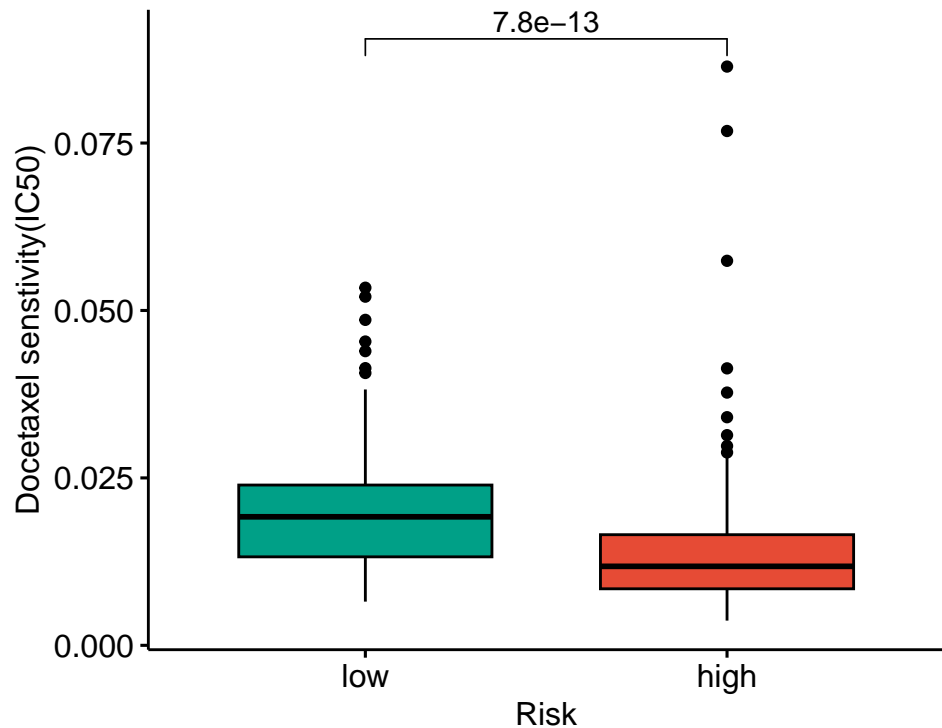

Supplement: Multimedia component 1 [file mmc1.zip › drug/drugSenstivity.Docetaxel.pdf]

Risk 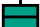 low 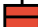 high

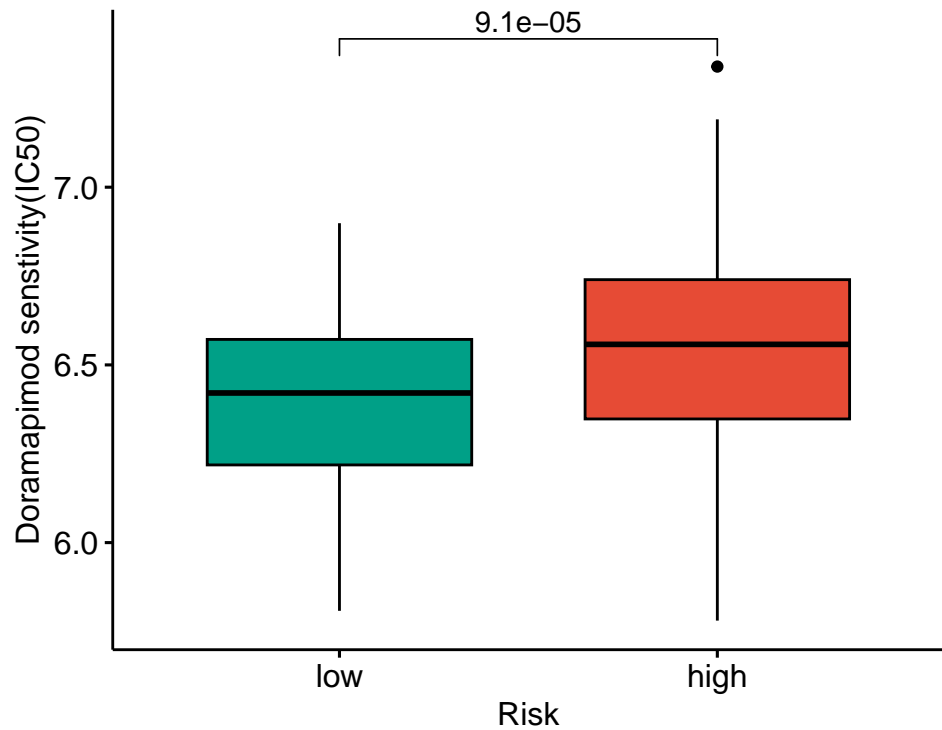

Supplement: Multimedia component 1 [file mmc1.zip › drug/drugSenstivity.Doramapimod.pdf]

Risk 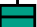 low 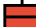 high

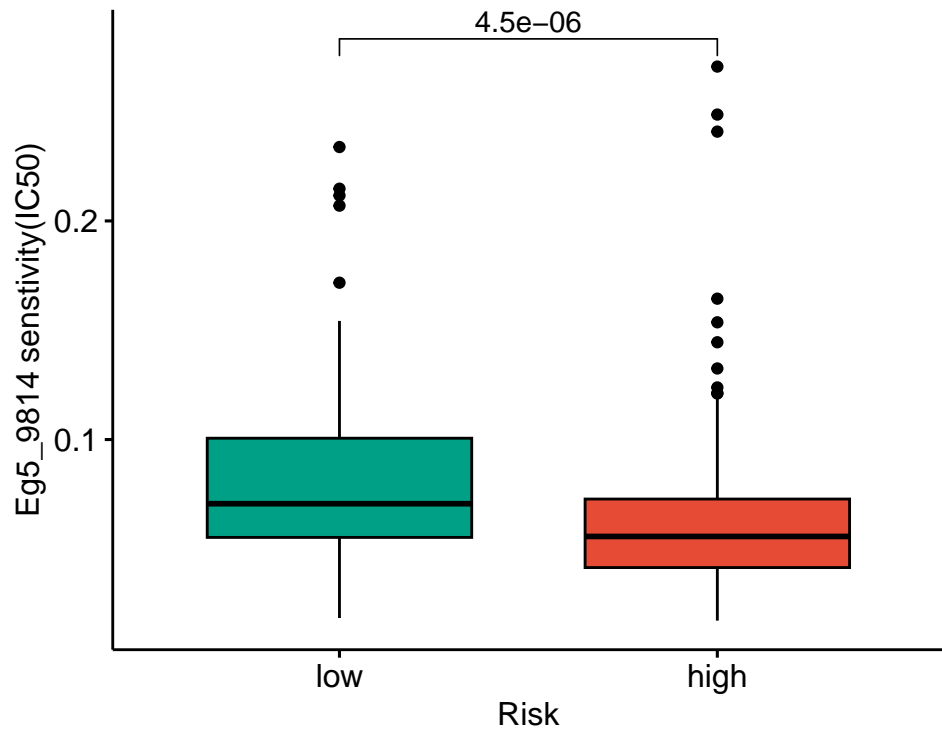

Supplement: Multimedia component 1 [file mmc1.zip › drug/drugSenstivity.Eg5_9814.pdf]

Risk 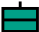 low 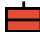 high

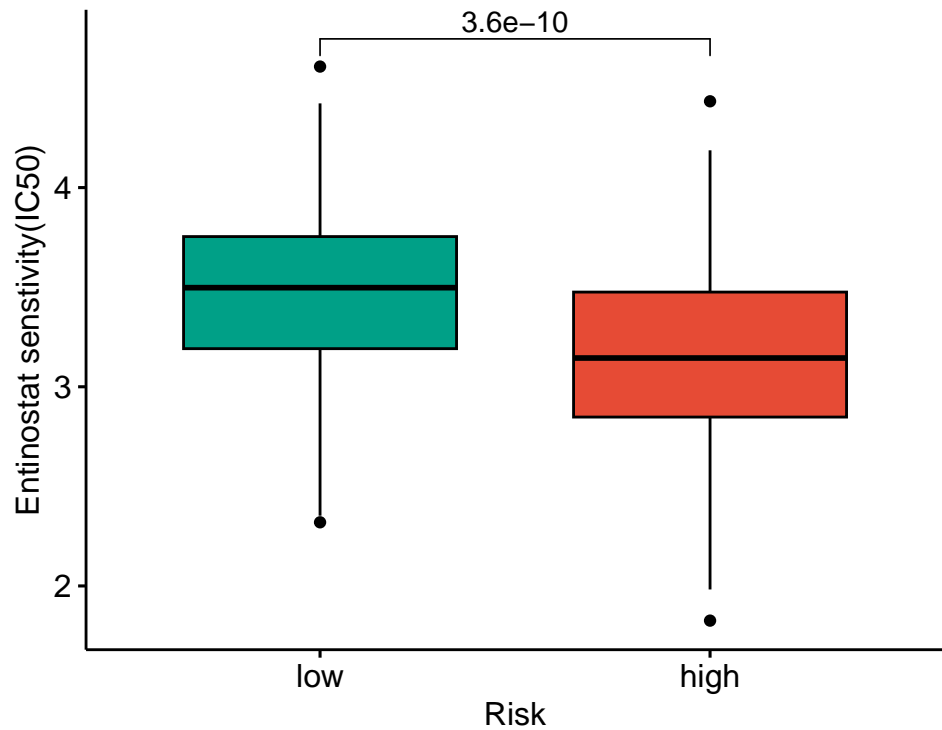

Supplement: Multimedia component 1 [file mmc1.zip › drug/drugSenstivity.Entinostat.pdf]

Risk 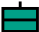 low 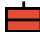 high

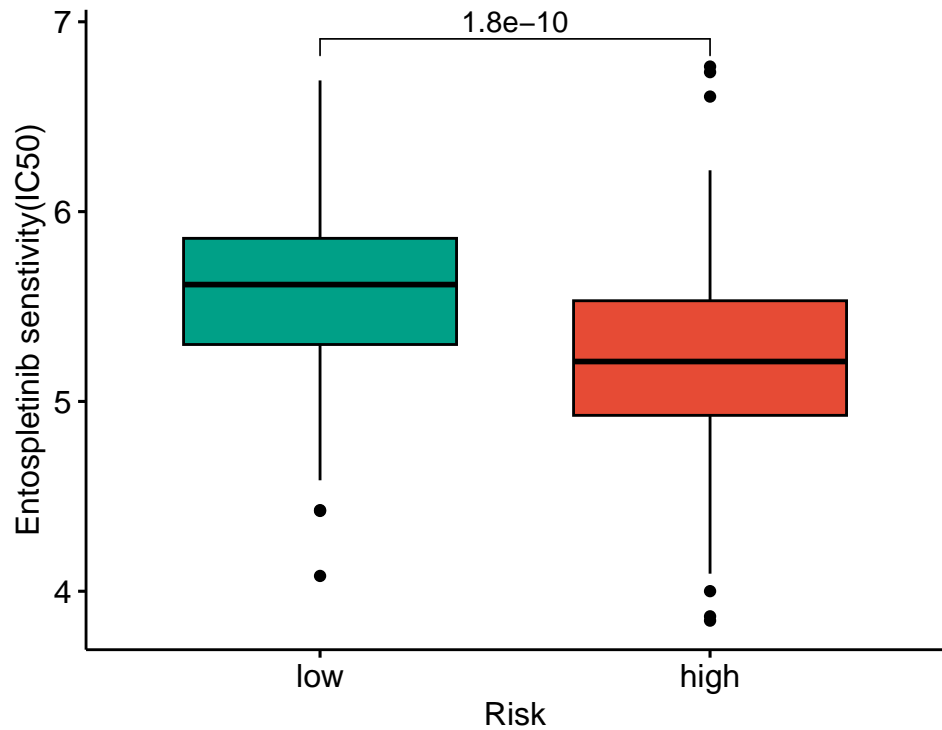

Supplement: Multimedia component 1 [file mmc1.zip › drug/drugSenstivity.Entospletinib.pdf]

Risk 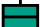 low 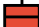 high

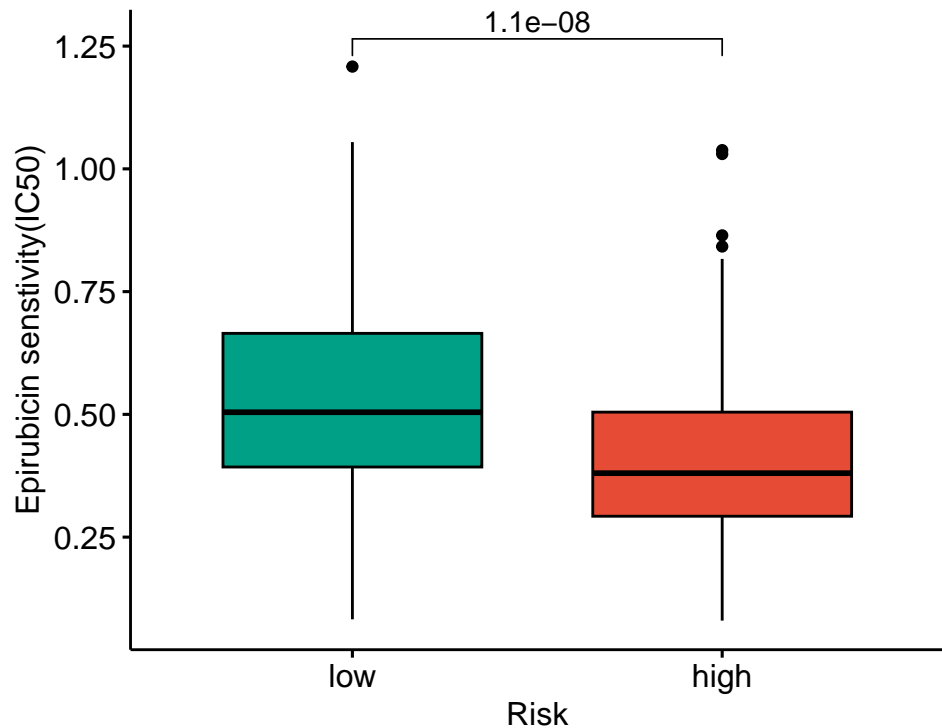

Supplement: Multimedia component 1 [file mmc1.zip › drug/drugSenstivity.Epirubicin.pdf]

Risk 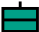 low 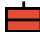 high

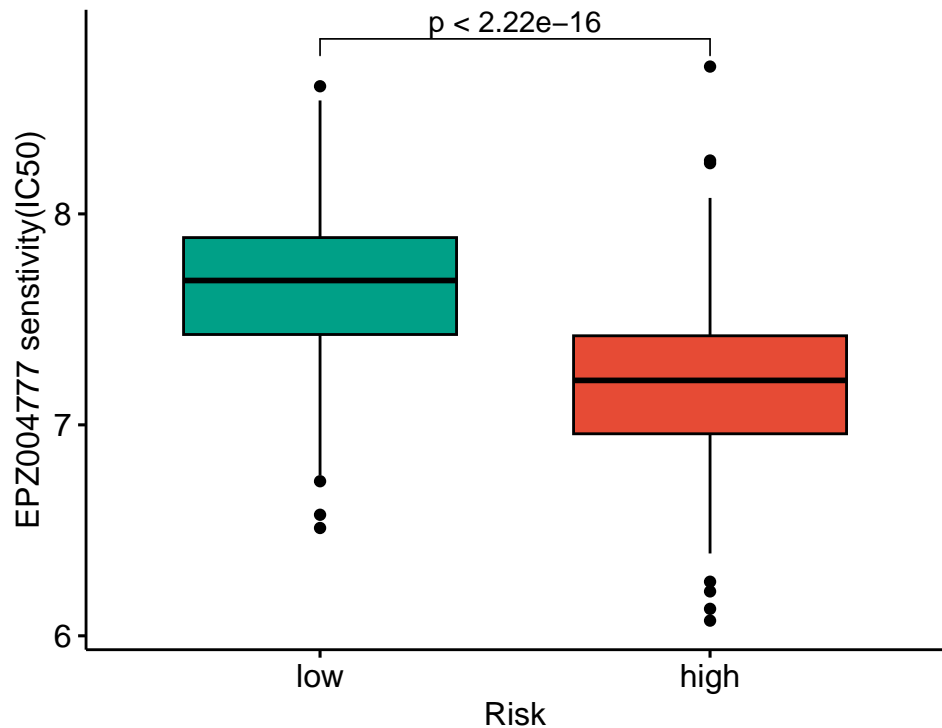

Supplement: Multimedia component 1 [file mmc1.zip › drug/drugSenstivity.EPZ004777.pdf]

Risk 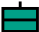 low 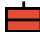 high

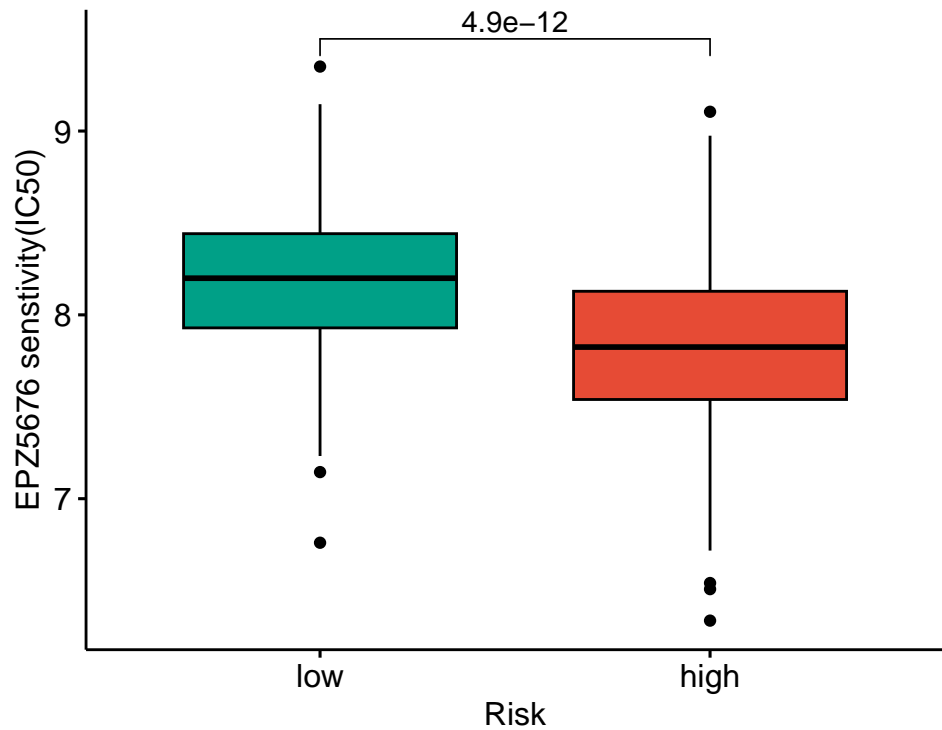

Supplement: Multimedia component 1 [file mmc1.zip › drug/drugSenstivity.EPZ5676.pdf]

Risk 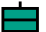 low 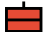 high

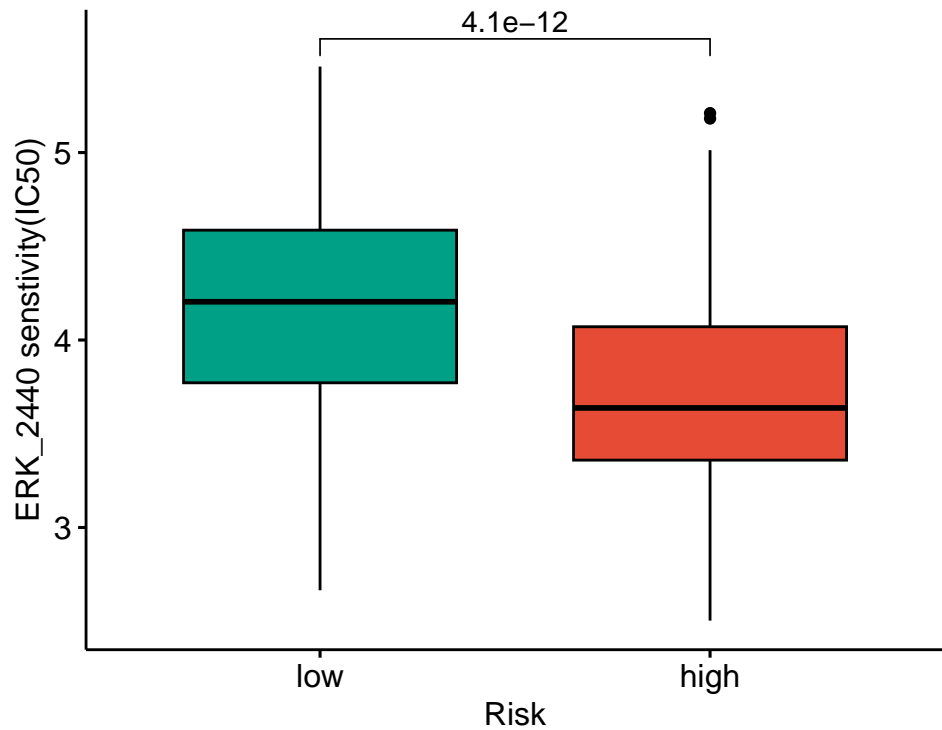

Supplement: Multimedia component 1 [file mmc1.zip › drug/drugSenstivity.ERK_2440.pdf]

Risk 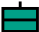 low 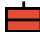 high

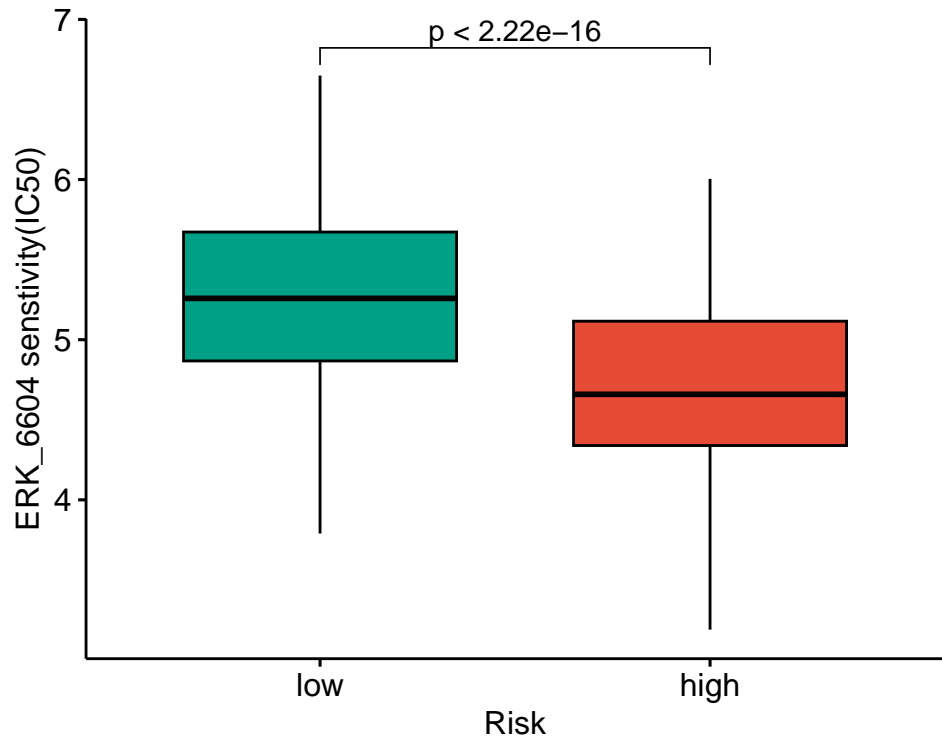

Supplement: Multimedia component 1 [file mmc1.zip › drug/drugSenstivity.ERK_6604.pdf]

Risk 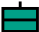 low 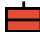 high

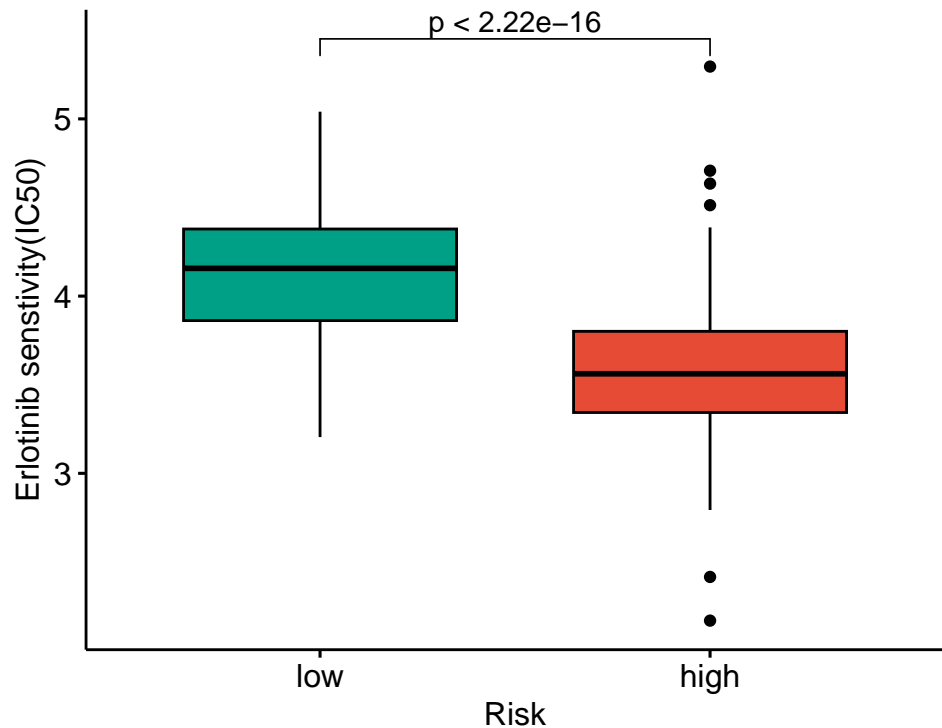

Supplement: Multimedia component 1 [file mmc1.zip › drug/drugSenstivity.Erlotinib.pdf]

Risk 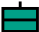 low 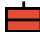 high

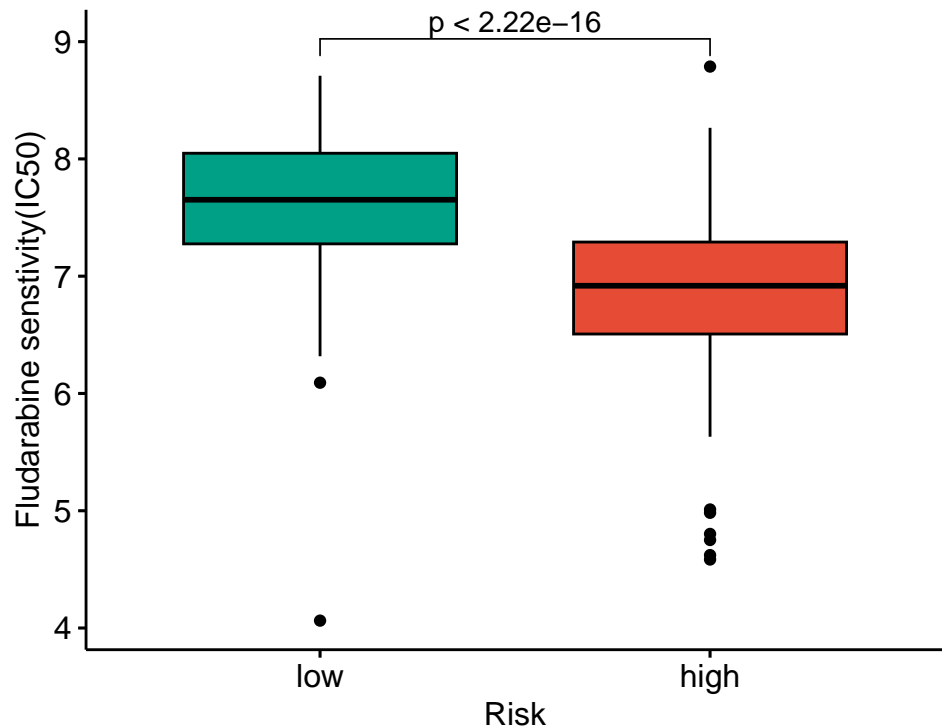

Supplement: Multimedia component 1 [file mmc1.zip › drug/drugSenstivity.Fludarabine.pdf]

Risk 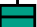 low 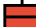 high

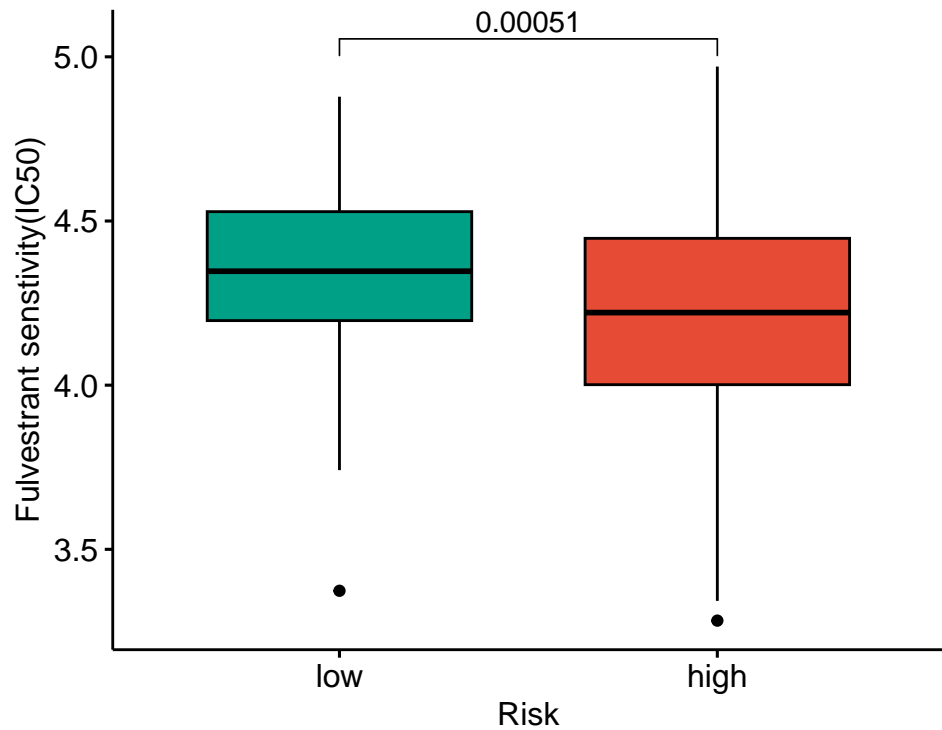

Supplement: Multimedia component 1 [file mmc1.zip › drug/drugSenstivity.Fulvestrant.pdf]

Risk 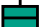 low 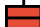 high

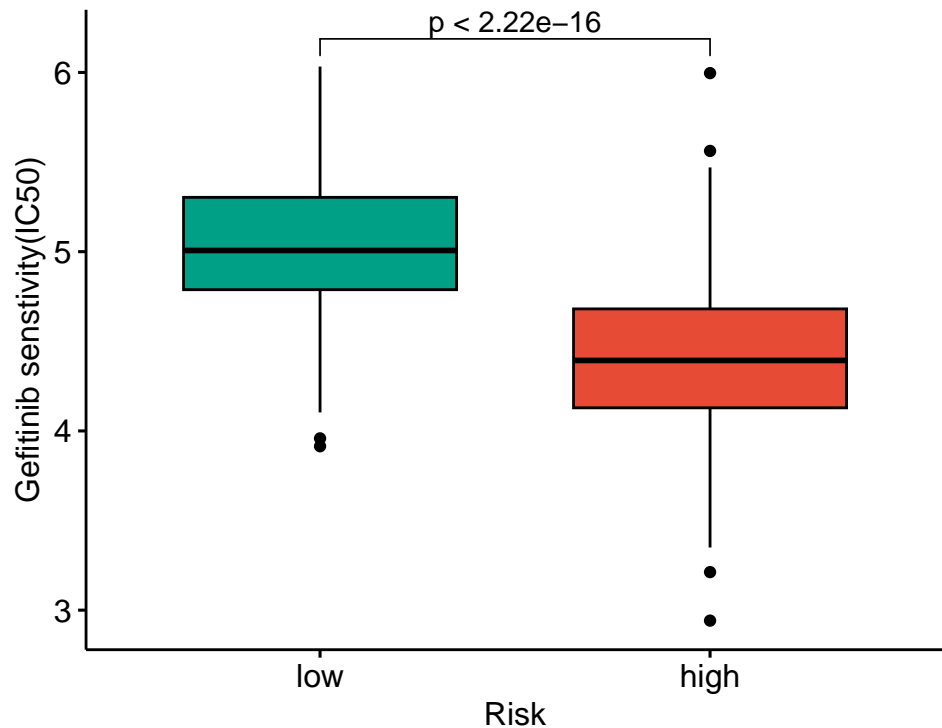

Supplement: Multimedia component 1 [file mmc1.zip › drug/drugSenstivity.Gefitinib.pdf]

Risk 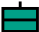 low 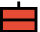 high

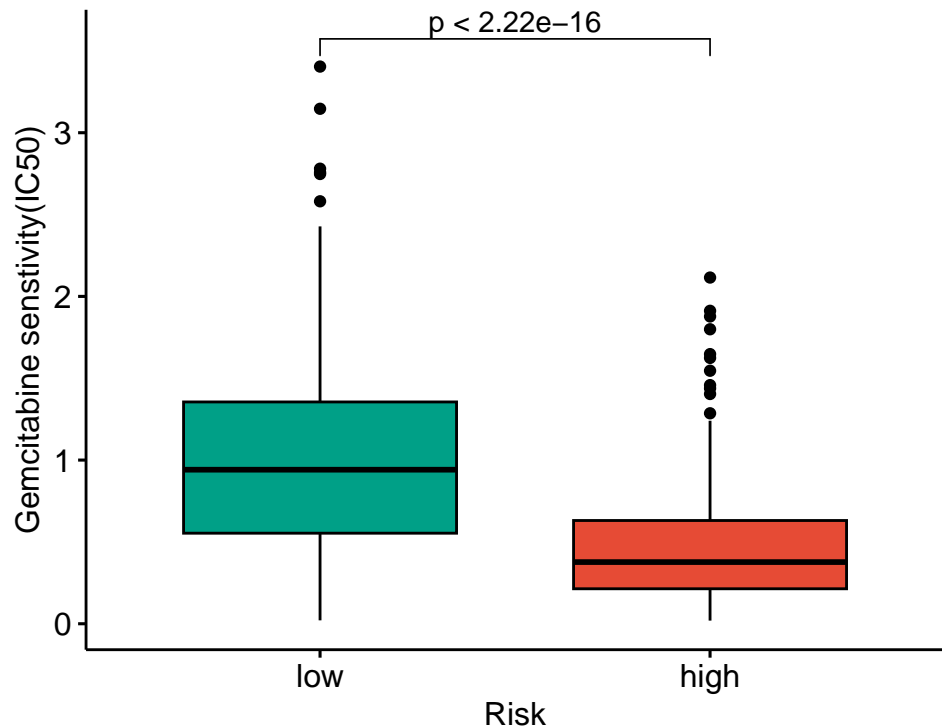

Supplement: Multimedia component 1 [file mmc1.zip › drug/drugSenstivity.Gemcitabine.pdf]

Risk 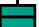 low 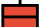 high

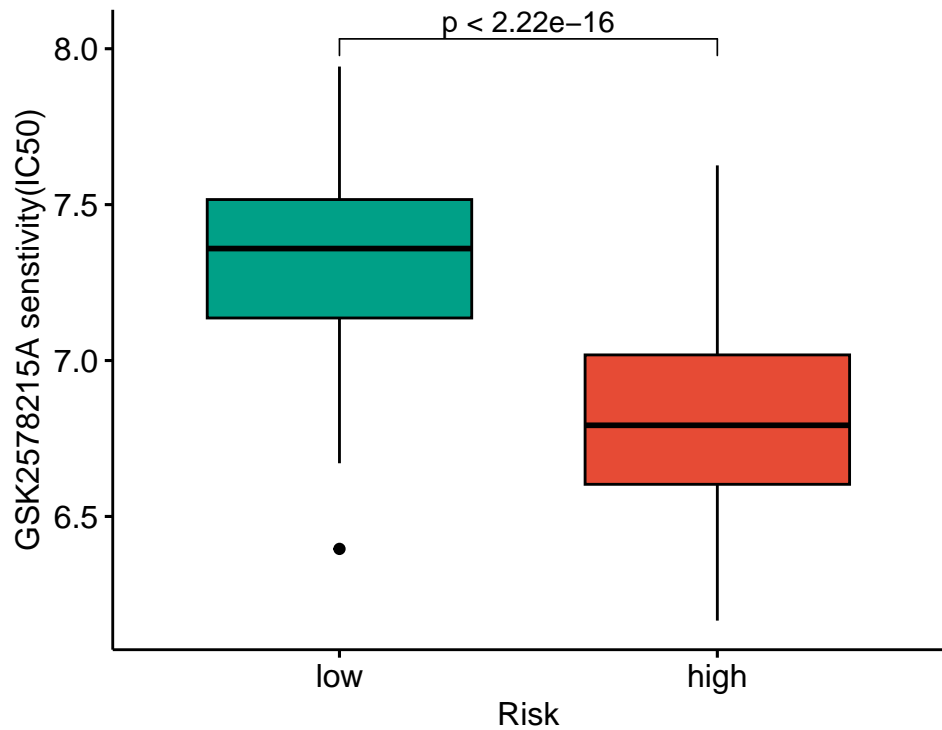

Supplement: Multimedia component 1 [file mmc1.zip › drug/drugSenstivity.GSK2578215A.pdf]

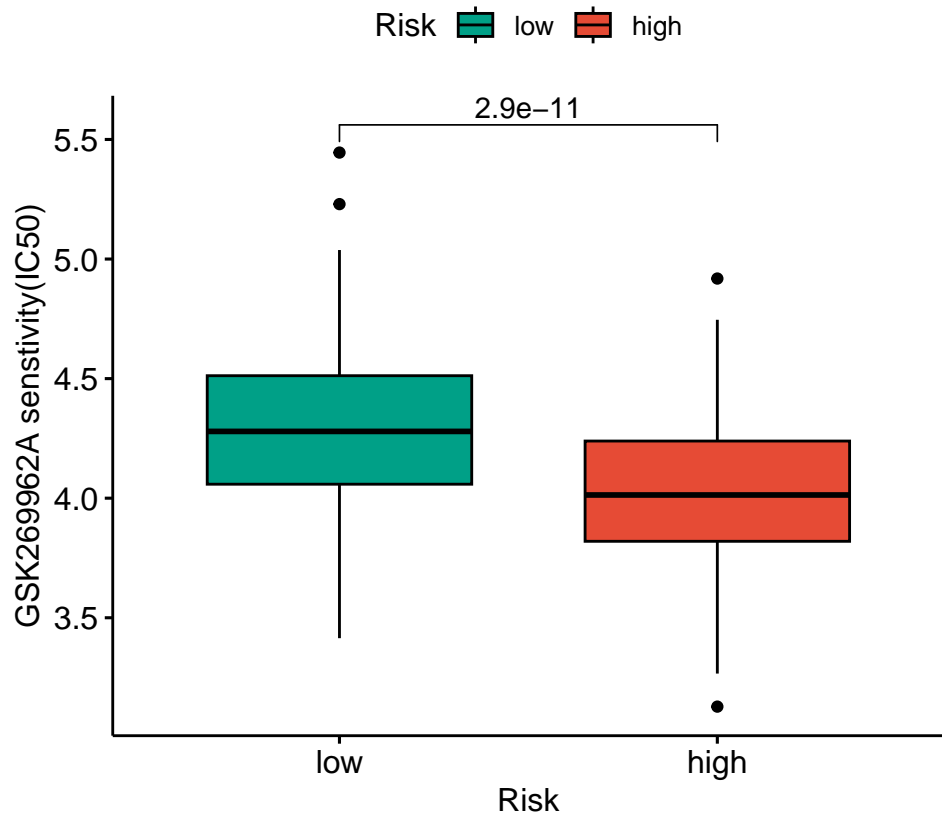

Supplement: Multimedia component 1 [file mmc1.zip › drug/drugSenstivity.GSK269962A.pdf]

Risk 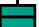 low 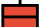 high

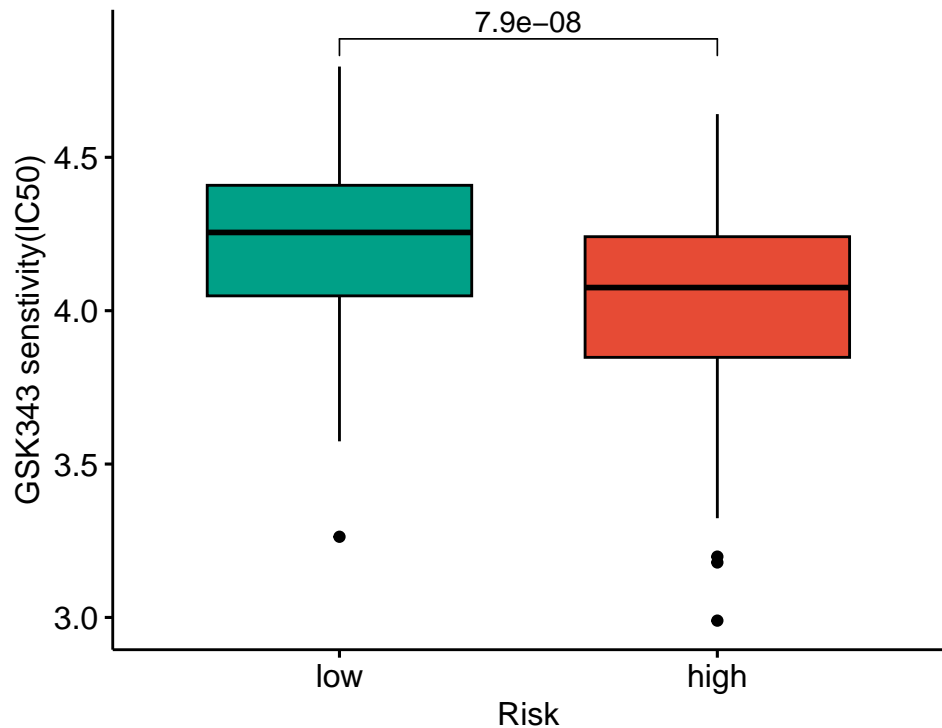

Supplement: Multimedia component 1 [file mmc1.zip › drug/drugSenstivity.GSK343.pdf]

Risk 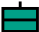 low 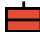 high

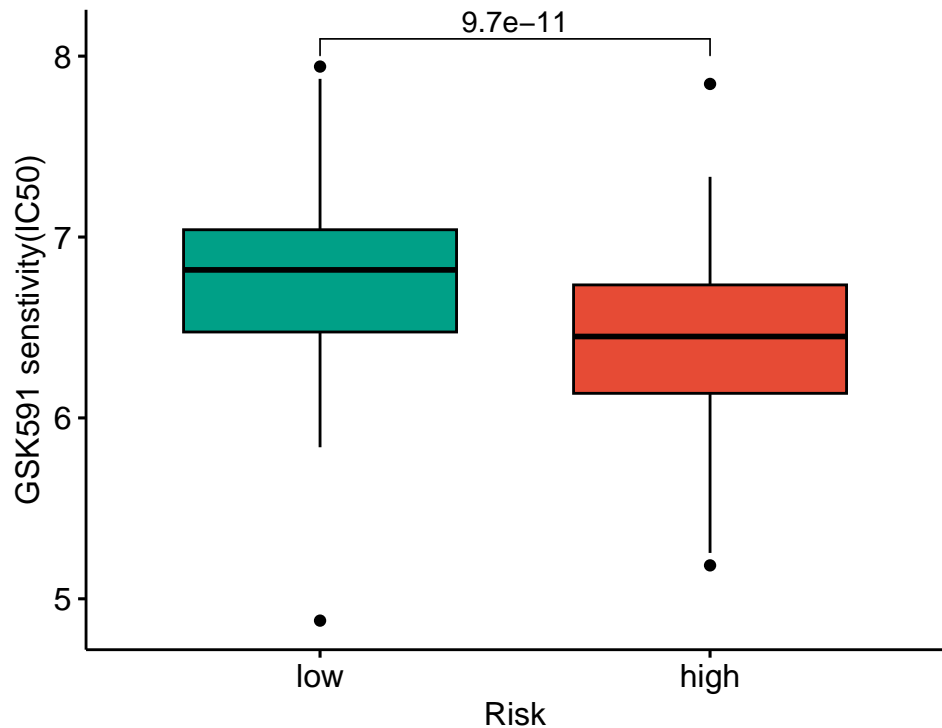

Supplement: Multimedia component 1 [file mmc1.zip › drug/drugSenstivity.GSK591.pdf]

Risk 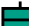 low 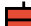 high

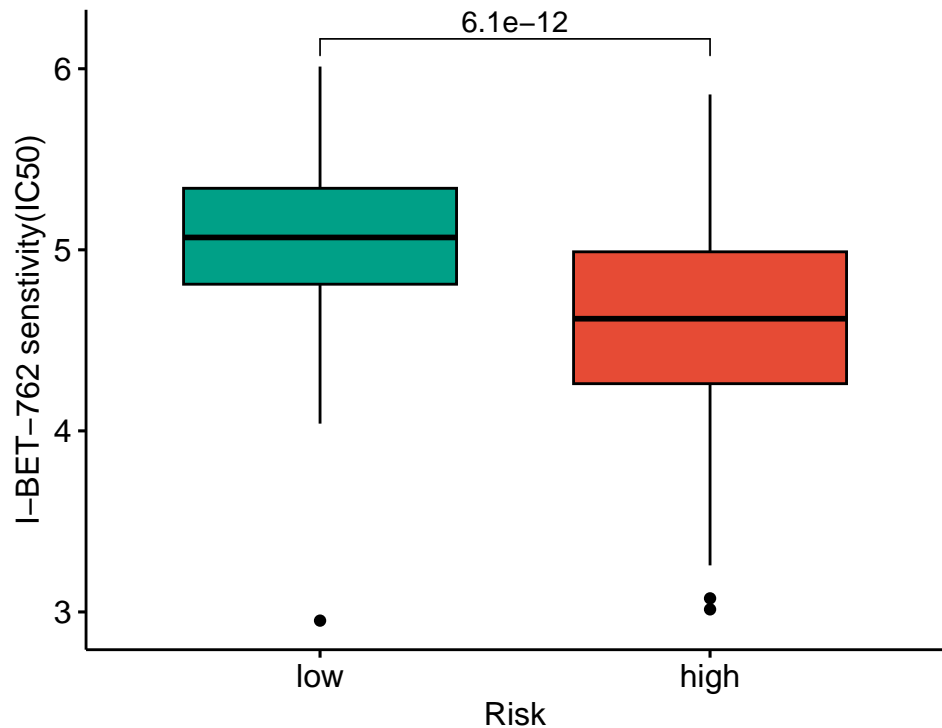

Supplement: Multimedia component 1 [file mmc1.zip › drug/drugSenstivity.I-BET-762.pdf]

Risk 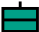 low 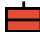 high

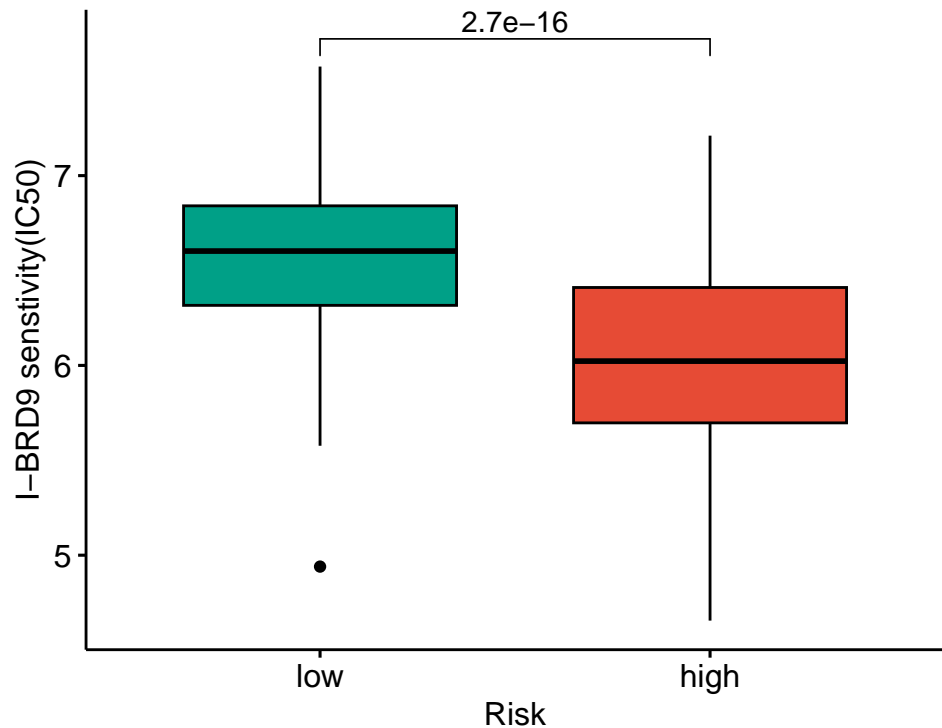

Supplement: Multimedia component 1 [file mmc1.zip › drug/drugSenstivity.I-BRD9.pdf]

Risk 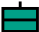 low 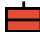 high

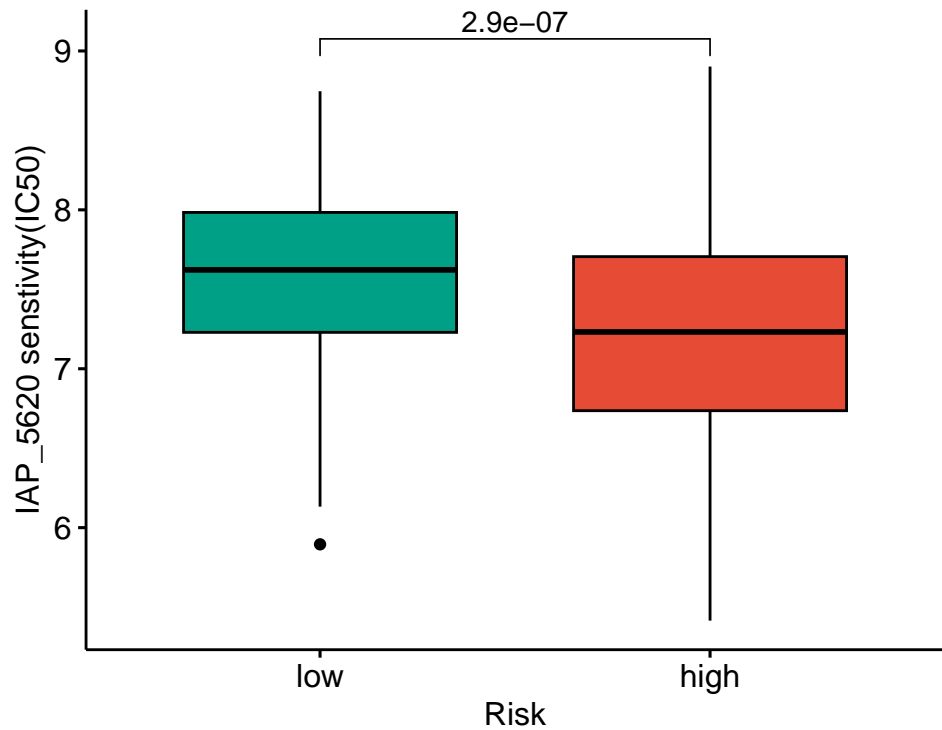

Supplement: Multimedia component 1 [file mmc1.zip › drug/drugSenstivity.IAP_5620.pdf]

Risk 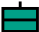 low 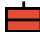 high

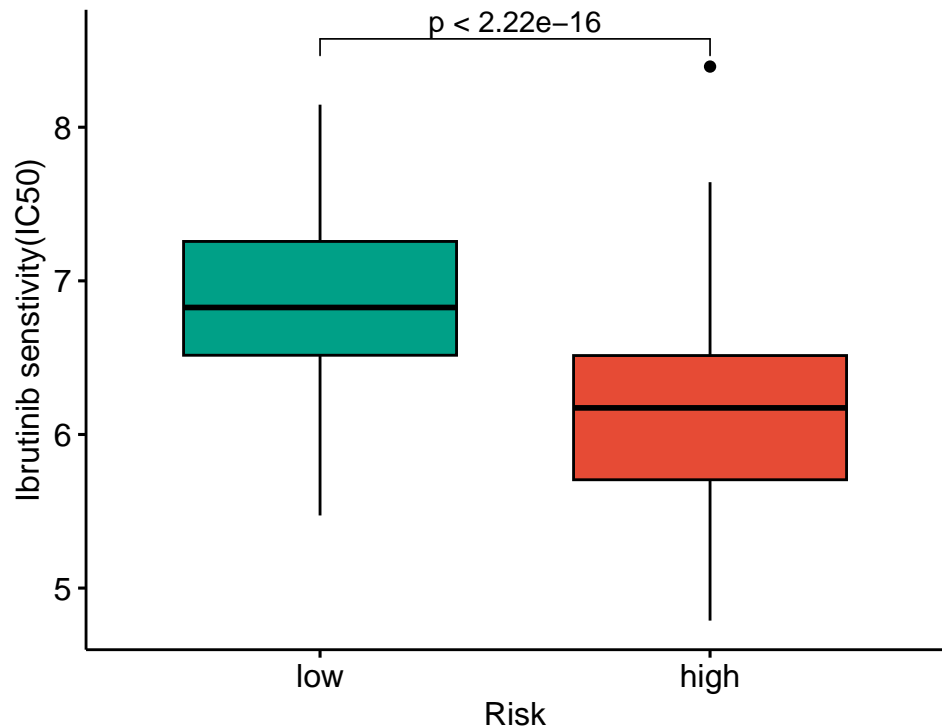

Supplement: Multimedia component 1 [file mmc1.zip › drug/drugSenstivity.Ibrutinib.pdf]

Risk 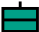 low 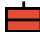 high

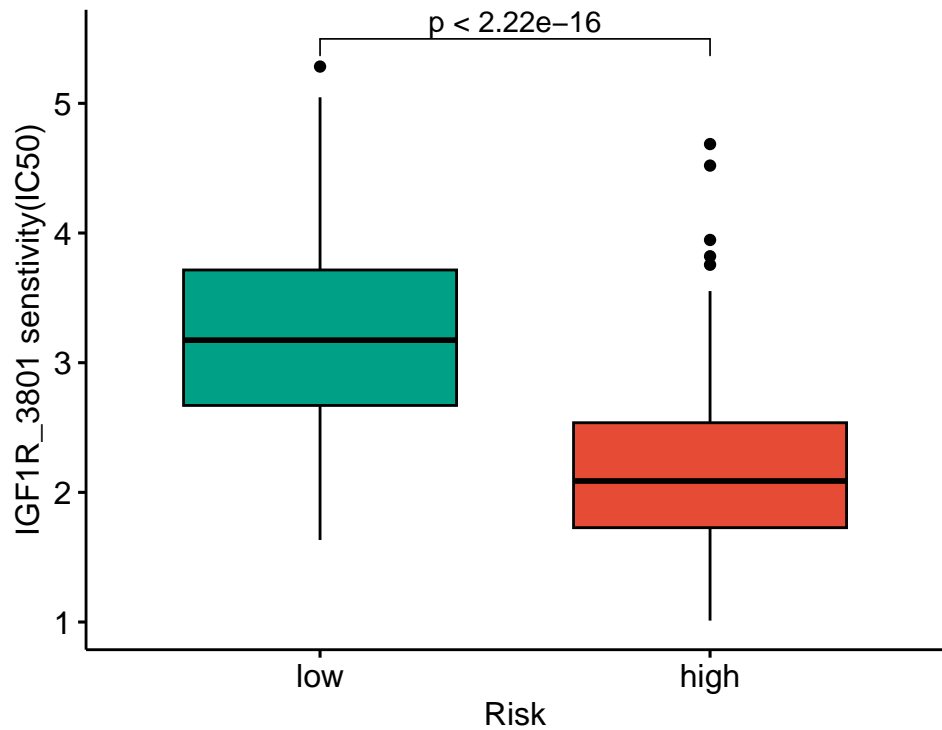

Supplement: Multimedia component 1 [file mmc1.zip › drug/drugSenstivity.IGF1R_3801.pdf]

Risk 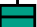 low 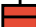 high

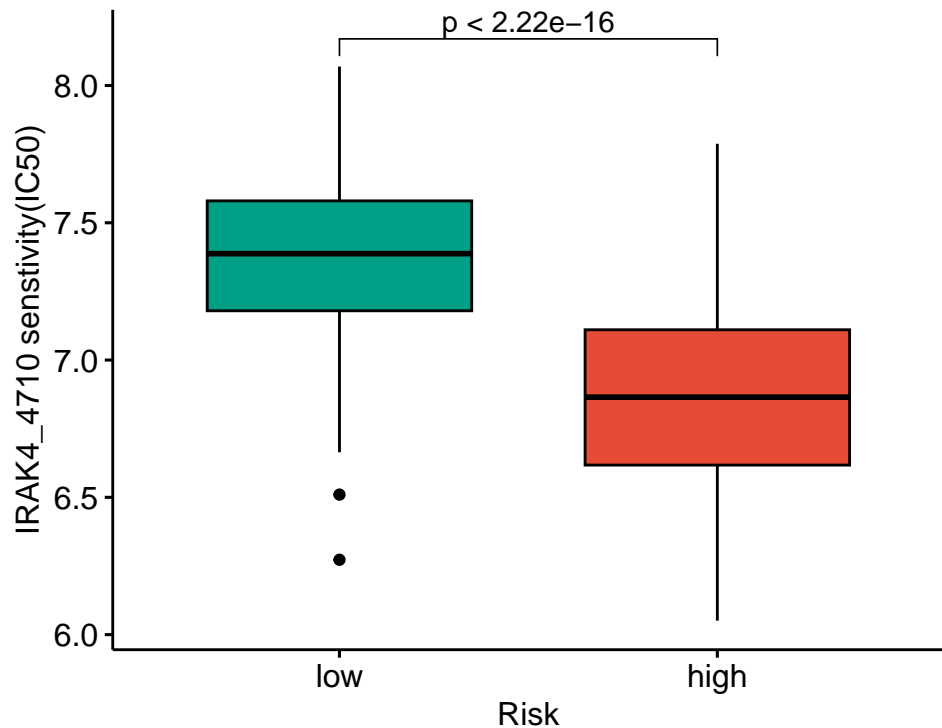

Supplement: Multimedia component 1 [file mmc1.zip › drug/drugSenstivity.IRAK4_4710.pdf]

Risk 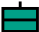 low 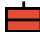 high

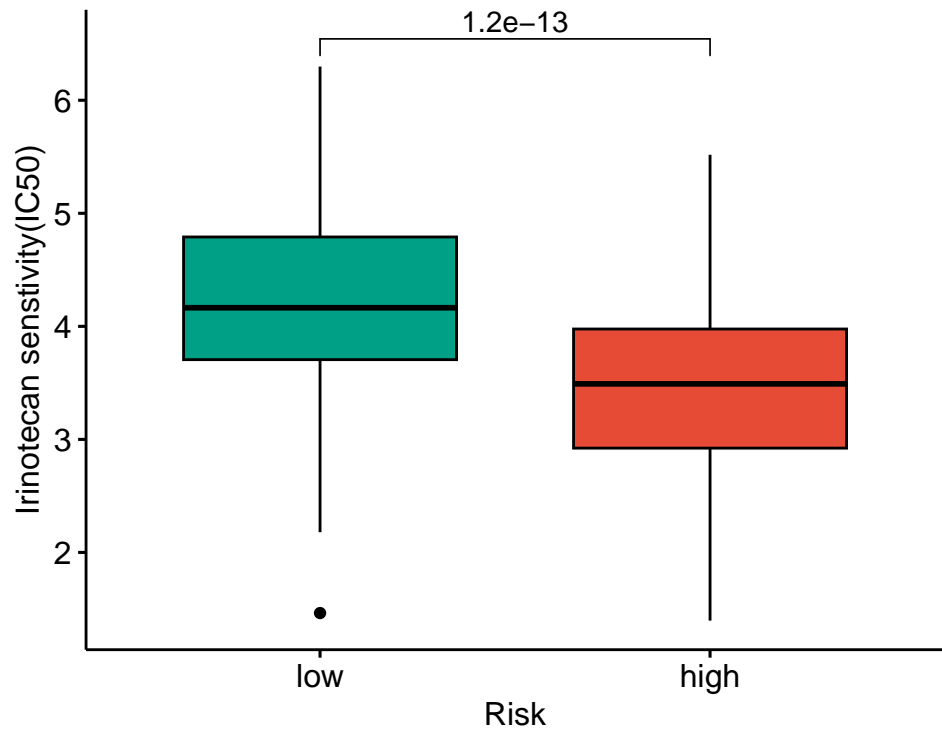

Supplement: Multimedia component 1 [file mmc1.zip › drug/drugSenstivity.Irinotecan.pdf]

Risk 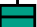 low 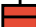 high

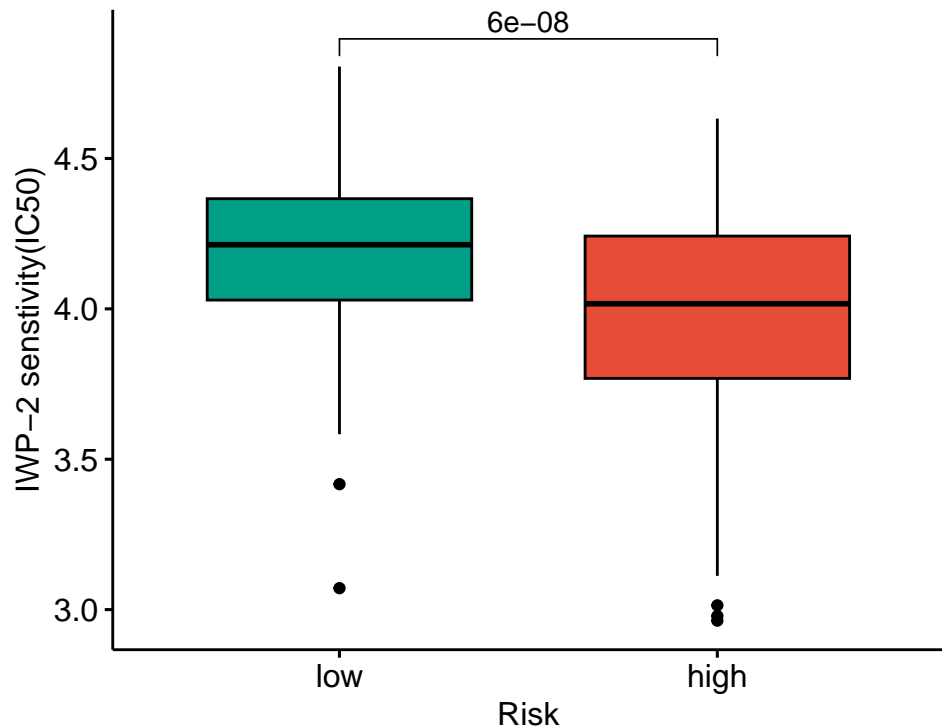

Supplement: Multimedia component 1 [file mmc1.zip › drug/drugSenstivity.IWP-2.pdf]

Risk 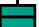 low 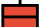 high

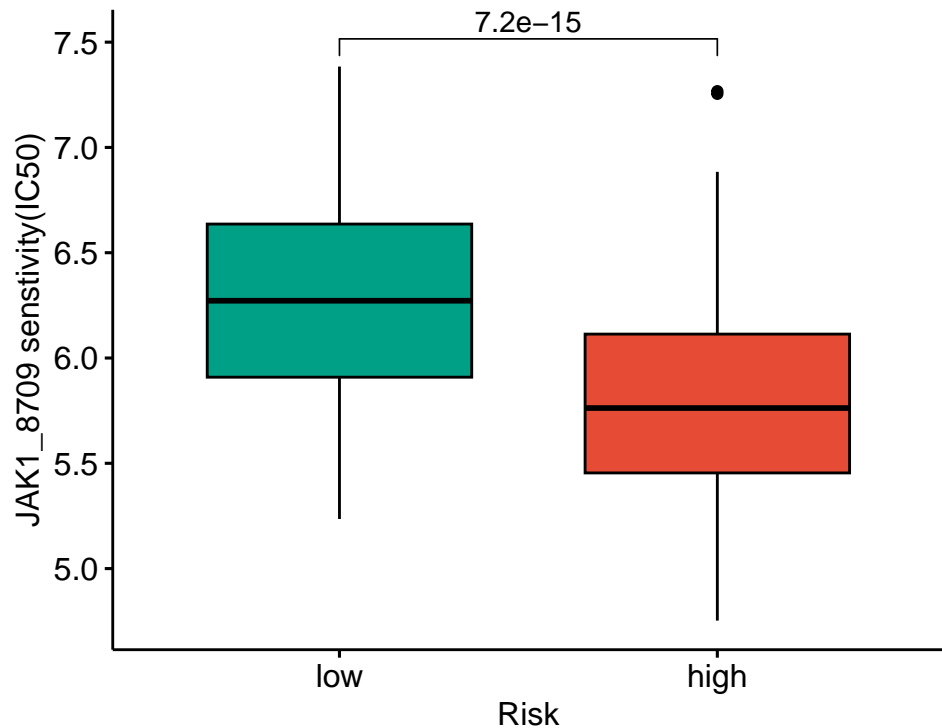

Supplement: Multimedia component 1 [file mmc1.zip › drug/drugSenstivity.JAK1_8709.pdf]

Risk 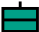 low 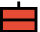 high

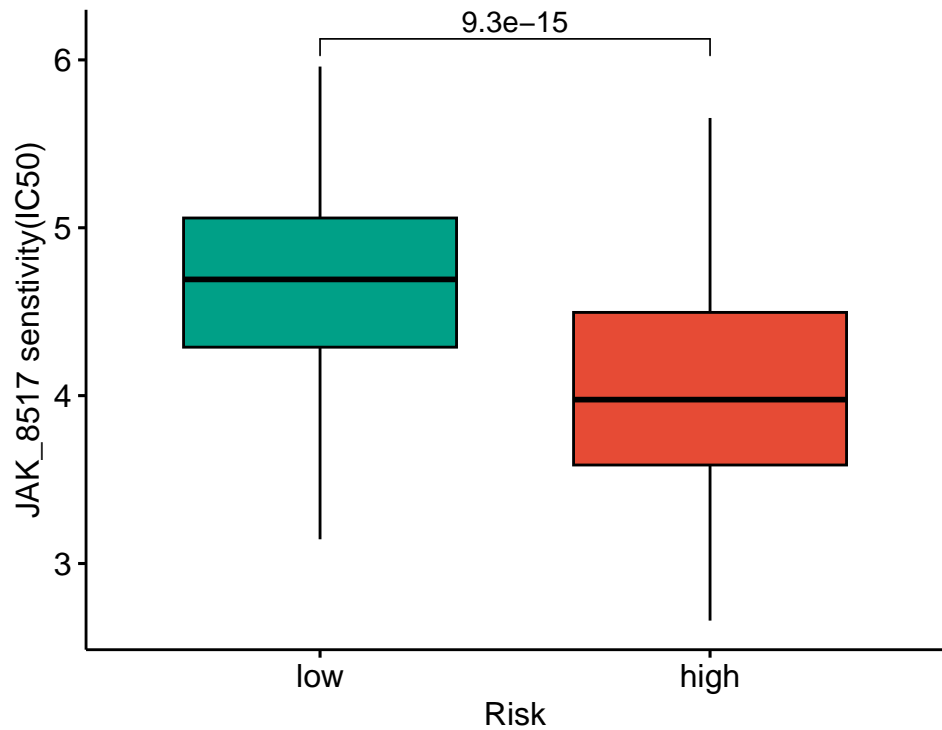

Supplement: Multimedia component 1 [file mmc1.zip › drug/drugSenstivity.JAK_8517.pdf]

Risk 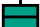 low 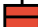 high

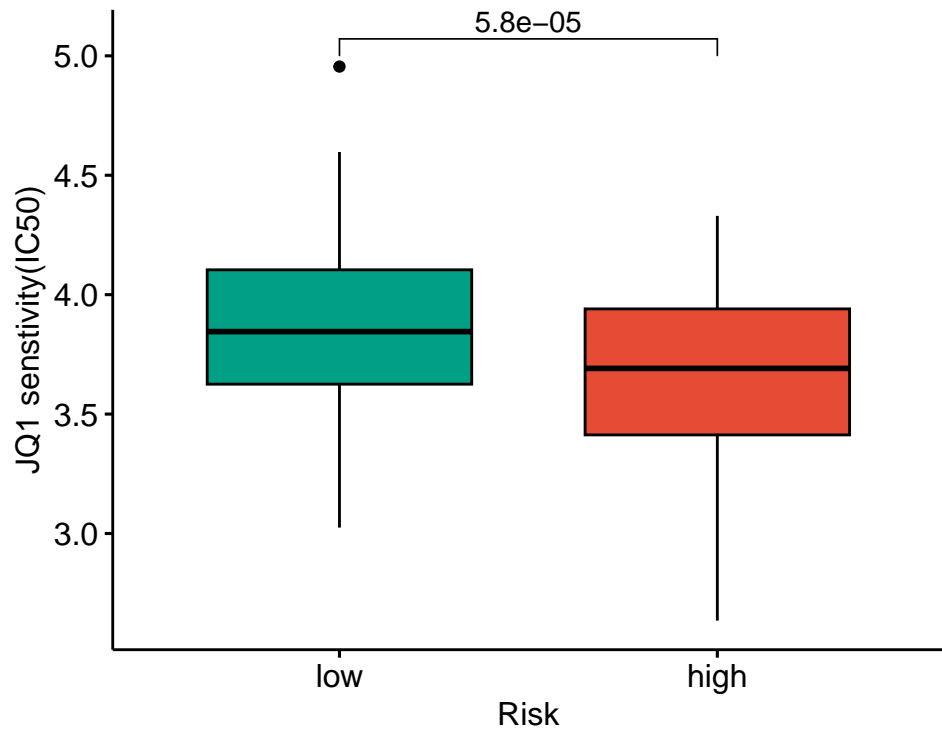

Supplement: Multimedia component 1 [file mmc1.zip › drug/drugSenstivity.JQ1.pdf]

Risk 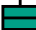 low 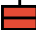 high

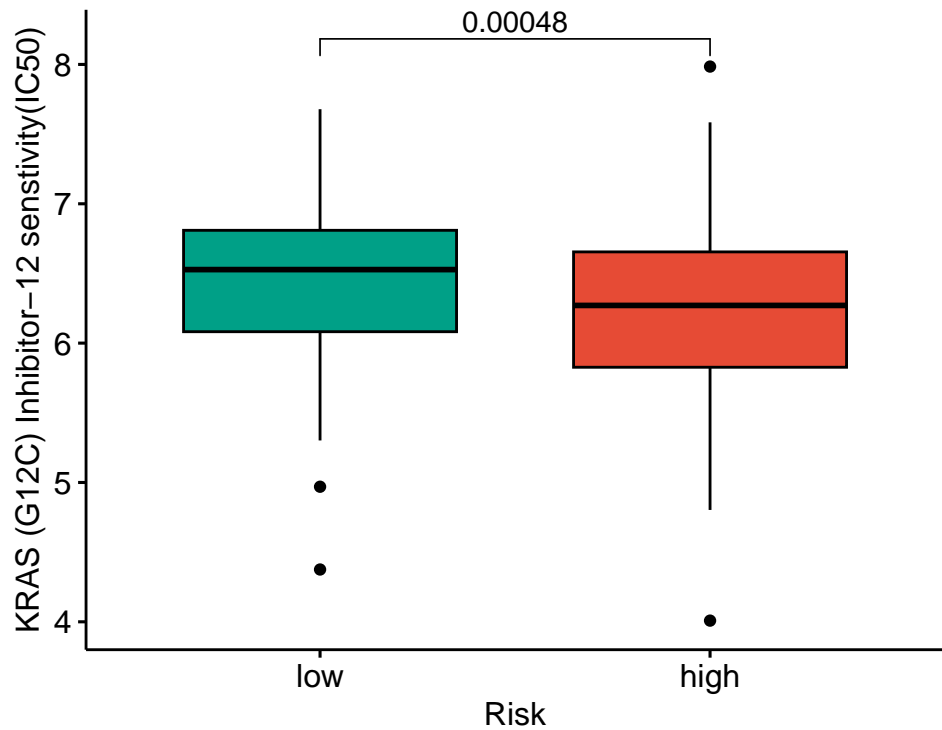

Supplement: Multimedia component 1 [file mmc1.zip › drug/drugSenstivity.KRAS (G12C) Inhibitor-12.pdf]

Risk 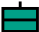 low 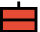 high

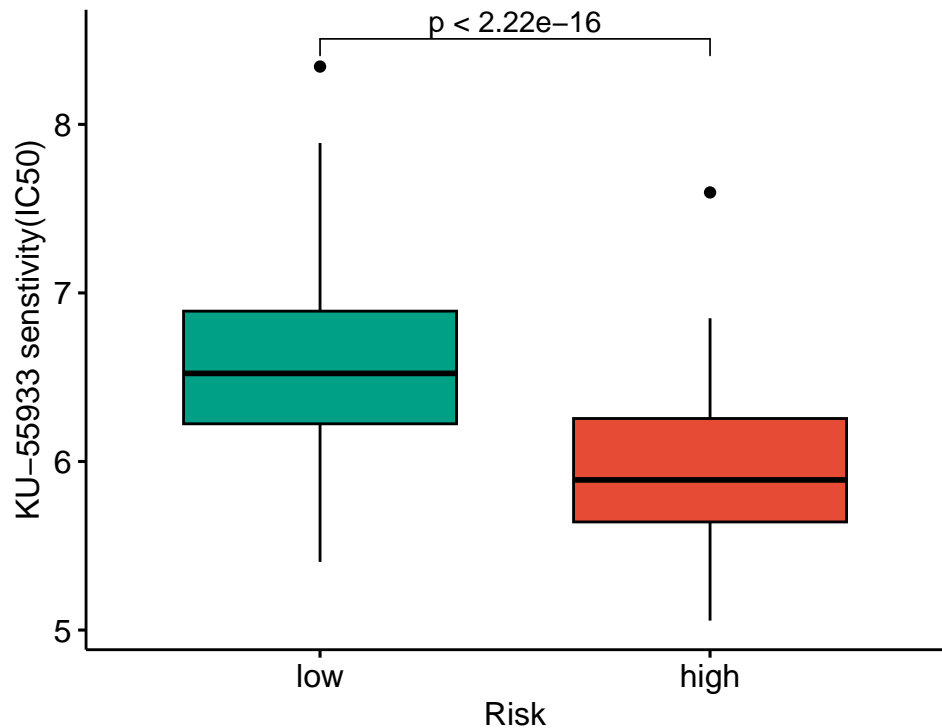

Supplement: Multimedia component 1 [file mmc1.zip › drug/drugSenstivity.KU-55933.pdf]

Risk 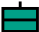 low 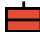 high

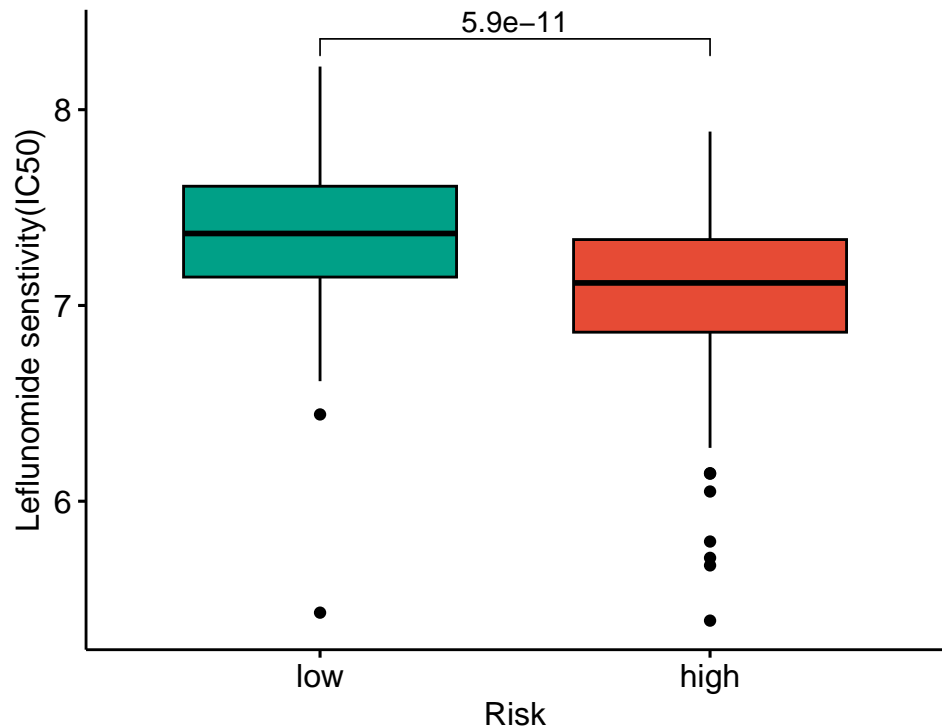

Supplement: Multimedia component 1 [file mmc1.zip › drug/drugSenstivity.Leflunomide.pdf]

Risk 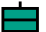 low 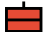 high

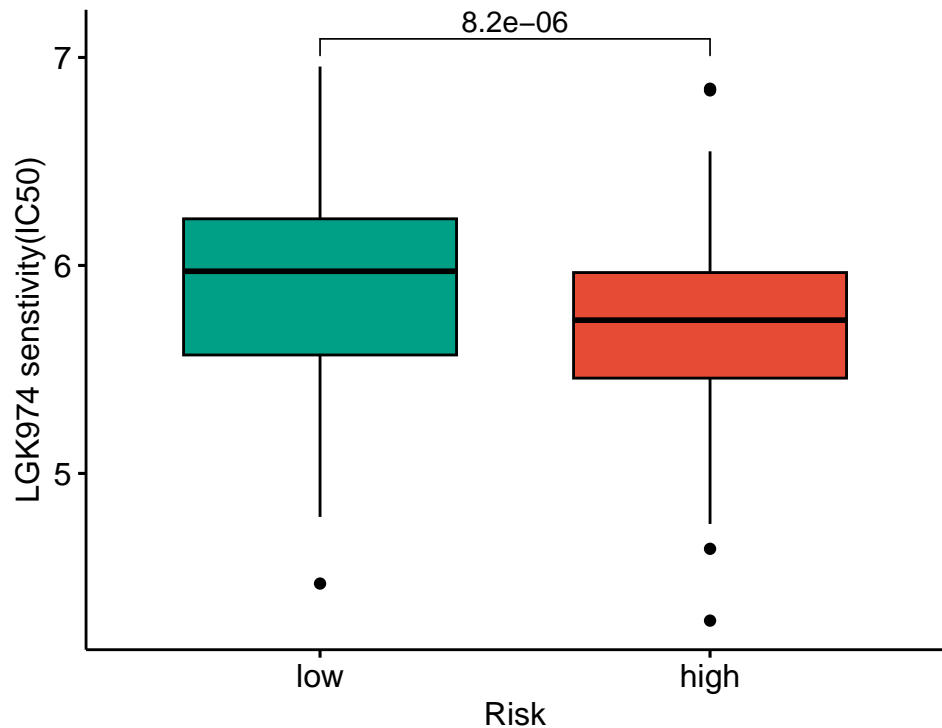

Supplement: Multimedia component 1 [file mmc1.zip › drug/drugSenstivity.LGK974.pdf]

Risk 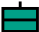 low 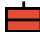 high

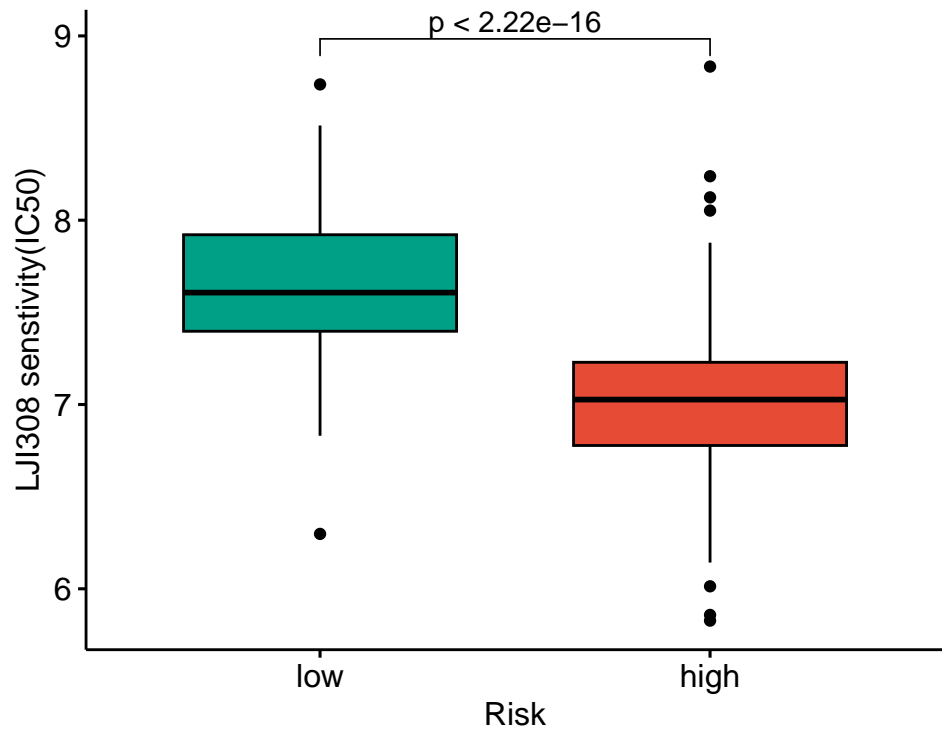

Supplement: Multimedia component 1 [file mmc1.zip › drug/drugSenstivity.LJI308.pdf]

Risk 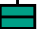 low 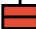 high

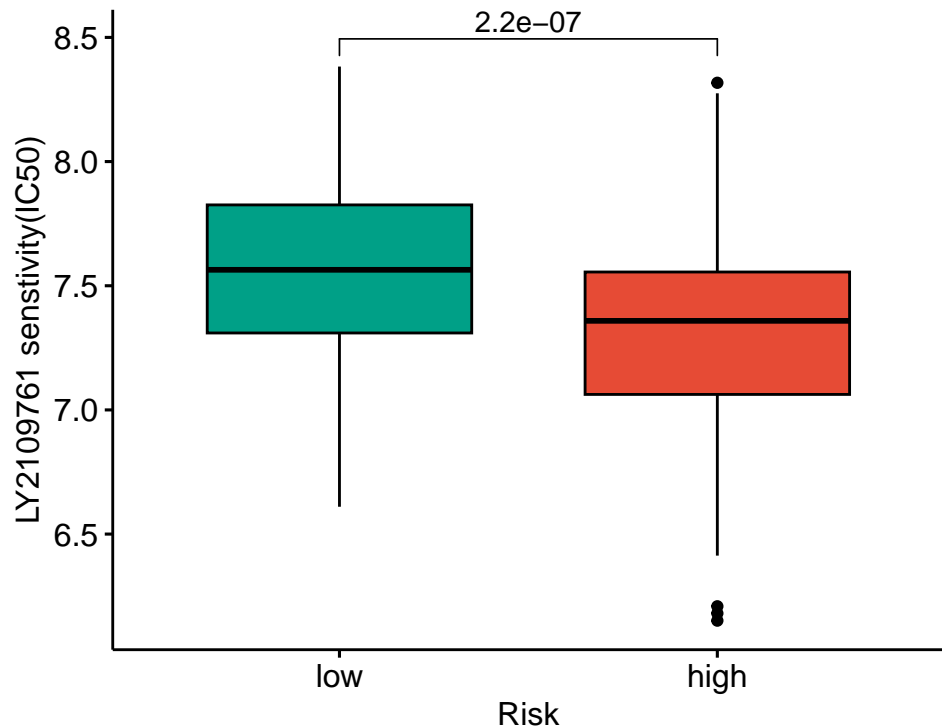

Supplement: Multimedia component 1 [file mmc1.zip › drug/drugSenstivity.LY2109761.pdf]

Risk 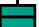 low 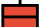 high

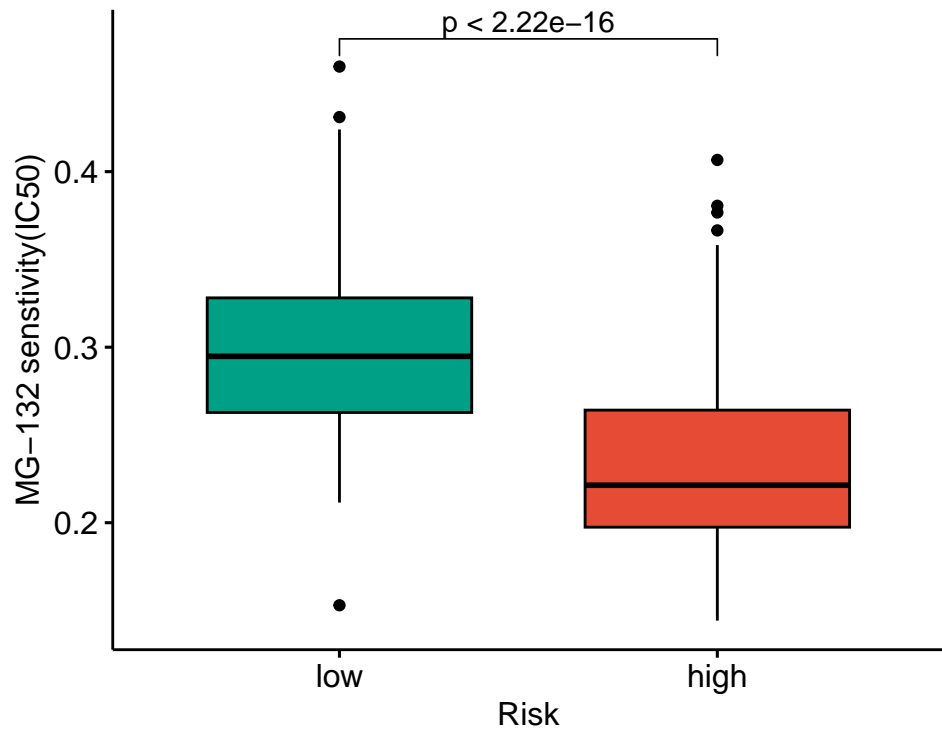

Supplement: Multimedia component 1 [file mmc1.zip › drug/drugSenstivity.MG-132.pdf]

Risk 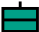 low 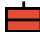 high

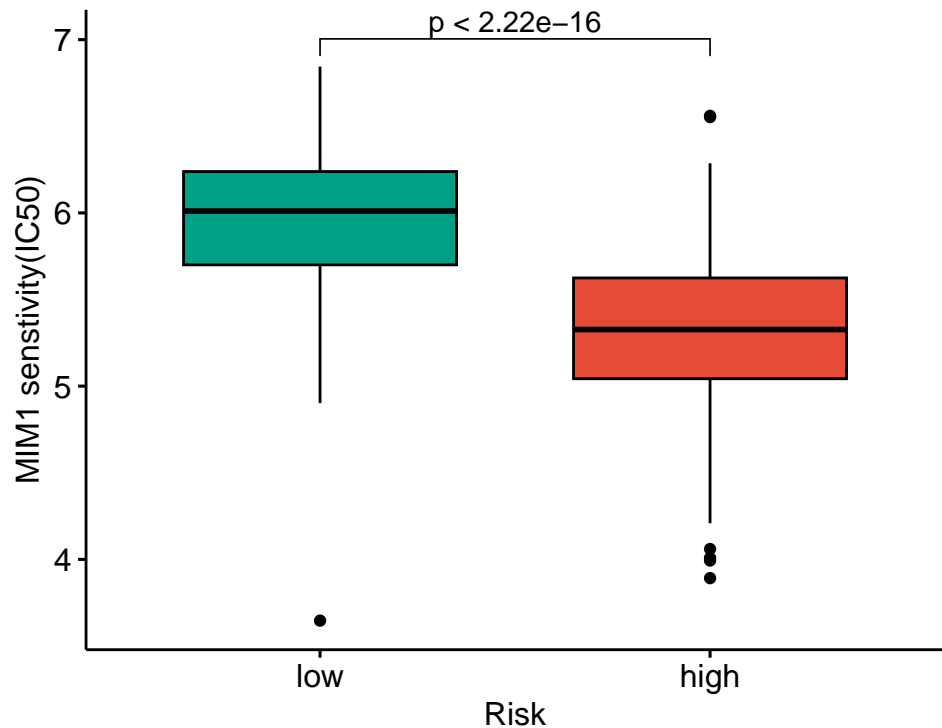

Supplement: Multimedia component 1 [file mmc1.zip › drug/drugSenstivity.MIM1.pdf]

Risk 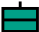 low 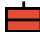 high

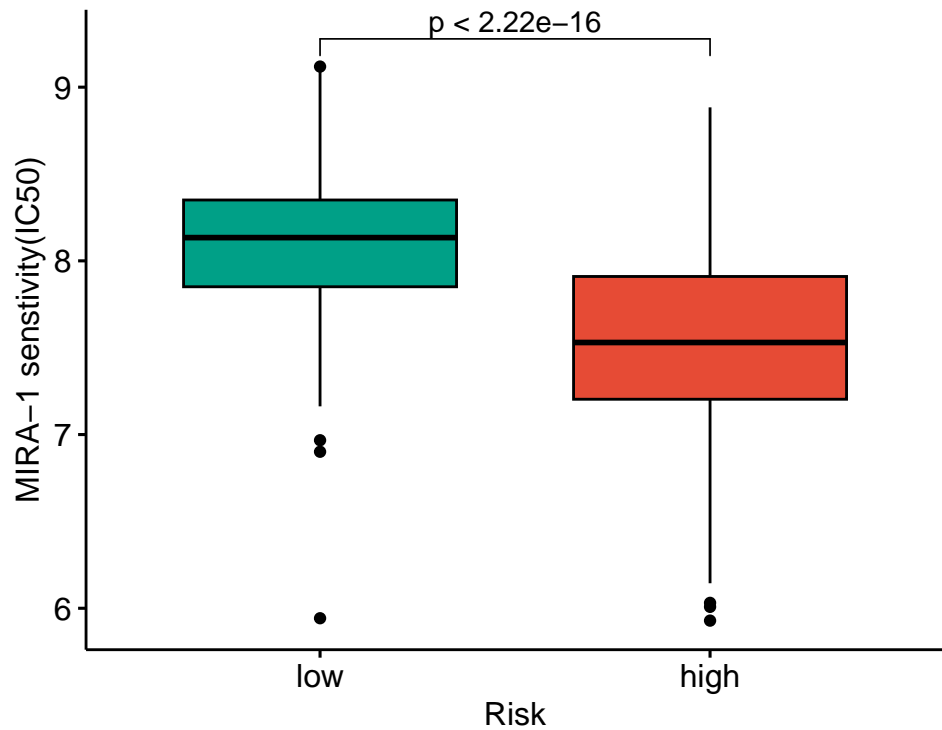

Supplement: Multimedia component 1 [file mmc1.zip › drug/drugSenstivity.MIRA-1.pdf]

Risk 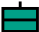 low 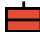 high

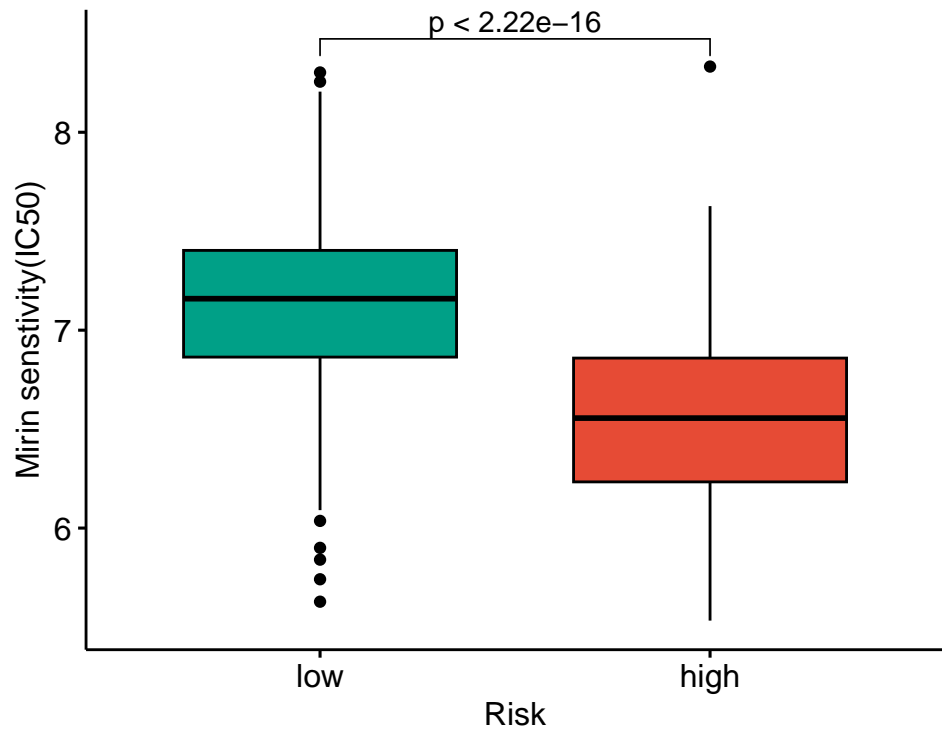

Supplement: Multimedia component 1 [file mmc1.zip › drug/drugSenstivity.Mirin.pdf]

Risk 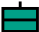 low 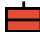 high

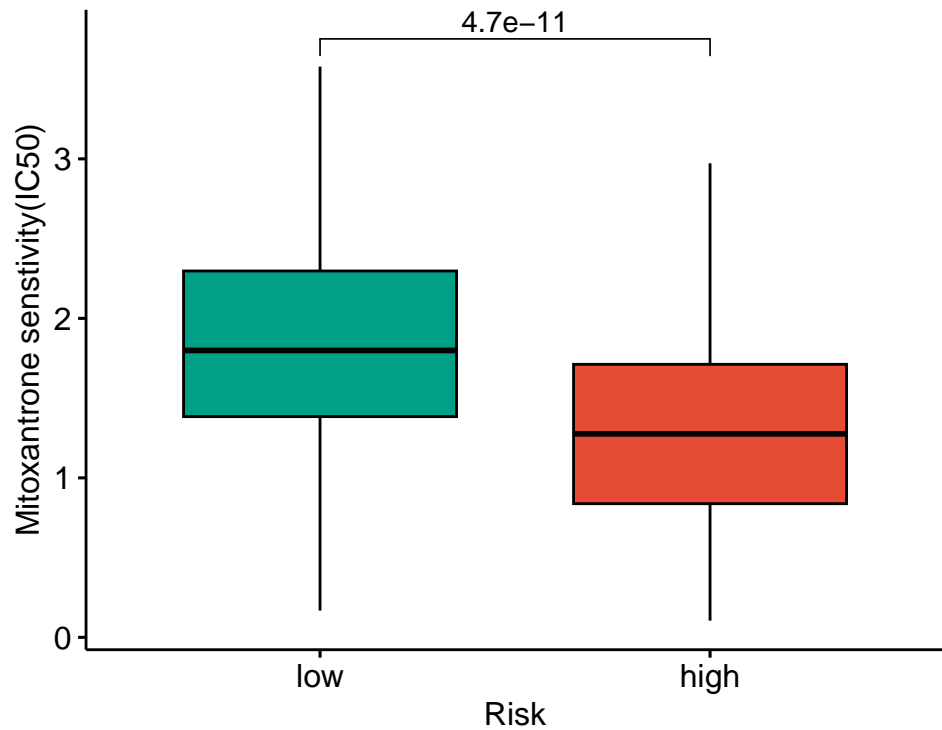

Supplement: Multimedia component 1 [file mmc1.zip › drug/drugSenstivity.Mitoxantrone.pdf]

Risk 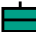 low 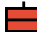 high

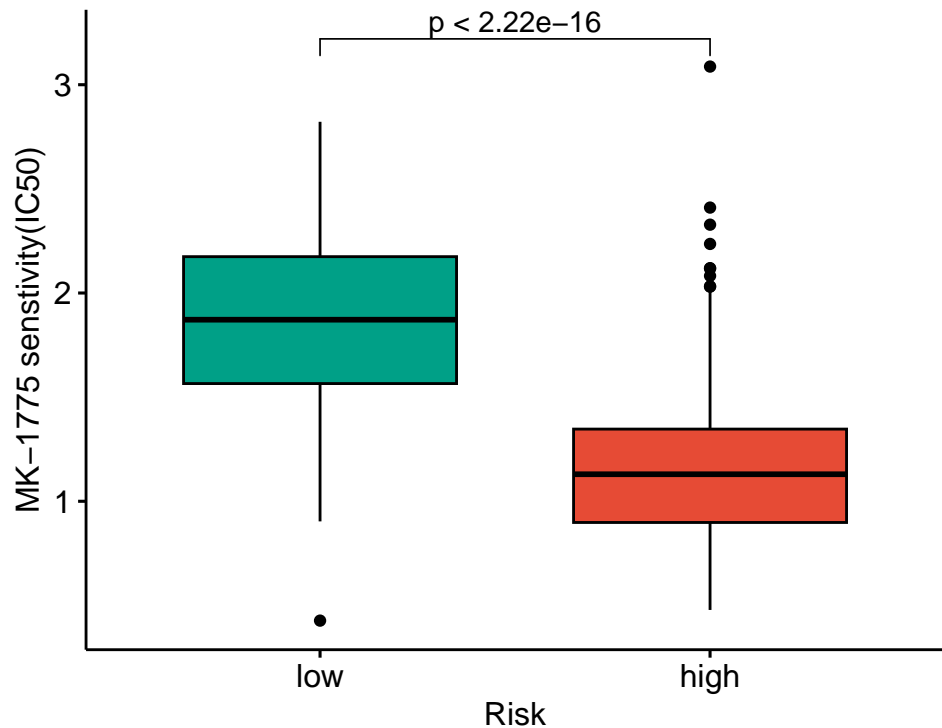

Supplement: Multimedia component 1 [file mmc1.zip › drug/drugSenstivity.MK-1775.pdf]

Risk 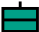 low 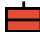 high

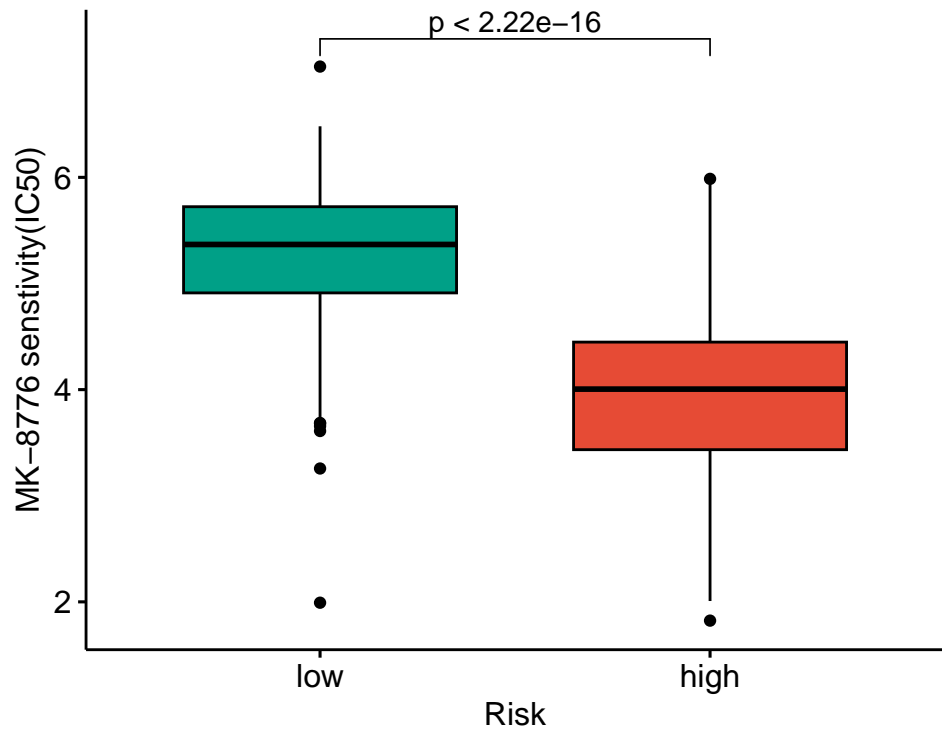

Supplement: Multimedia component 1 [file mmc1.zip › drug/drugSenstivity.MK-8776.pdf]

Risk 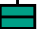 low 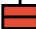 high

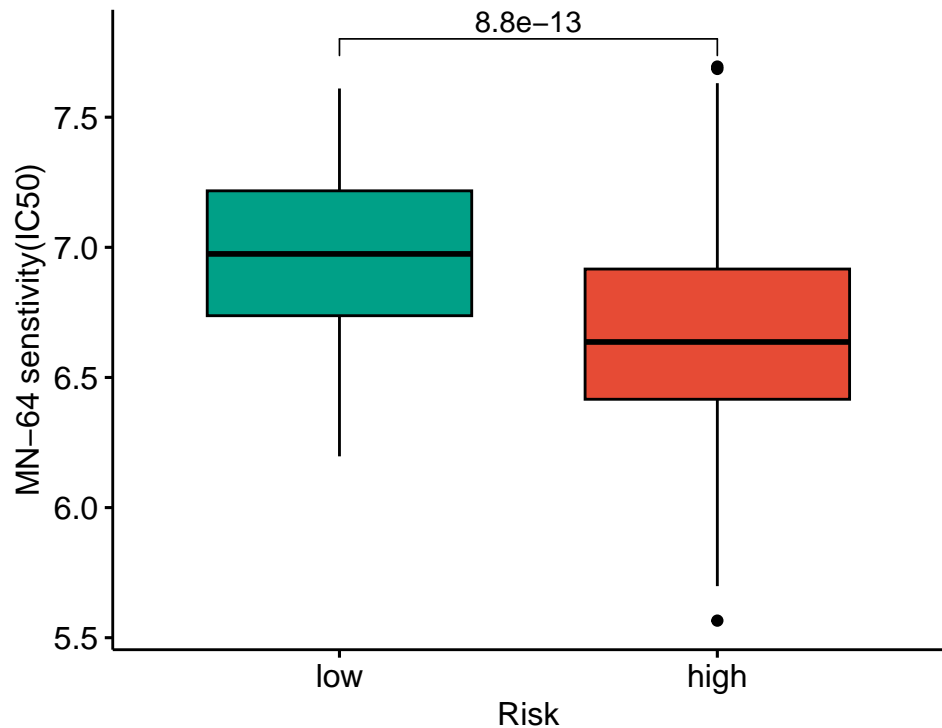

Supplement: Multimedia component 1 [file mmc1.zip › drug/drugSenstivity.MN-64.pdf]

Risk 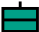 low 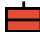 high

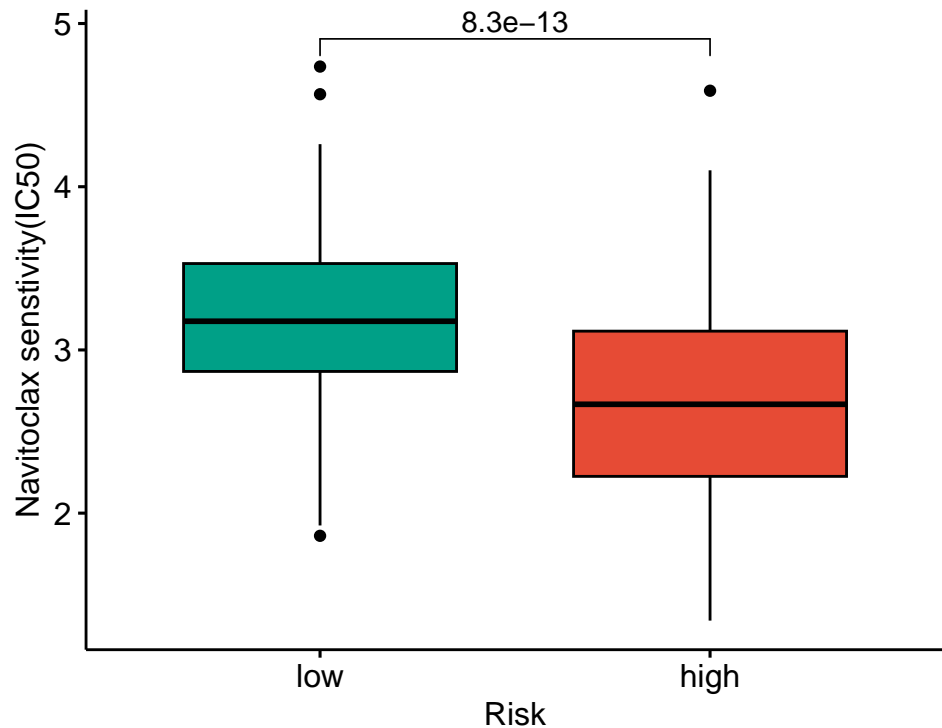

Supplement: Multimedia component 1 [file mmc1.zip › drug/drugSenstivity.Navitoclax.pdf]

Risk 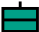 low 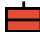 high

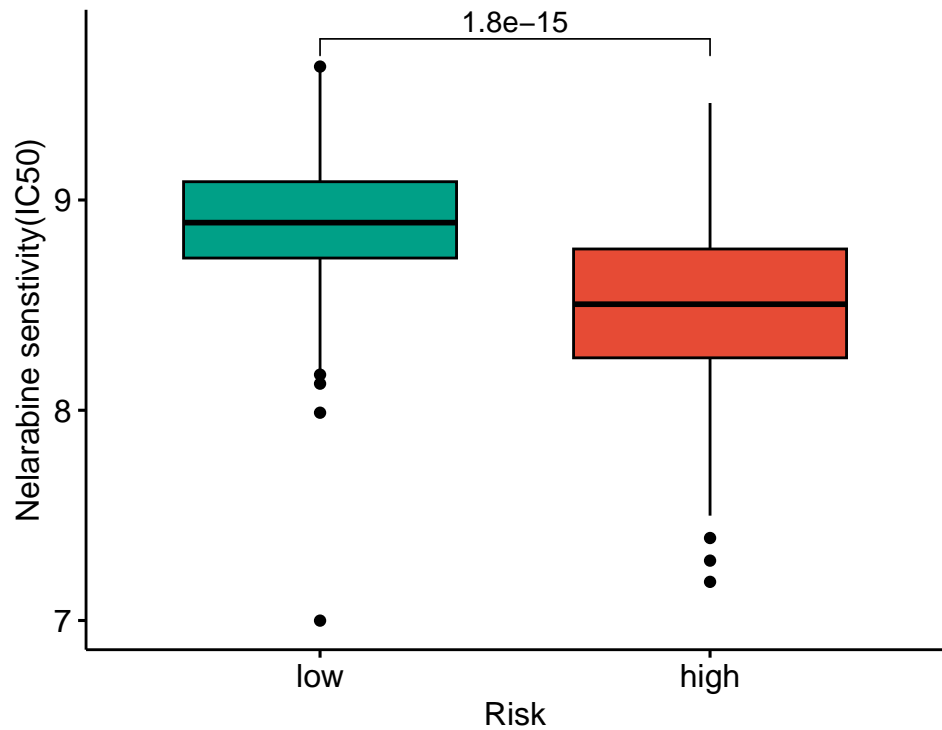

Supplement: Multimedia component 1 [file mmc1.zip › drug/drugSenstivity.Nelarabine.pdf]

Risk 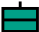 low 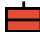 high

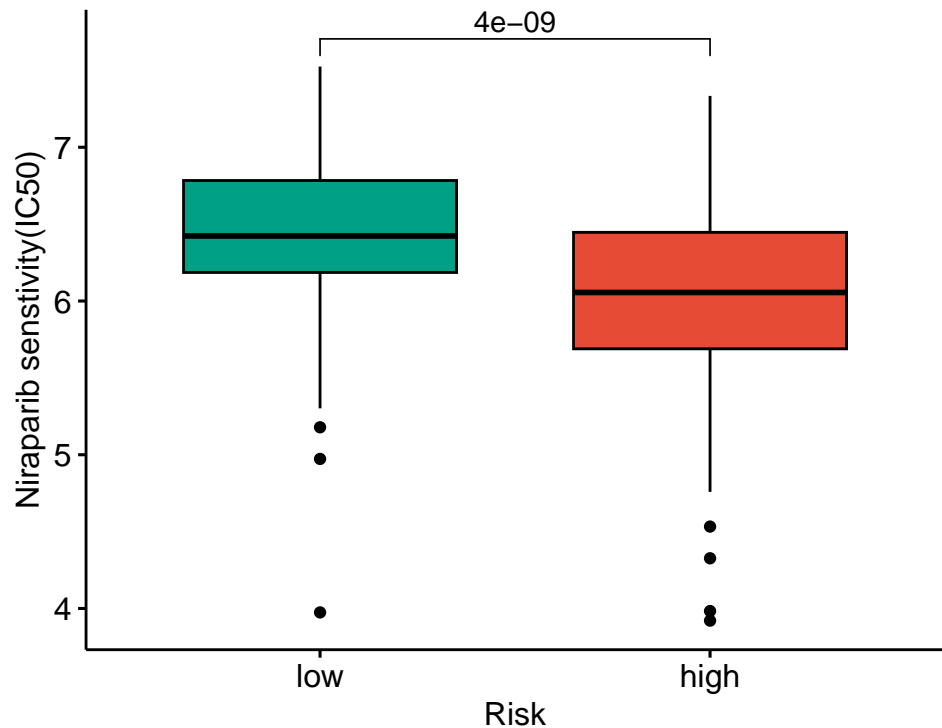

Supplement: Multimedia component 1 [file mmc1.zip › drug/drugSenstivity.Niraparib.pdf]

Risk 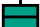 low 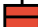 high

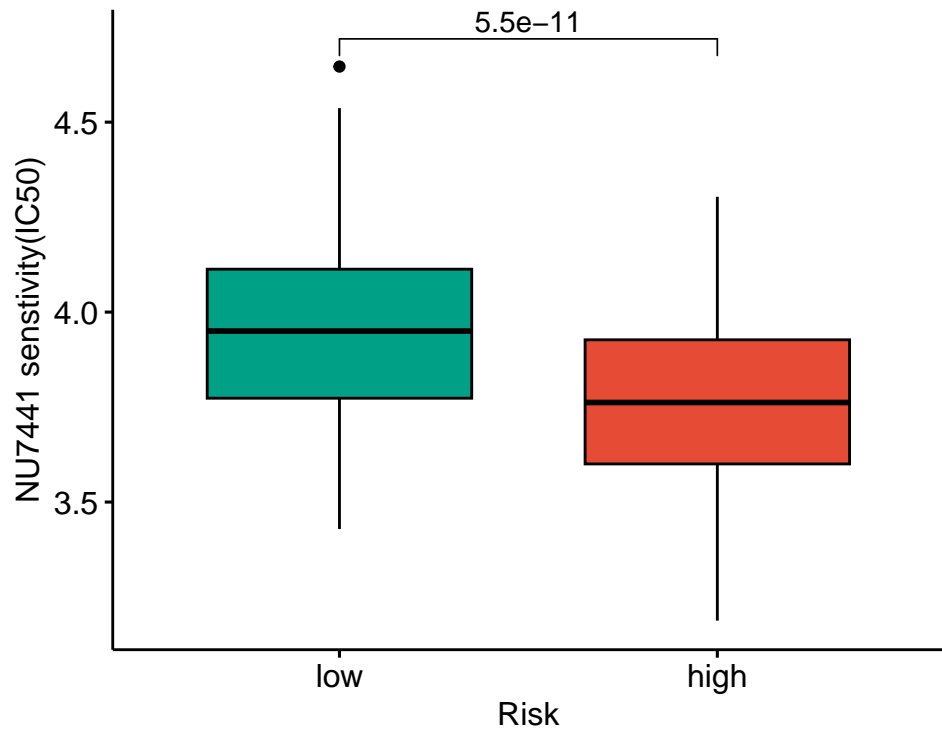

Supplement: Multimedia component 1 [file mmc1.zip › drug/drugSenstivity.NU7441.pdf]

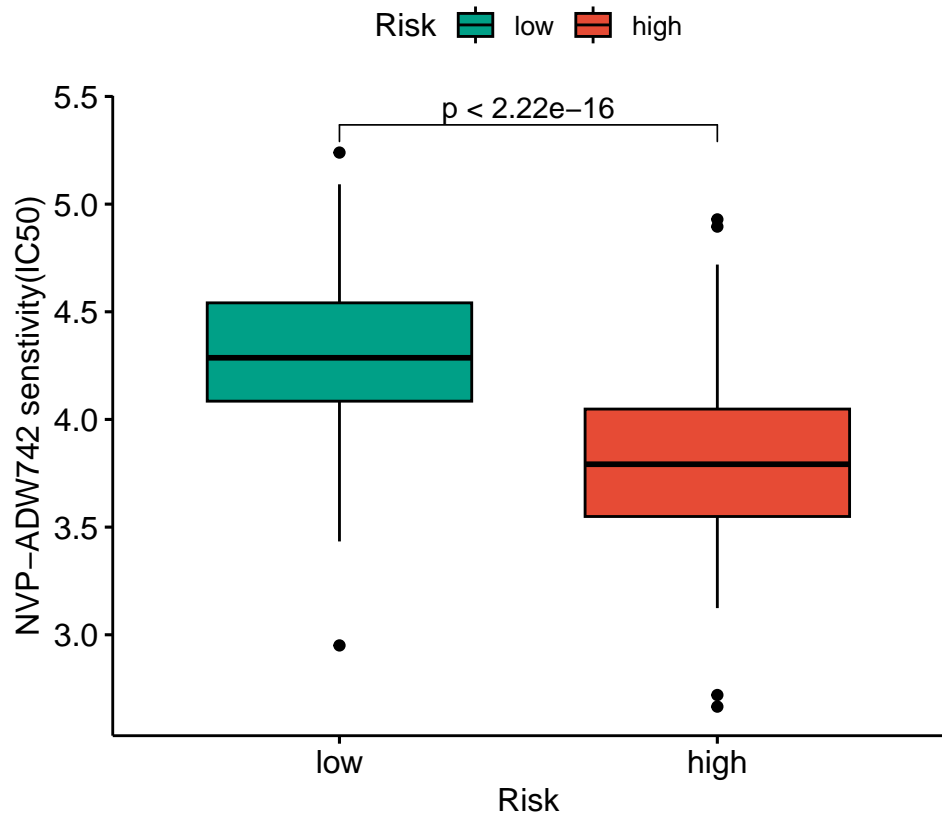

Supplement: Multimedia component 1 [file mmc1.zip › drug/drugSenstivity.NVP-ADW742.pdf]

Risk 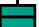 low 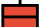 high

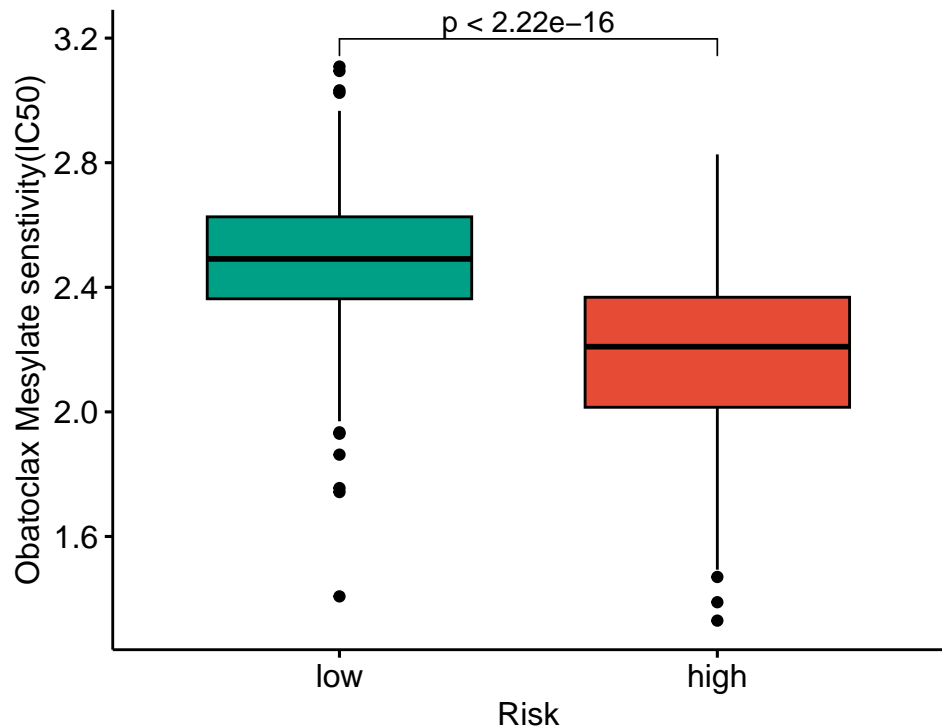

Supplement: Multimedia component 1 [file mmc1.zip › drug/drugSenstivity.Obatoclax Mesylate.pdf]

Risk 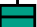 low 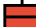 high

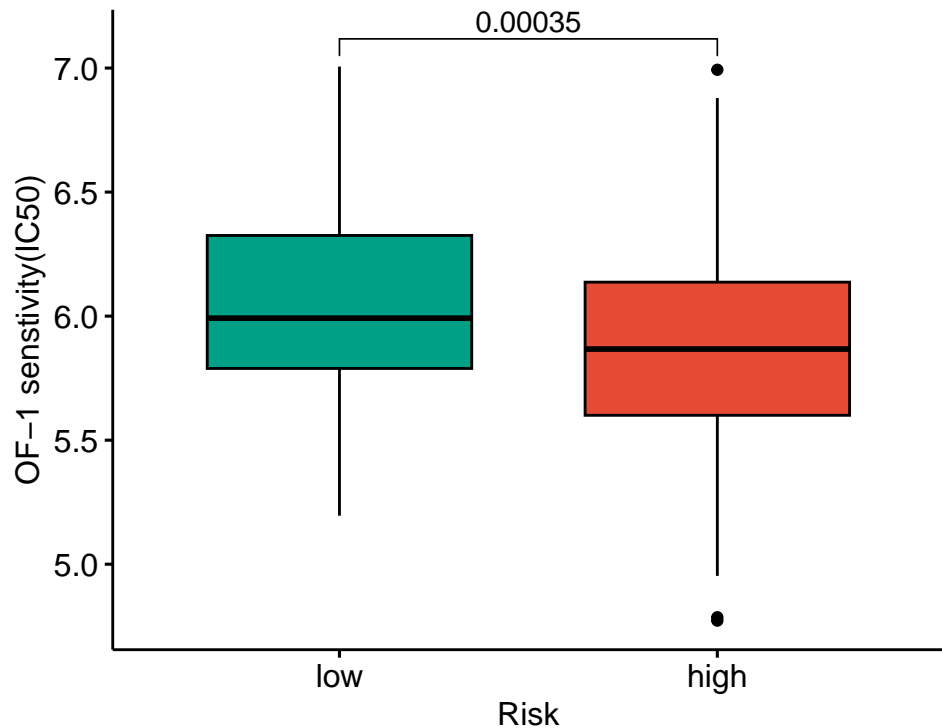

Supplement: Multimedia component 1 [file mmc1.zip › drug/drugSenstivity.OF-1.pdf]

Risk 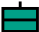 low 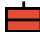 high

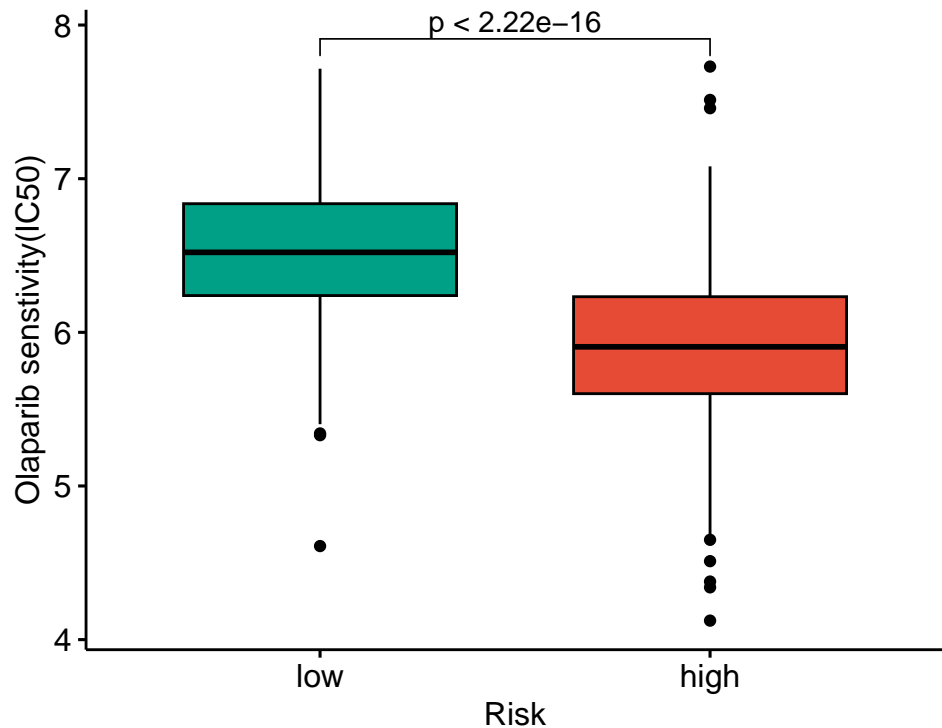

Supplement: Multimedia component 1 [file mmc1.zip › drug/drugSenstivity.Olaparib.pdf]

Risk 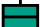 low 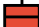 high

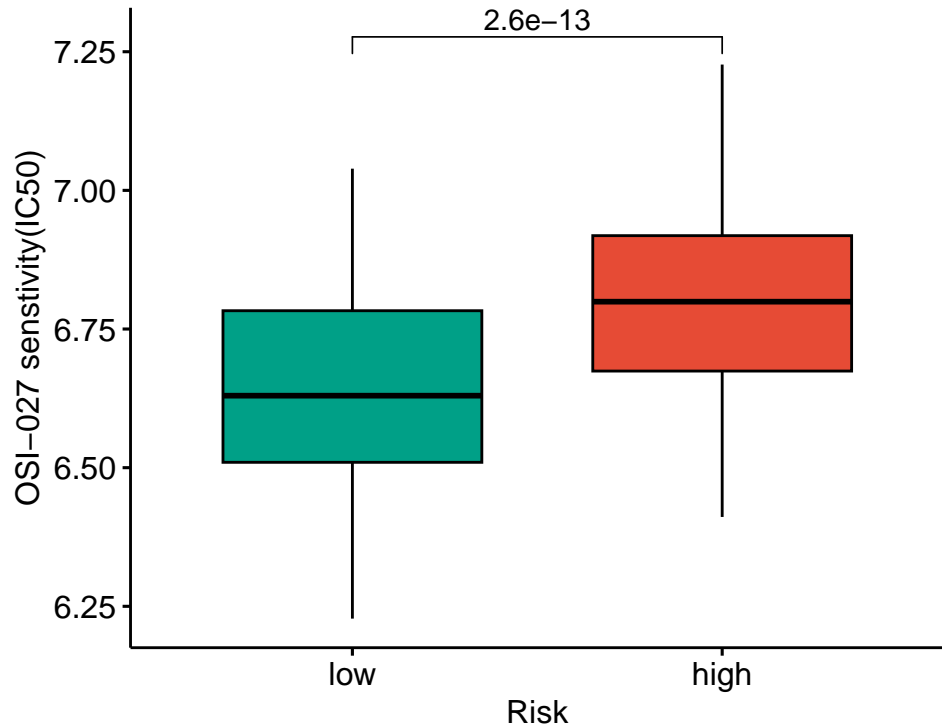

Supplement: Multimedia component 1 [file mmc1.zip › drug/drugSenstivity.OSI-027.pdf]

Risk 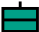 low 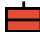 high

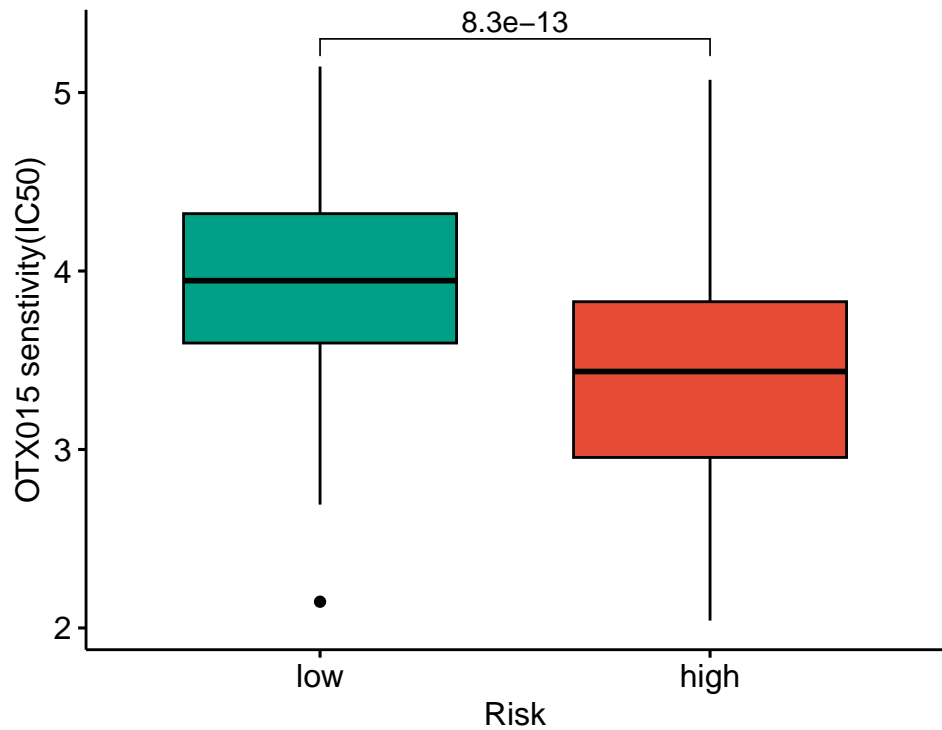

Supplement: Multimedia component 1 [file mmc1.zip › drug/drugSenstivity.OTX015.pdf]

Risk 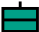 low 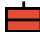 high

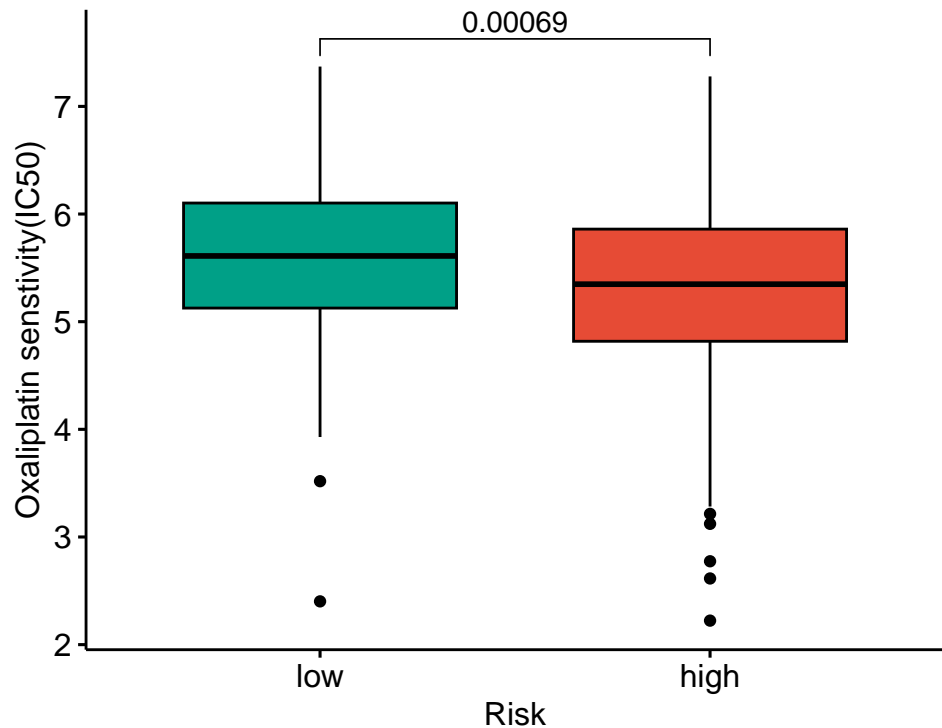

Supplement: Multimedia component 1 [file mmc1.zip › drug/drugSenstivity.Oxaliplatin.pdf]

Risk 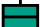 low 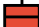 high

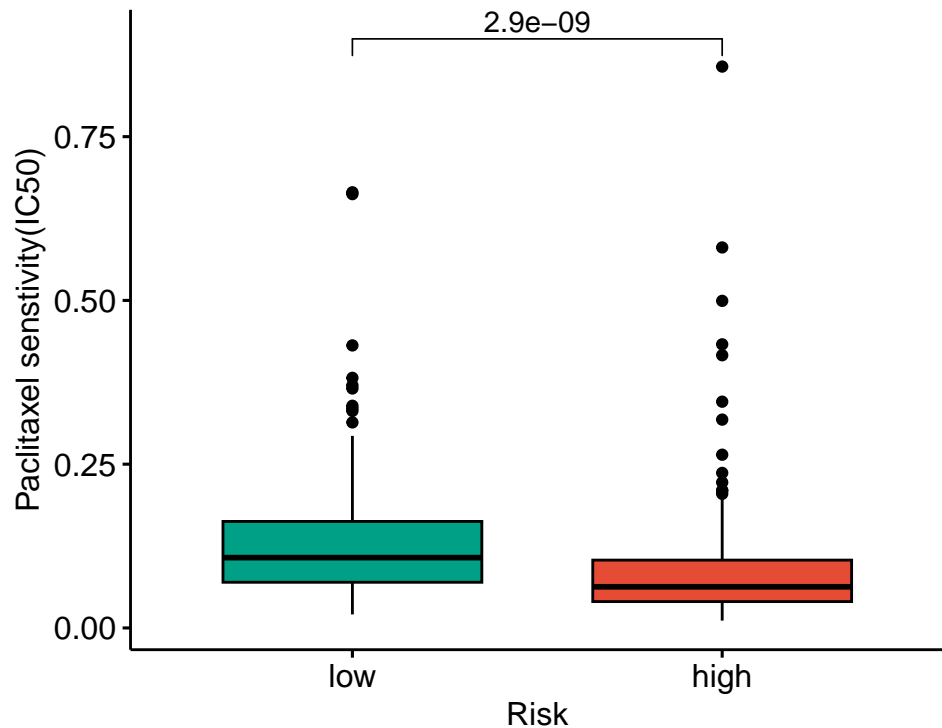

Supplement: Multimedia component 1 [file mmc1.zip › drug/drugSenstivity.Paclitaxel.pdf]

Risk 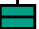 low 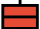 high

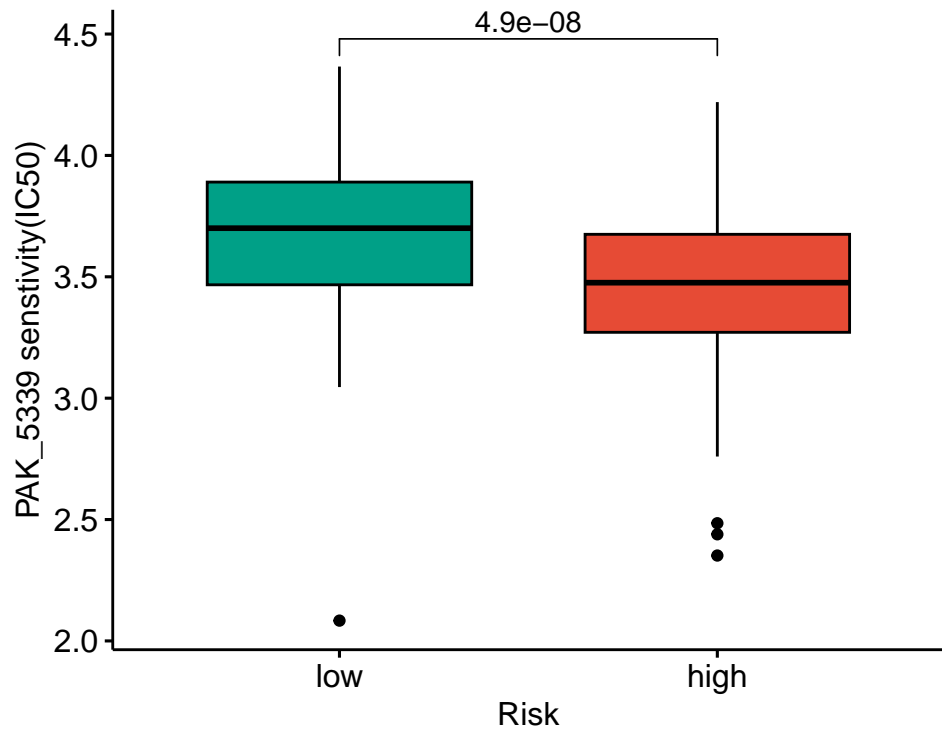

Supplement: Multimedia component 1 [file mmc1.zip › drug/drugSenstivity.PAK_5339.pdf]

Risk 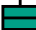 low 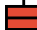 high

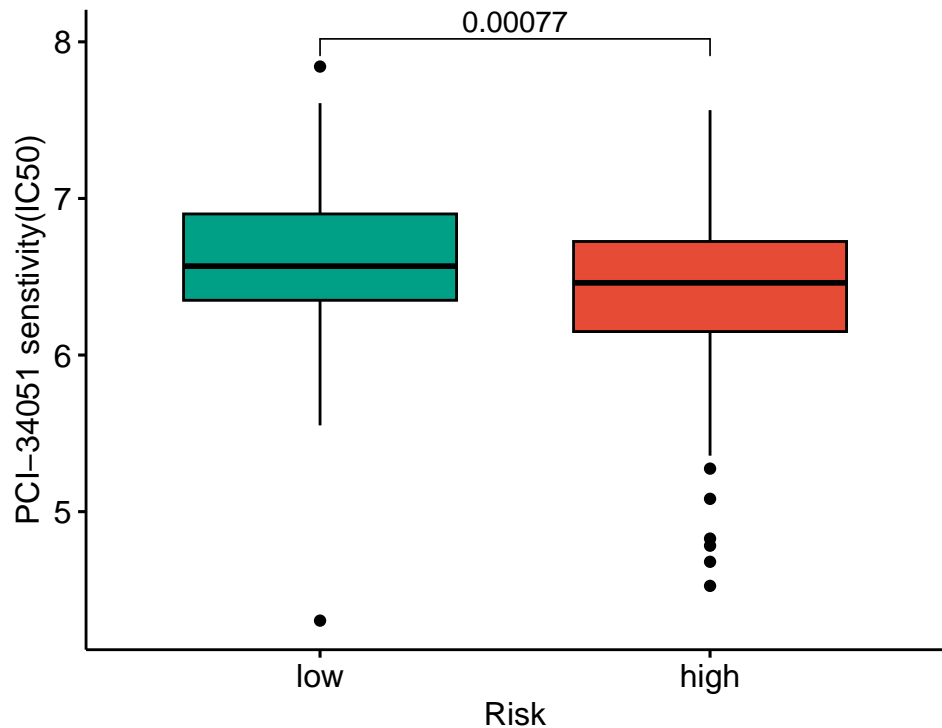

Supplement: Multimedia component 1 [file mmc1.zip › drug/drugSenstivity.PCI-34051.pdf]

Risk 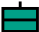 low 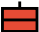 high

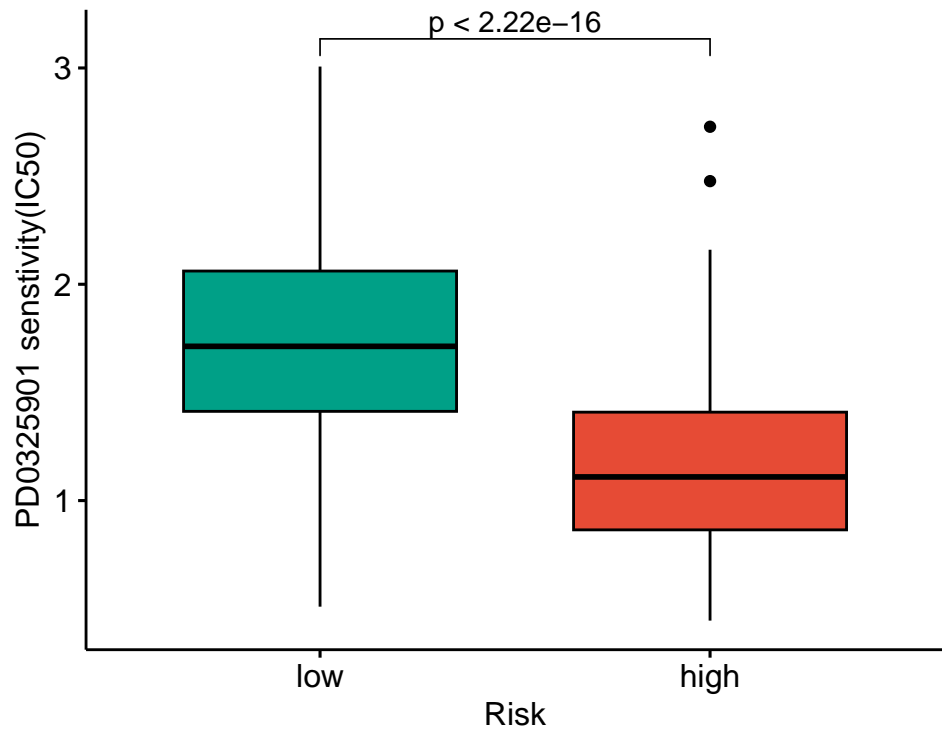

Supplement: Multimedia component 1 [file mmc1.zip › drug/drugSenstivity.PD0325901.pdf]

Risk 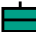 low 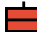 high

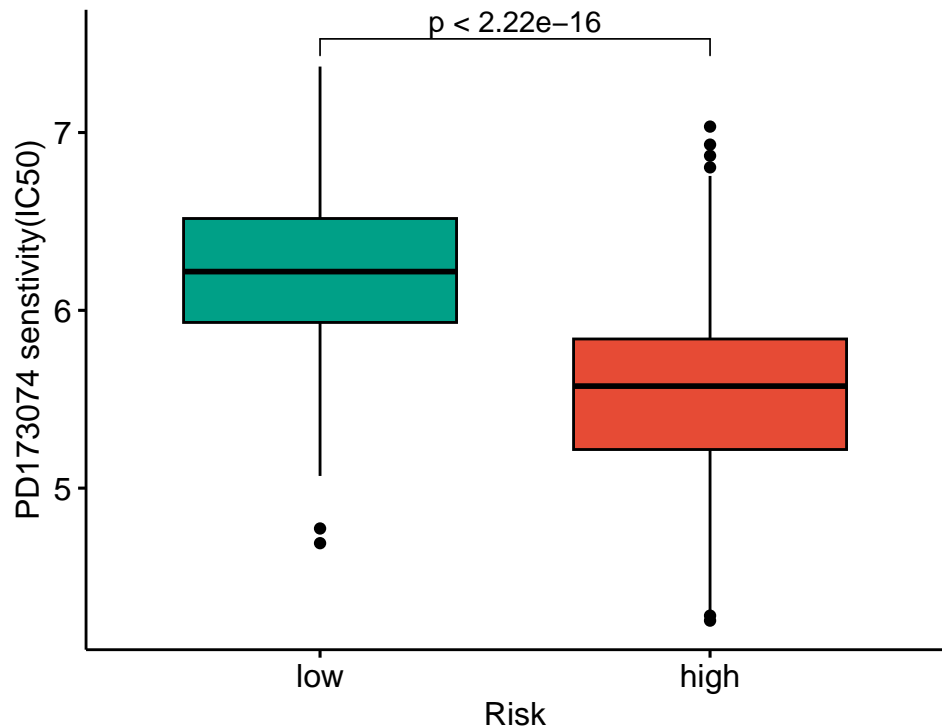

Supplement: Multimedia component 1 [file mmc1.zip › drug/drugSenstivity.PD173074.pdf]

Risk 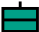 low 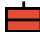 high

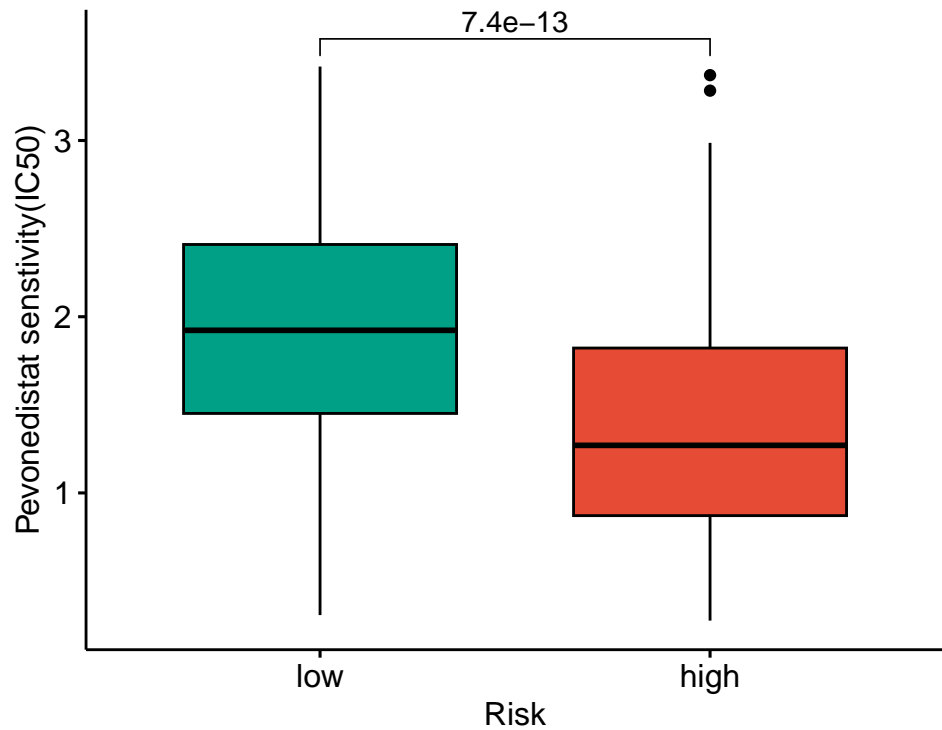

Supplement: Multimedia component 1 [file mmc1.zip › drug/drugSenstivity.Pevonedistat.pdf]

Risk 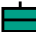 low 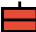 high

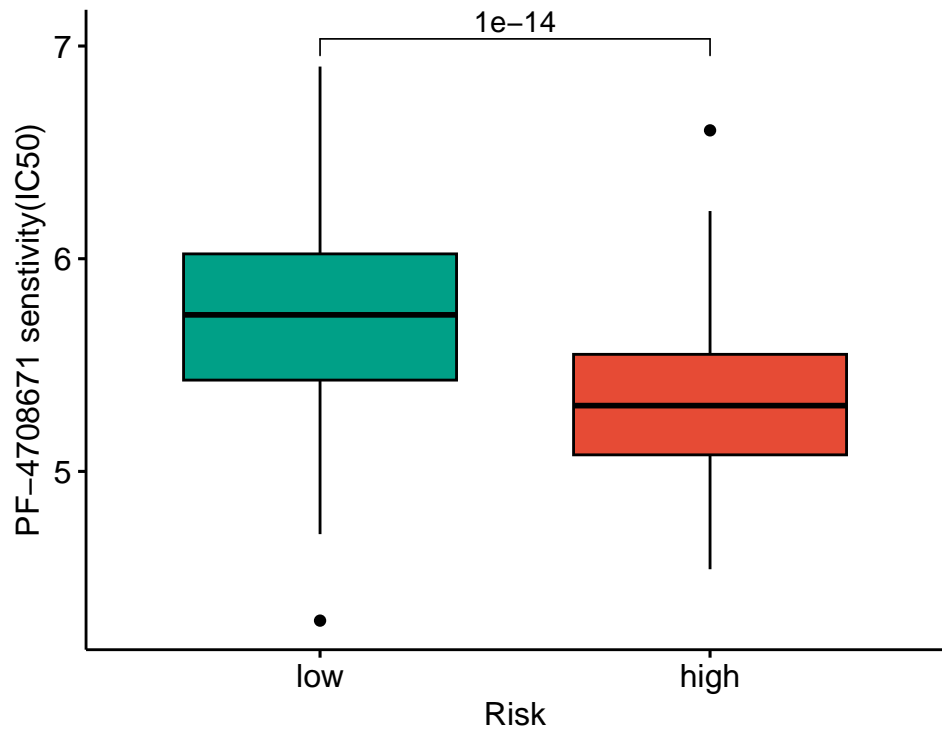

Supplement: Multimedia component 1 [file mmc1.zip › drug/drugSenstivity.PF-4708671.pdf]

Risk 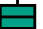 low 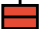 high

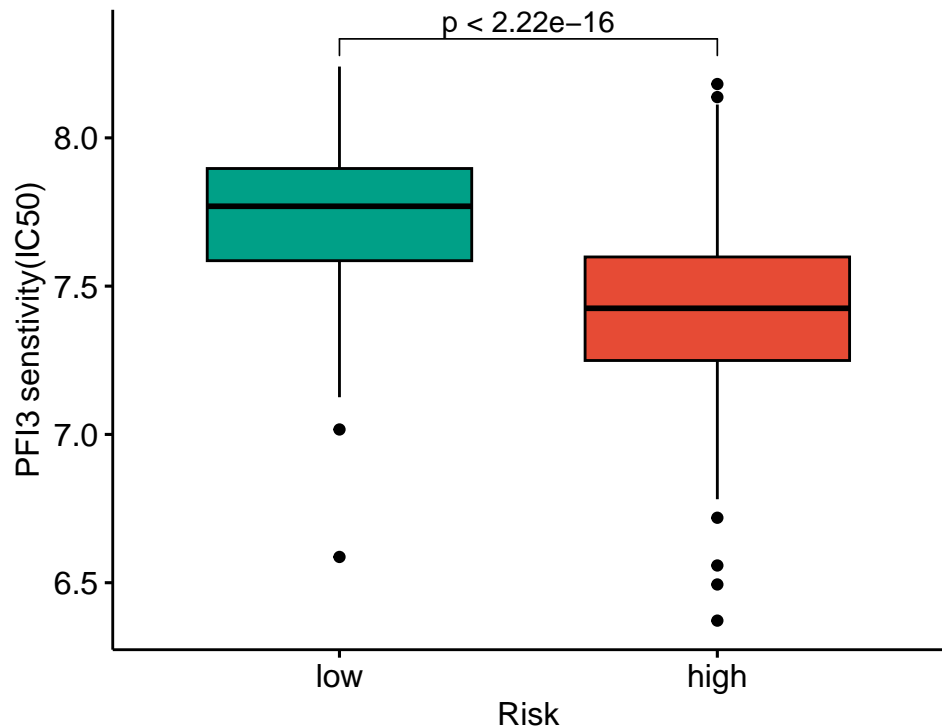

Supplement: Multimedia component 1 [file mmc1.zip › drug/drugSenstivity.PFI3.pdf]

Risk 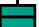 low 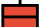 high

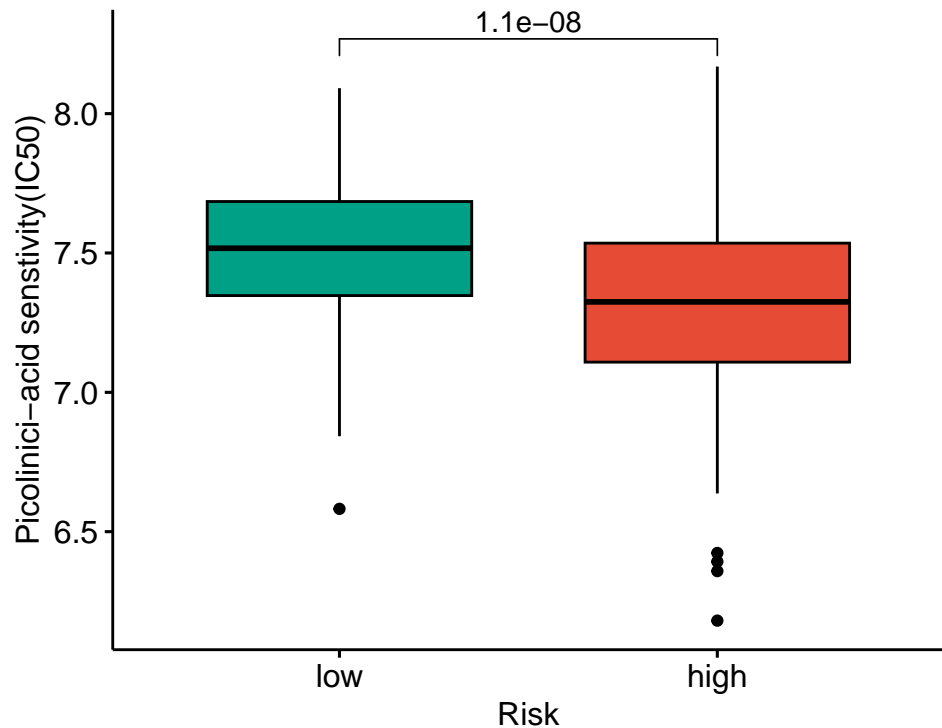

Supplement: Multimedia component 1 [file mmc1.zip › drug/drugSenstivity.Picolinici-acid.pdf]
